# Design, Synthesis, and Biological Evaluation of HDAC Inhibitors Containing Natural Product-Inspired *N*-Linked 2-Acetylpyrrole Cap

**DOI:** 10.3390/molecules29194653

**Published:** 2024-09-30

**Authors:** Han Zhang, Qianqian Shen, Zhu Hu, Pei-Qian Wu, Yi Chen, Jin-Xin Zhao, Jian-Min Yue

**Affiliations:** 1 School of Chinese Materia Medica, Nanjing University of Chinese Medicine, 138 Xianlin Road, Nanjing 210023, China; zhanghan@simm.ac.cn; 2 State Key Laboratory of Drug Research, Ethnomedicine and Biofunctional Molecule Research Center, Shanghai Institute of Materia Medica, Chinese Academy of Sciences, 555 Zuchongzhi Road, Shanghai 201203, China; huzhu@simm.ac.cn (Z.H.); wupeiqian@simm.ac.cn (P.-Q.W.); 3 State Key Laboratory of Chemical Biology, Division of Antitumor Pharmacology, Shanghai Institute of Materia Medica, Chinese Academy of Sciences, 501 Haike Road, Shanghai 201203, China; shenqianqian@simm.ac.cn; 4 Shandong Laboratory of Yantai Drug Discovery, Bohai Rim Advanced Research Institute for Drug Discovery, 198 East Binhai Road, Yantai 264117, China

**Keywords:** HDAC inhibitors, anti-cancer activity, natural products, pyrrole derivatives, molecular docking, molecular dynamics simulation

## Abstract

Drawing inspiration from the structural resemblance between a natural product *N*-(3-carboxypropyl)-2-acetylpyrrole and phenylbutyric acid, a pioneer HDAC inhibitor evaluated in clinical trials, we embarked on the design and synthesis of a novel array of HDAC inhibitors containing an *N*-linked 2-acetylpyrrole cap by utilizing the pharmacophore fusion strategy. Among them, compound **20** exhibited potential inhibitory activity on HDAC1, and demonstrated notable potency against RPMI-8226 cells with an IC_50_ value of 2.89 ± 0.43 μM, which was better than chidamide (IC_50_ = 10.23 ± 1.02 μM). Western blot analysis and Annexin V-FTIC/propidium iodide (PI) staining showed that **20** could enhance the acetylation of histone H3, as well as remarkably induce apoptosis of RPMI-8226 cancer cells. The docking study highlighted the presence of a hydrogen bond between the carbonyl oxygen of the 2-acetylpyrrole cap group and Phe198 of the HDAC1 enzyme in **20**, emphasizing the crucial role of introducing this natural product-inspired cap group. Molecular dynamics simulations showed that the docked complex had good conformational stability. The ADME parameters calculation showed that **20** possesses remarkable theoretical drug-likeness properties. Taken together, these results suggested that **20** is worthy of further exploration as a potential HDAC-targeted anticancer drug candidate.

## 1. Introduction

As one of the most serious threats to human health, cancer has become the leading cause of death worldwide after cardiovascular and cerebrovascular diseases [1,2]. Although great progress of chemotherapeutic agents has been made in cancer treatment, cancer-related deaths continue to dominate mortality due to drug resistance, which is the foremost reason for failure of cancer treatment [3,4,5,6,7]. Among the multiple contributing factors in cancer drug resistance, epigenetic dysregulation is one of the most important determinants [8]. Modification of epigenetic processes is considered to be an innovative strategy for cancer therapy [9,10]. Deacetylation of histone or non-histone catalyzed by histone deacetylases (HDACs) is one of the most intensively investigated epigenetic modifications [11,12].

**Figure 1:**
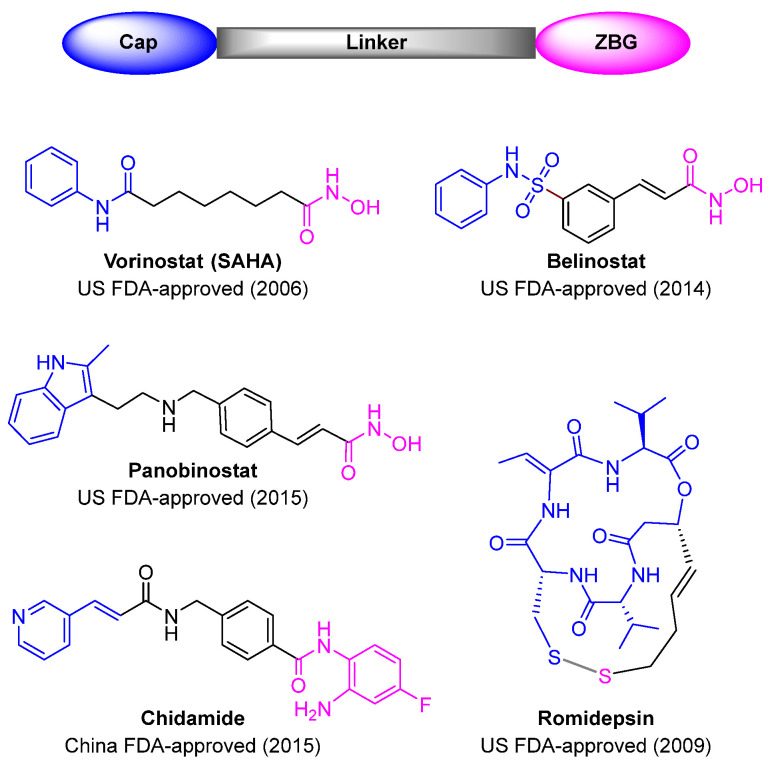
Chemical structures of currently approved HDAC inhibitors.

HDAC inhibitors (HDACis) have proved to be effective anticancer agents, owing to the potential capacity to inhibit cancer cell invasion, sensitize the cancer cells to chemotherapy, induce apoptosis, and boost immunogenicity [13]. To date, four HDACis, vorinostat (SAHA), belinostat, panobinostat, and romidepsin, have gained US Food and Drug Administration (FDA) approval for the treatment of refractory or relapsed cutaneous and peripheral T-cell lymphomas or multiple myeloma. Chidamide is another HDACi approved by the China FDA for the treatment of peripheral T-cell lymphoma (Figure 1) [14,15,16]. However, most HDACis are associated with adverse side effects, such as fatigue, nausea, thrombocytopenia, and neutropenia, prompting the search for new HDACis [4,17,18].

**Figure 2:**
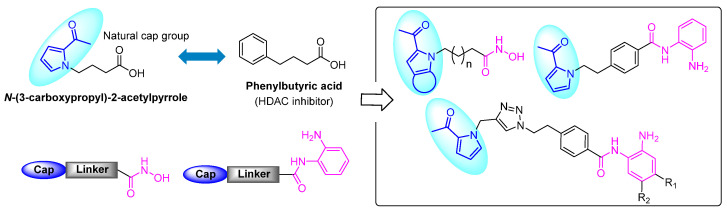
Design strategy and modification of novel HDAC inhibitors containing a natural product-inspired *N*-linked 2-acetylpyrrole cap.

Natural products are an important source of inspiration in drug design and development [19,20]. A short-chain aliphatic acid, *N*-(3-carboxypropyl)-2-acetyl pyrrole, isolated from *Sauropus spatulifolius* [21,22], captured our attention due to its highly similar structure to that of phenylbutyric acid (Figure 2), the first HDACi evaluated in clinical trials [23]. Pyrroles are among the most prevalent natural pharmacophores embedded in a number of drug molecules, such as ketorolac, atorvastatin, sunitinib, etc. [24,25], and they play a crucial role in the development of HDACis [26,27,28]. However, the utilization of a pyrrole motif as the cap group remains uncommon in existing works, where the linker chain is typically connected at the C-2 position [28,29,30]. Despite several reports utilizing indole as the cap group, only a limited number of HDACis possess the linker connection point on the nitrogen atom [4,11,31,32,33]. In the present work, we envisioned a new design strategy that integrates the natural product-derived 2-acetylpyrrole cap group with established HDACi pharmacophores. Leveraging this molecular hybridization strategy, two novel series of HDACis with hydroxamic acid or *o*-phenylenediamine as a ZBG have been successfully synthesized (Figure 2). Biological screening revealed that most of them exhibited nanomolar inhibitory activity against three HDAC isomers. Among them, compound **20** demonstrated the strongest anti-proliferative effects targeting RPMI-8226 cells. Herein, we report the discovery, biological evaluation, kinetic binding analysis, and ADME prediction of the synthesized HDACis.

## 2. Results and Discussion

### 2.1. Chemistry

**Scheme 1:**
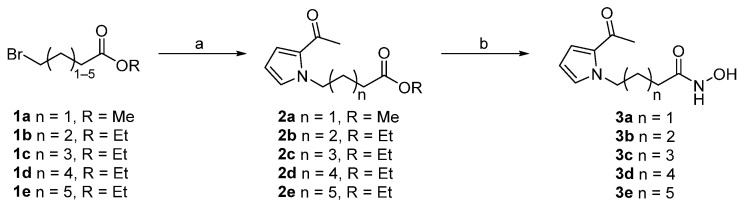
Reagents and conditions: (a) 2-acetyl pyrrole, NaH, DMF, 0 °C to rt; (b) NH_2_OH·HCl, KOH, MeOH, 0 °C to 40 °C.

**Scheme 2:**
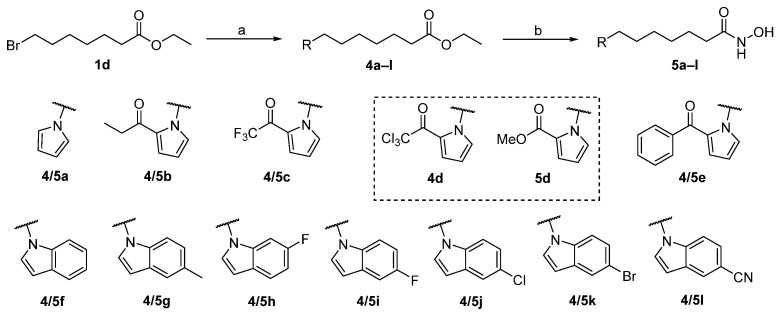
Reagents and conditions: (a) pyrrole or indole (RH), NaH, DMF, 0 °C to rt; (b) NH_2_OH·HCl, KOH, MeOH, 0 °C to 40 °C.

**Scheme 3:**
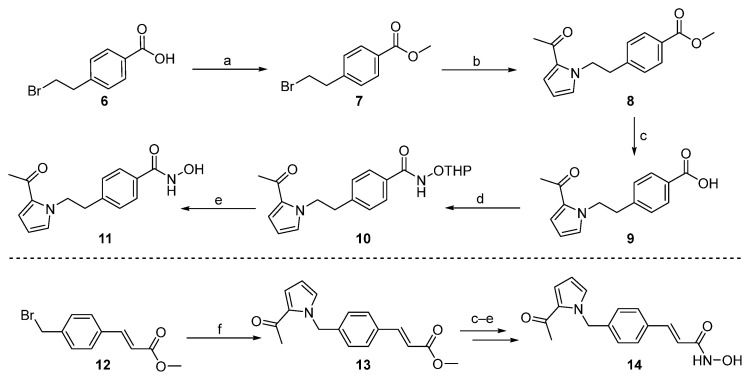
Reagents and conditions: (a) SOCl_2_, MeOH, 0 °C to reflux; (b) 2-acetylpyrrole, KOH, 18-crown-6, toluene, reflux; (c) LiOH, H_2_O/MeOH (1:4), 40 °C; (d) *O*-(tetrahydro-2*H*-pyran-2-yl)hydroxylamine, DIPEA, HATU, DCM, rt; (e) TsOH, MeOH, rt; (f) 2-acetylpyrrole, NaH, DMF, 0 °C to rt.

**Scheme 4:**
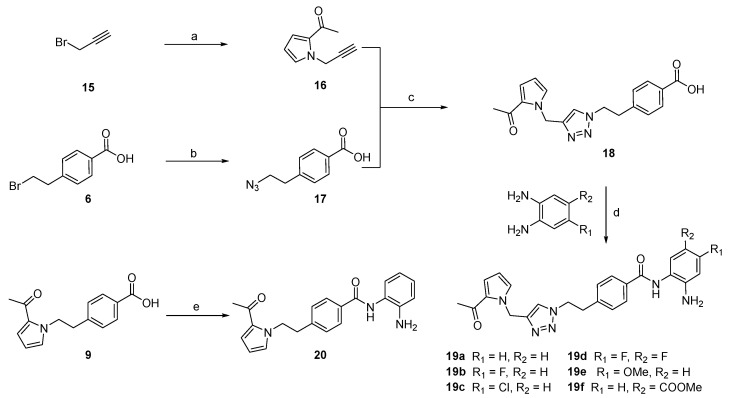
Reagents and conditions: (a) 2-acetylpyrrole, NaH, DMF, 0 °C to rt; (b) TBAF, TMSN_3_, THF, rt; (c) CuSO_4_·5H_2_O, sodium l-ascorbate, DMF/H_2_O (3:1), rt; (d) EDCI, DMAP, DCM, rt; (e) 1,2-diaminobenzene, DIPEA, HATU, DCM, rt.

Two series of target compounds were designed and prepared. One is with hydroxamic acid as a ZBG (Scheme 1, Scheme 2 and Scheme 3), and the other is with *o*-phenylenediamine (Scheme 4).

Firstly, we synthesized hydroxamic acid-based SAHA-like targeting products **3a**–**e** comprising aliphatic linkers of various lengths (Scheme 1). The reactions of commercially available bromo esters **1a**–**e** with 2-acetylpyrrole under basic conditions successfully provided intermediates **2a**–**e**. Next, compounds **2a**–**e** were converted into the target compounds **3a**–**e** in a single step by using hydroxyamine in the presence of potassium hydroxide. Through a similar two-step procedure, the target compounds **5a**–**l** were successfully synthesized from ethyl 7-bromoheptanoate (**1d**) (Scheme 2). Particularly, the 2-trichloroacetyl pyrrole derivative **4d** yielded the methyl ester **5d**. Two more target compounds **11** and **14** were synthesized by similar procedures from brominated starting materials 4-(2-bromoethyl)benzoic acid (**6**) and methyl (*E*)-3-(4-(bromomethyl)phenyl)acrylate (**12**), respectively (Scheme 3).

Next, we prepared a series of *o*-phenylenediamine-based targeting products (Scheme 4). Starting from propargyl bromide (**15**) and 4-(2-bromoethyl)benzoic acid (**6**), alkyne **16** and azide **17** were synthesized in a single step, respectively. Subsequent copper(II)-catalyzed click reaction afforded triazole intermediate **18**, which underwent amidation with various *o*-phenylenediamines to furnish target compounds **19a**–**f**. The advanced intermediate **9** was coupled with *o*-phenylenediamine to result in an additional target compound **20**.

### 2.2. In Vitro HDAC Inhibitory Activity and Structure-Activity Relationship

Upon completion of the synthesis, all target compounds **3a**–**e**, **5a**–**l**, **11**, **14**, **19a**–**f**, and **20** were evaluated against three HDAC isomers (HDAC1/3/6) (Table 1, Table 2 and Table 3). Among the hydroxamic acid-based targeting products **3a**–**e**, compound **3d** exhibited the best inhibitory activity, indicating that a six-methylene linker is optimal for the activity. Introduction of phenyl groups into the linker chain, as exemplified by **11** and **14**, resulted in a slight diminution of inhibitory activities (Table 1).

Based on these results, we preferentially introduced hydroxamic acid into the *N*-1 position of the pyrrole cap with a 6-carbon aliphatic linker (**5a**–**e**). Compound **5a** without any substituent on the pyrrole cap showed diminished inhibitory activity, implying that the 2-acyl group is pivotal in maintaining the activity (Table 2). Compound **5e** incorporating a 2-benzoylpyrrole cap exhibited comparable inhibitory effects to SAHA. Most of these compounds showed good selectivity towards the HDAC6 isomer. However, compound **5e** stood out as it displayed a slight preferential inhibition towards HDAC3 compared to HDAC1 and HDAC6. Replacing the 2-acyl pyrrole cap with an indole cap (**5f**–**l**) basically led to better inhibitory effects (Table 2). Both compounds **5i** and **5l** possessing a 5-fluoro and 5-cyano indole cap group, respectively, showed even stronger HDAC inhibition potency than SAHA.

Among the target compounds (**19a**–**f** and **20**) with an *o*-phenylenediamine cap group, only compounds **19a** and **20** showed certain inhibitory activity against HDAC1 and HDAC3 isoforms (Table 3). Substitution with the fluorine (**19b** and **19d**), chloro (**19c**), methoxy (**19e**), or ester group (**19f**) in the *o*-phenylenediamine ZBG leads to a loss of activity.

### 2.3. In Vitro Anti-Proliferation Against Cancer Cells

To assess the potential, those compounds with good HDAC inhibition (**3d**, **5a**–**l**, **11**, **14**, **19a**, and **20**) were evaluated for their anti-proliferation against three different tumor cell lines, HCT-116 (human colon cancer cell line), HL-60 (human leukemia cell line), and RPMI-8226 (human myeloma cell line) (Table 4). Surprisingly, among the hydroxamic acid-based HDACis, a decrease in antiproliferative activity of the indole derivatives (**5f**–**l**) was observed in contrast to their displayed potency against HDACs, and most pyrrole derivatives (**5a**, **5b**, **5d**, **5e**, and **11**) also exhibited weaker antiproliferative activity. On one hand, it may be attributed to off-target effects, as hydroxamic acid is known for its non-specific metal binding capability [34]. On the other hand, the hydroxamic acid group is susceptible to hydrolysis by esterases, as exemplified in trichostatin A [35,36]. In addition, hydroxamic acid is prone to form isocyanates by Lossen rearrangement, which consequently leads to the loss of its inhibitory activity [37,38]. In contrast, compounds **5c** and **14**, and two *o*-phenylenediamine-based HDACis, **19a** and **20**, showed relatively good inhibitory effects, and the anti-proliferative activity of **20** on RPMI-8226 cells was even better than that of the positive control chidamide. After a thorough analysis of the aforementioned information, we chose compounds **5c**, **14**, **19a**, and **20** for in-depth evaluation to elucidate their efficacy and mechanism on the cellular level.

### 2.4. Analysis of Ac-Tubulin and Ac-Histone H3 Levels in RPMI-8226 Cells by Western Blotting

To firmly establish the inhibitory effects of compounds **5c**, **14**, **19a**, and **20** on HDAC deacetylation activity, we performed a meticulous assessment of their impact on tubulin and histone H3 acetylation levels in RPMI-8226 cells.

**Figure 3:**
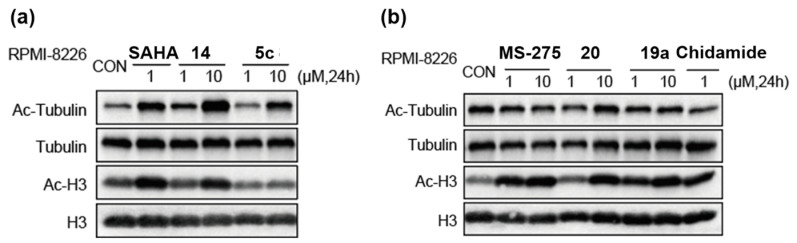
Western blot analysis of Ac-tubulin and Ac-histone H3 from RPMI 8226 cells cultured for 24 h with DMSO control and selected compounds. (**a**) Effect of compounds **5c**, **14**, and SAHA on tubulin and histone H3 acetylation; (**b**) effect of compounds **19a**, **20**, MS-275, and chidamide on tubulin and histone H3 acetylation.

As shown in Figure 3a, compound **14** induced more acetylated tubulin and acetylated histone H3 at the same concentration compared to compound **5c**, aligning with its stronger anti-proliferation effects on cancer cells (Table 4). At a concentration of 10 μM, compounds **20** and **19a** increased histone H3 acetylation levels comparable to MS-275. HDAC6 is primarily located in the cytoplasm and deacetylates cytoplasmic non-histone substrates, such as α-tubulin, to regulate tumor cell proliferation and apoptosis [38]. In Figure 3b, compounds **20**, **19a**, MS-275, and chidamide at tested concentrations had no discernible effect on Ac-α-tubulin protein expression associated with HDAC6 activity. This result confirms the selectivity of compounds **20** and **19a** observed in the isoform profiling (Table 3).

### 2.5. Effects of Compounds **5c**, **14**, **19a**, and **20** on Cancer Cell Apoptosis

**Figure 4:**
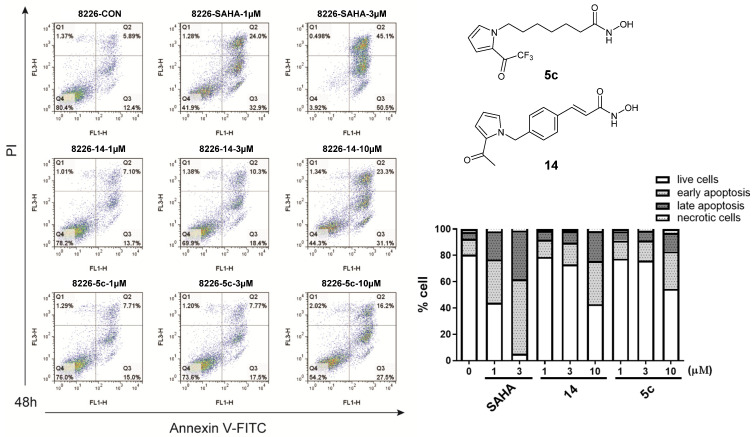
RPMI 8226 cells were treated with compounds **14** (1, 3, 10 μM), **5c** (1, 3, 10 μM), and SAHA (1, 3 μM) and DMSO (0.1%) for 48 h, and then stained with Annexin-V-FITC and propidium iodide (PI) and analyzed by flow cytometry.

**Figure 5:**
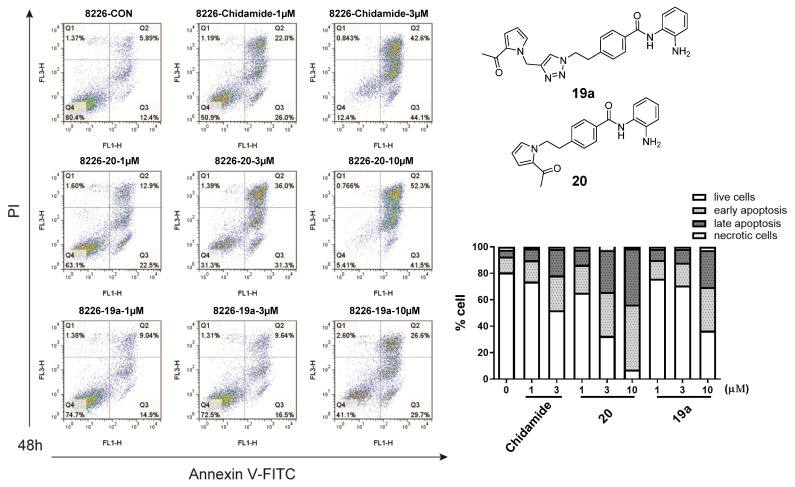
RPMI 8226 cells were treated with compounds **20** (1, 3, 10 μM), **19a** (1, 3, 10 μM), and chidamide (1, 3 μM) and DMSO (0.1%) for 48 h, and then stained with Annexin-V-FITC and propidium iodide (PI) and analyzed by flow cytometry.

To investigate whether the antiproliferative activities of compounds **5c**, **14**, **19a**, and **20** are accompanied by enhanced cancer cell apoptosis, a flow cytometry assay was implemented. RPMI-8226 cells were treated with selected compounds at concentrations of 1, 3, and 10 μM for 48 h, respectively, then stained with Annexin V-FTIC/propidium iodide (PI), and finally analyzed by flow cytometry. The flow cytometry data are shown in Figure 4 and Figure 5. Four tested compounds (**20**, **19a**, **14**, and **5c**) could promote RPMI-8226 cell apoptosis in a dose-dependent manner. Remarkably, compared with chidamide, compound **20** at 1 μM and 3 μM induced apoptosis in RPMI-8226 cells more effectively.

### 2.6. Molecular Docking of Compounds **19a** and **20** with HDAC1

Based on the protein crystal structure (PDB code: 1C3S), an in silico docking study was carried out to investigate the interaction of compounds **19a** and **20** with the active site of HDAC1 [39].

**Figure 6:**
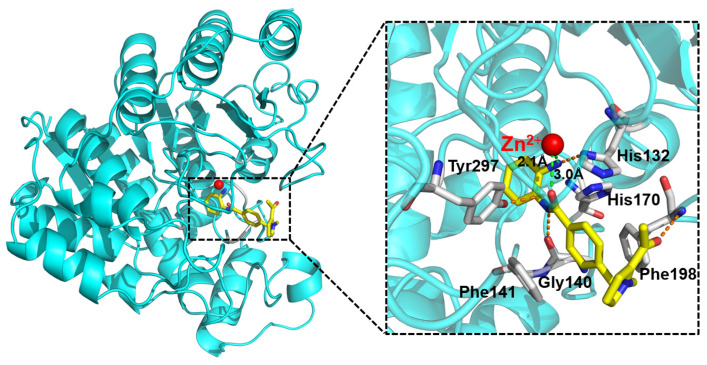
Binding pose of **20** with HDAC1 predicted by molecular docking (PDB entry: 1C3S). The figure was generated using PyMol (http://www.pymol.org/, accessed on 7 May 2024). Dash green lines represent the chelation of ZBG and zinc ions; Dash orange lines represent hydrogen bonding.

**Figure 7:**
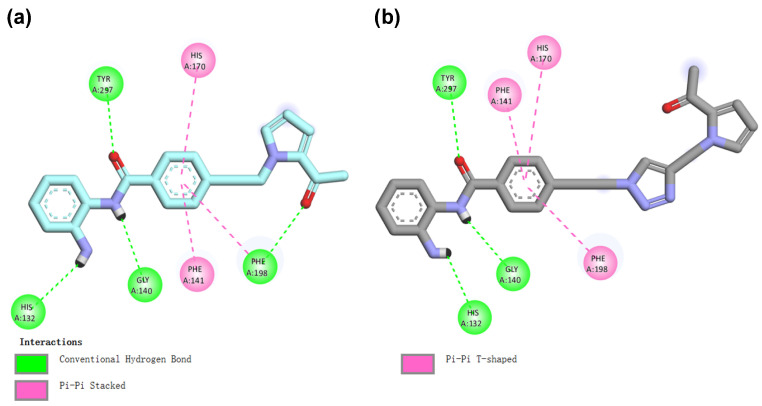
Predicted binding modes of compounds **20** (**a**) and **19a** (**b**) with HDAC1. The 2D figure was generated using Discovery Studio 4.5.

The docking results revealed that compound **20** could effectively penetrate the active pocket of the protein (Figure 6). The *N*-(2-aminophenyl)benzamide group of compound **20** chelated the Zn^2+^ ion well in a bidentate fashion, maintaining a distance of 3.0 Å to the oxygen atom in the carbonyl group and 2.1 Å to the nitrogen atom in aniline, respectively. In addition, the *N*-(2-aminophenyl)benzamide group also formed hydrogen bonding interactions with His132, Gly140, and Tyr297, respectively. The carbonyl oxygen of the 2-acetylpyrrole cap group formed a hydrogen bond with Phe198. The phenyl group within the linker fit well into the hydrophobic channel, engaging in π–π interactions with Phe198 and Phe141 in a face-to-face configuration, and with His170 in an edge-to-face orientation (Figure 6 and Figure 7a). For comparison, in the binding model of compound **19a** with HDAC1, the introduction of triazole resulted in a displacement and flip of the 2-acetylpyrrole group towards the outer solvent region, thereby abolishing the hydrogen bonding interaction with Phe198 and subsequently reducing the affinity (Figure 7b). In summary, molecular docking revealed that compound **20** interacts more closely with HDAC1, thus further validating the experimental findings.

### 2.7. Molecular Dynamic Simulation

Molecular dynamics (MD) simulations have been performed to elucidate the behavior of the HDAC1 enzyme upon inhibitor binding, and the stability and interactions of the structure throughout the simulations. Root mean square deviation (RMSD) represents the sum of all atomic deviations between the conformation and the target conformation at a certain time, and serves as a crucial benchmark to measure the stability of the system [40]. The root mean square fluctuation (RMSF) is utilized to assess the macromolecular flexibility, highlighting localized structural changes within the protein [41].

**Figure 8:**
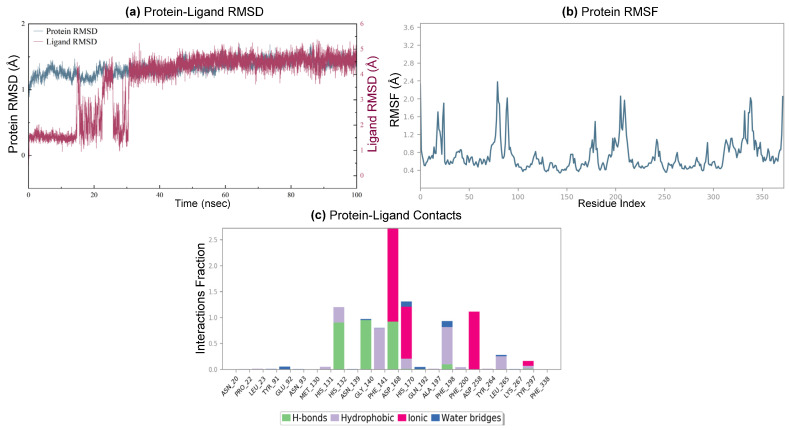
Molecular dynamics simulation of the docked complex of **20**-HDAC1. (**a**) RMSD of HDAC1 protein backbone atoms (blue line) and **20** (red line) monitored throughout the 100 ns; (**b**) RMSF of HDAC1 residue; (**c**) histogram showing interaction fractions.

Figure 8a depicts the RMSD values of the protein backbone atoms during the MD simulation, revealing that the **20**-HDAC1 complex attains a stable RMSD value after approximately 30 ns of simulation. As shown in Figure 8b, the RMSF data suggested a flexibility ranging from 0.4 to 2.4 Å, indicating that the residues within the binding pocket are relatively stable. Subsequently, protein interactions with the ligand are monitored throughout the simulation (Figure 8c). Zn^2+^ ion was penta-coordinated with Asp168, His170, and Asp258 as well as the carbonyl and aniline of benzamide of compound **20**. Hydrogen bonds are formed with His132, Gly140, Asp168, and Phe198. Moreover, hydrophobic contacts were observed with His132, Phe141, His170, Phe198, and Leu265, during the entire MD simulation process.

### 2.8. ADME Analysis

The ADME parameters (physicochemical properties, lipophilicity, pharmacokinetics, drug-likeness, and medicinal chemistry) of compound **20** and chidamide were measured using the SwissADME free web tool (http://www.swissadme.ch, accessed on 10 March 2024) [42,43,44]. The results are summarized in Table 5. Regarding the druglike properties, it was observed that **20** adheres to Lipinski’s rule of five and does not violate any Ghose, Muegge, Egan, or Veber filters. Besides, compound **20** exhibited a topological polar surface area (TPSA) of 77.12 Å^2^, suggesting that it could effectively permeate cell membranes (See Supplementary Materials Figure S1 for details).

**Figure 9:**
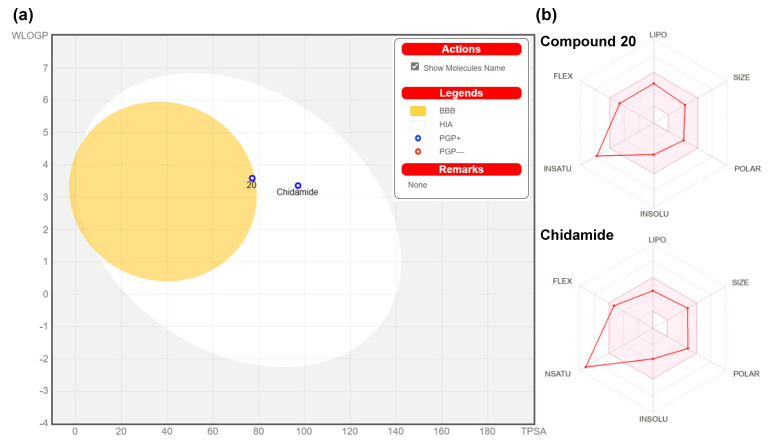
Boiled-Egg predictive model (**a**) and bioavailability radar (**b**) for **20** and chidamide. The white area is a high-probability area for passive absorption (HIA) in the gastrointestinal tract, and the yellow area (yolk) is a high-probability area for the blood-brain barrier (BBB). The yolk and white zone are not mutually exclusive. Points are shown in blue if predicted to be an active P-gp efflux (PGP+) and in red if predicted to be a non-P-gp substrate (PGP−).

The Boiled-Egg model of **20** shows high gastrointestinal (GI) absorption, indicating potentially good overall oral bioavailability (Figure 9a). Furthermore, **20** is predicted as passively traverse the brain penetration (BBB, at the yolk edge), but it can be pumped out of the brain by P-glycoprotein (P-gp, blue dot), thereby reducing its absorption and penetration into the brain [42,45]. As depicted in Figure 9b, the bioavailability radar of compound **20** also highly resembles that of chidamide.

### 2.9. Preliminary Pharmacokinetic Profiling

Subsequently, a pharmacokinetic study of compound **20** was conducted in male ICR mice. The pharmacokinetic profiles are summarized in Table 6. The results reveal that it has a high clearance of 102 mL/min/kg and a terminal half-life (*t*_1/2_) of 0.192 h after intravenous (iv) route at 1 mg/kg dose. Although the oral bioavailability was lower than expected due to lower plasma exposure (*F*% = 5.00%), it was better than panobinostat (*F*% = 4.62%) and comparable to SAHA (*F*% = 8.33%) [46,47]. This preliminary pharmacokinetic assessment highlights the need to refine its pharmacokinetic parameters as a crucial step in further development and optimization, aiming to enhance in vivo stability, extend the duration of action, and elevate oral bioavailability.

## 3. Materials and Methods

### 3.1. Chemistry

All solvents used were commercially available and of analytical grade unless stated otherwise. Chemical shifts are given in ppm with the solvent as internal standard or TMS, if added. Protons on heteroatoms such as COOH, NH, and OH protons are only reported when detected in NMR and may therefore be missing. NMR spectra were acquired on Varian Mercury 400, Bruker AM-400, and AM-500 spectrometers using CDCl_3_ or CD_3_OD as solvent (Bruker, Billerica, MA, USA). Unless otherwise noted, all reagents were commercially available (Sinopharm (Beijing, China), Energy, Bide, Adamas-beta (Shanghai, China), TCI (Qingdao, China), J&K (Beijing, China), etc.) and used without further purification. Purifications were performed by flash column chromatography using a Biotage Isolera (Shanghai, China) one-flash purification system. The chromatographic columns used with the Biotage Isolera system were purchased from Changzhou Santai Technology Co., Ltd. (Changzhou, China).

#### 3.1.1. Methyl 4-(2-acetyl-1*H*-pyrrol-1-yl)butanoate (**2a**)

To a solution of 2-acetyl pyrrole (109 mg, 1.00 mmol) in dry DMF (10 mL) was added NaH (44 mg, 60%, dispersion in paraffin liquid, 1.10 mmol) at 0 °C. The resulting mixture was stirred for 15 min followed by the addition of methyl 4-bromobutyrate (**1a**) (217 mg, 1.20 mmol). Then, the mixture was stirred at room temperature and monitored by TLC. After 12 h, H_2_O (20 mL) was added, and the mixture was extracted with ethyl acetate (3 × 10 mL). The combined organic layers were washed with brine, dried by anhydrous Na_2_SO_4_, filtered, and then concentrated under reduced pressure to give the crude product, which was further purified by column chromatography eluting with 10% ethyl acetate in petroleum ether to give **2a** as a colorless oil (128 mg, 61%). ^1^H NMR (400 MHz, CDCl_3_) δ 6.93 (dd, *J* = 4.1, 1.7 Hz, 1H), 6.82 (t, *J* = 2.1 Hz, 1H), 6.10 (dd, *J* = 4.1, 2.5 Hz, 1H), 4.34 (t, *J* = 6.9 Hz, 2H), 3.63 (s, 3H), 2.39 (s, 3H), 2.26 (t, *J* = 7.3 Hz, 2H), 2.03 (p, *J* = 7.1 Hz, 2H). ^13^C NMR (100 MHz, CDCl_3_) δ 188.3, 173.4, 130.4, 130.1, 120.5, 108.2, 51.7, 48.6, 30.7, 27.3, 26.5.

#### 3.1.2. Ethyl 5-(2-acetyl-1*H*-pyrrol-1-yl)pentanoate (**2b**)

Starting from ethyl 5-bromovalerate (**1b**) (500 mg, 2.39 mmol) and 2-acetyl pyrrole (217 mg, 1.99 mmol), the title compound was obtained following the procedure previously described for compound **2a**. The crude product was purified by silica gel chromatography to give the colorless oil **2b** (388 mg, 82%). ^1^H NMR (400 MHz, CDCl_3_) δ 6.95 (dd, *J* = 4.1, 1.7 Hz, 1H), 6.84 (t, *J* = 2.2 Hz, 1H), 6.11 (dd, *J* = 4.1, 2.5 Hz, 1H), 4.31 (t, *J* = 7.1 Hz, 2H), 4.10 (q, *J* = 7.1 Hz, 2H), 2.42 (s, 3H), 2.30 (t, *J* = 7.4 Hz, 2H), 1.79–1.71 (m, 2H), 1.62–1.55 (m, 2H), 1.23 (t, *J* = 7.1 Hz, 3H). ^13^C NMR (100 MHz, CDCl_3_) δ 188.4, 173.5, 130.3, 130.1, 120.5, 108.1, 60.4, 49.5, 33.9, 30.9, 27.4, 22.1, 14.3.

#### 3.1.3. Ethyl 6-(2-acetyl-1*H*-pyrrol-1-yl)hexanoate (**2c**)

Starting from ethyl 6-bromohexanoate (**1c**) (533 mg, 2.39 mmol) and 2-acetyl pyrrole (217 mg, 1.99 mmol), the title compound was obtained following the procedure previously described for compound **2a**. The crude product was purified by silica gel chromatography to give the colorless oil **2c** (386 mg, 77%). ^1^H NMR (400 MHz, CDCl_3_) δ 6.95 (dd, *J* = 4.0, 1.7 Hz, 1H), 6.84 (dd, *J* = 2.5, 1.8 Hz, 1H), 6.11 (dd, *J* = 4.1, 2.5 Hz, 1H), 4.29 (t, *J* = 7.2 Hz, 2H), 4.10 (q, *J* = 7.1 Hz, 2H), 2.42 (s, 3H), 2.28 (t, *J* = 7.5 Hz, 2H), 1.78–1.70 (m, 2H), 1.67–1.58 (m, 2H), 1.34–1.27 (m, 2H), 1.24 (t, *J* = 7.1 Hz, 3H). ^13^C NMR (100 MHz, CDCl_3_) δ 188.4, 173.8, 130.3, 130.1, 120.4, 108.0, 60.4, 49.7, 34.3, 31.2, 27.5, 26.2, 24.7, 14.4.

#### 3.1.4. Ethyl 7-(2-acetyl-1*H*-pyrrol-1-yl)heptanoate (**2d**)

Starting from ethyl 7-bromoheptanoate (**1d**) (566 mg, 2.39 mmol) and 2-acetyl pyrrole (217 mg, 1.99 mmol), the title compound was obtained following the procedure previously described for compound **2a**. The crude product was purified by silica gel chromatography to give the colorless oil **2d** (498 mg, 94%). ^1^H NMR (400 MHz, CDCl_3_) δ 6.94 (dd, *J* = 4.1, 1.7 Hz, 1H), 6.83 (t, *J* = 2.1 Hz, 1H), 6.10 (dd, *J* = 4.0, 2.5 Hz, 1H), 4.28 (t, *J* = 7.4 Hz, 2H), 4.10 (q, *J* = 7.1 Hz, 2H), 2.41 (s, 3H), 2.26 (t, *J* = 7.5 Hz, 2H), 1.74–1.66 (m, 2H), 1.63–1.53 (m, 2H), 1.36–1.26 (overlap, 4H), 1.23 (t, *J* = 7.1 Hz, 3H). ^13^C NMR (100 MHz, CDCl_3_) δ 188.3, 173.9, 130.3, 130.1, 120.4, 108.0, 60.3, 49.8, 34.3, 31.4, 28.8, 27.5, 26.4, 24.9, 14.4.

#### 3.1.5. Ethyl 8-(2-acetyl-1*H*-pyrrol-1-yl)octanoate (**2e**)

Starting from ethyl 8-bromooctanoate (**1e**) (379 mg, 1.51 mmol) and 2-acetyl pyrrole (137 mg, 1.26 mmol), the title compound was obtained following the procedure previously described for compound **2a**. The crude product was purified by silica gel chromatography to give the colorless oil **2e** (300 mg, 85%). ^1^H NMR (400 MHz, CDCl_3_) δ 6.95 (dd, *J* = 4.1, 1.8 Hz, 1H), 6.83 (t, *J* = 2.1 Hz, 1H), 6.11 (dd, *J* = 4.0, 2.5 Hz, 1H), 4.28 (t, *J* = 7.3 Hz, 2H), 4.11 (q, *J* = 7.1 Hz, 2H), 2.42 (s, 3H), 2.26 (t, *J* = 7.5 Hz, 2H), 1.72–1.67 (m, 2H), 1.62–1.55 (m, 2H), 1.32–1.26 (overlap, 6H), 1.24 (t, *J* = 7.1 Hz, 3H). ^13^C NMR (125 MHz, CDCl_3_) δ 188.3, 174.0, 130.3, 130.2, 120.4, 108.0, 60.3, 49.9, 34.5, 31.5, 29.2, 29.0, 27.5, 26.6, 25.0, 14.4.

#### 3.1.6. 4-(2-Acetyl-1*H*-pyrrol-1-yl)-*N*-hydroxybutanamide (**3a**)

Potassium hydroxide (134 mg, 2.39 mmol) was added to a solution of hydroxylamine hydrochloride (147 mg, 2.13 mmol) in MeOH (15 mL) at 0 °C. The mixture was stirred for 30 min at 0 °C. Then compound **2a** (128 mg, 0.61 mmol) was added, and the mixture was stirred at 40 °C overnight. The reaction was complete detected by TLC. After cooling to room temperature, the solvent was removed under reduced pressure to give a yellow oil. H_2_O (15 mL) was added, and the mixture was adjusted to pH 7 with acetic acid and extracted with ethyl acetate (3 × 10 mL). The combined organic layers were washed with brine, dried by anhydrous Na_2_SO_4_, filtered, and concentrated under reduced pressure to give a crude product, which was purified by flash silica gel column (1%–10% MeOH/DCM) to afford compound **3a** as a light brown solid (86 mg, 67%). ^1^H NMR (400 MHz, CDCl_3_) δ 6.91–6.84 (overlap, 2H), 6.03 (t, *J* = 3.3 Hz, 1H), 4.20 (t, *J* = 7.2 Hz, 2H), 2.32 (s, 3H), 2.12 (t, *J* = 7.4 Hz, 2H), 1.99–1.89 (m, 2H). ^13^C NMR (100 MHz, CDCl_3_) δ 188.9, 171.0, 131.4, 129.7, 121.4, 108.5, 48.8, 29.7, 27.2, 27.1. HRMS (ESI), *m*/*z*: calcd for C_10_H_13_N_2_O_3_^−^: 209.0932 [M − H]^−^; found: 209.0931.

#### 3.1.7. 5-(2-Acetyl-1*H*-pyrrol-1-yl)-*N*-hydroxypentanamide (**3b**)

Compound **3b** was prepared using similar procedures as for **3a** from compound **2b** (388 mg, 1.64 mmol). The crude product was purified by silica gel chromatography to give the light brown solid (106 mg, 29%). ^1^H NMR (400 MHz, CDCl_3_) δ 6.94 (dd, *J* = 4.1, 1.5 Hz, 1H), 6.86 (d, *J* = 2.0 Hz, 1H), 6.08 (dd, *J* = 4.1, 2.4 Hz, 1H), 4.20 (t, *J* = 7.1 Hz, 2H), 2.38 (s, 3H), 2.16 (t, *J* = 7.2 Hz, 2H), 1.72–1.61 (m, 2H), 1.60–1.51 (m, 2H). ^13^C NMR (100 MHz, CDCl_3_) δ 189.1, 171.7, 131.2, 129.8, 121.4, 108.5, 49.2, 32.1, 30.7, 27.3, 22.4. HRMS (ESI), *m*/*z*: calcd for C_11_H_15_N_2_O_3_^−^: 223.1088 [M − H]^−^; found: 223.1085.

#### 3.1.8. 6-(2-Acetyl-1*H*-pyrrol-1-yl)-*N*-hydroxyhexanamide (**3c**)

Compound **3c** was prepared using similar procedures as for **3a** from compound **2c** (386 mg, 1.54 mmol). The crude product was purified by silica gel chromatography to give the light brown solid (154 mg, 42%). ^1^H NMR (400 MHz, CDCl_3_) δ 6.92 (dd, *J* = 4.2, 1.7 Hz, 1H), 6.83 (t, *J* = 2.1 Hz, 1H), 6.06 (dd, *J* = 4.1, 2.5 Hz, 1H), 4.19 (t, *J* = 7.4 Hz, 2H), 2.37 (s, 3H), 2.13 (t, *J* = 7.5 Hz, 2H), 1.69–1.55 (overlap, 4H), 1.32–1.22 (m, 2H). ^13^C NMR (100 MHz, CDCl_3_) δ 188.7, 171.7, 130.8, 129.7, 121.0, 108.2, 49.5, 32.5, 30.9, 27.2, 25.8, 24.8. HRMS (ESI), *m*/*z*: calcd for C_12_H_19_N_2_O_3_^+^: 239.1390 [M + H]^+^; found: 239.1389.

#### 3.1.9. 7-(2-Acetyl-1*H*-pyrrol-1-yl)-*N*-hydroxyheptanamide (**3d**)

Compound **3d** was prepared using similar procedures as for **3a** from compound **2d** (498 mg, 1.88 mmol). The crude product was purified by silica gel chromatography to give the light brown solid (166 mg, 35%). ^1^H NMR (400 MHz, CDCl_3_) δ 6.94 (dd, *J* = 4.1, 1.7 Hz, 1H), 6.83 (t, *J* = 2.1 Hz, 1H), 6.08 (dd, *J* = 4.1, 2.5 Hz, 1H), 4.21 (t, *J* = 7.4 Hz, 2H), 2.40 (s, 3H), 2.12 (t, *J* = 7.4 Hz, 2H), 1.69–1.60 (m, 2H), 1.59–1.50 (m, 2H), 1.32–1.20 (overlap, 4H). ^13^C NMR (100 MHz, CDCl_3_) δ 188.7, 171.8, 130.7, 129.8, 121.0, 108.2, 49.7, 32.7, 31.1, 28.4, 27.3, 26.0, 25.2. HRMS (ESI), *m*/*z*: calcd for C_13_H_19_N_2_O_3_^−^: 251.1401 [M − H]^−^; found: 251.1399.

#### 3.1.10. 8-(2-Acetyl-1*H*-pyrrol-1-yl)-*N*-hydroxyoctanamide (**3e**)

Compound **3e** was prepared using similar procedures as for **3a** from compound **2e** (300 mg, 1.08 mmol). The crude product was purified by silica gel chromatography to give the light brown solid (121 mg, 42%). ^1^H NMR (400 MHz, CDCl_3_) δ 6.98 (dd, *J* = 4.1, 1.7 Hz, 1H), 6.86 (t, *J* = 2.1 Hz, 1H), 6.13 (dd, *J* = 4.1, 2.5 Hz, 1H), 4.27 (t, *J* = 7.3 Hz, 2H), 2.44 (s, 3H), 2.16 (t, *J* = 7.3 Hz, 2H), 1.72–1.60 (overlap, 4H), 1.33–1.26 (overlap, 6H). ^13^C NMR (125 MHz, CDCl_3_) δ 188.9, 171.5, 130.7, 130.1, 120.9, 108.3, 49.8, 32.9, 31.3, 28.6, 28.4, 27.4, 26.2, 25.2. HRMS (ESI), *m*/*z*: calcd for C_14_H_21_N_2_O_3_^−^: 265.1558 [M − H]^−^; found: 265.1558.

#### 3.1.11. Ethyl 7-(1*H*-pyrrol-1-yl)heptanoate (**4a**)

Starting from ethyl 7-bromoheptanoate (**1d**) (1.55 g, 6.53 mmol) and pyrrole (364 mg, 5.44 mmol), the title compound was obtained following the procedure previously described for compound **2a**. The crude product was purified by silica gel chromatography to give the colorless oil **4a** (546 mg, 45%). ^1^H NMR (400 MHz, CDCl_3_) δ 6.63 (t, *J* = 2.1 Hz, 2H), 6.12 (t, *J* = 2.1 Hz, 2H), 4.12 (q, *J* = 7.1 Hz, 2H), 3.86 (t, *J* = 7.1 Hz, 2H), 2.27 (t, *J* = 7.5 Hz, 2H), 1.76 (p, *J* = 7.3 Hz, 2H), 1.64–1.57 (m, 2H), 1.37–1.28 (overlap, 4H), 1.25 (t, *J* = 7.1 Hz, 3H). ^13^C NMR (100 MHz, CDCl_3_) δ 173.8, 120.5 × 2, 107.9 × 2, 60.3, 49.6, 34.3, 31.5, 28.8, 26.5, 24.9, 14.4.

#### 3.1.12. Ethyl 7-(2-propionyl-1*H*-pyrrol-1-yl)heptanoate (**4b**)

Starting from ethyl 7-bromoheptanoate (**1d**) (927 mg, 3.91 mmol) and 2-propionylpyrrole (401 mg, 3.26 mmol), the title compound was obtained following the procedure previously described for compound **2a**. The crude product was purified by silica gel chromatography to give the colorless oil **4b** (791 mg, 87%). ^1^H NMR (600 MHz, CDCl_3_) δ 6.96 (dd, *J* = 4.1, 1.7 Hz, 1H), 6.83 (t, *J* = 2.1 Hz, 1H), 6.10 (dd, *J* = 4.1, 2.5 Hz, 1H), 4.29 (t, *J* = 7.3 Hz, 2H), 4.11 (q, *J* = 7.1 Hz, 2H), 2.81 (q, *J* = 7.4 Hz, 2H), 2.26 (t, *J* = 7.5 Hz, 2H), 1.72 (p, *J* = 7.4 Hz, 2H), 1.60 (p, *J* = 7.5 Hz, 2H), 1.36–1.27 (overlap, 4H), 1.24 (t, *J* = 7.1 Hz, 3H), 1.17 (t, *J* = 7.4 Hz, 3H). ^13^C NMR (150 MHz, CDCl_3_) δ 191.8, 173.9, 130.0, 129.8, 119.3, 107.9, 60.3, 49.9, 34.4, 32.4, 31.5, 28.9, 26.4, 25.0, 14.4, 9.1.

#### 3.1.13. Ethyl 7-(2-(2,2,2-trifluoroacetyl)-1*H*-pyrrol-1-yl)heptanoate (**4c**)

Starting from ethyl 7-bromoheptanoate (**1d**) (870 mg, 3.67 mmol) and 2-(trifluoroacetyl)pyrrole (499 mg, 3.06 mmol), the title compound was obtained following the procedure previously described for compound **2a**. The crude product was purified by silica gel chromatography to give the colorless oil **4c** (810 mg, 83%). ^1^H NMR (600 MHz, CDCl_3_) δ 7.24–7.21 (m, 1H), 7.08 (t, *J* = 2.0 Hz, 1H), 6.26 (dd, *J* = 4.4, 2.4 Hz, 1H), 4.31 (t, *J* = 7.3 Hz, 2H), 4.12 (q, *J* = 7.1 Hz, 2H), 2.28 (t, *J* = 7.5 Hz, 2H), 1.77–1.71 (m, 2H), 1.62–1.60 (m, 2H), 1.38–1.29 (overlap, 4H), 1.25 (t, *J* = 7.1 Hz, 3H). ^13^C NMR (150 MHz, CDCl_3_) δ 173.8, (169.9, 169.7), 134.2, (124.6, 124.5), 124.1, (118.2, 116.3), 110.3, 60.4, 50.3, 34.3, 31.1, 28.7, 26.3, 24.9, 14.4.

#### 3.1.14. Ethyl 7-(2-(2,2,2-trichloroacetyl)-1*H*-pyrrol-1-yl)heptanoate (**4d**)

Starting from ethyl 7-bromoheptanoate (**1d**) (668 mg, 2.82 mmol) and 2-(trichloroacetyl)pyrrole (498 mg, 2.35 mmol), the title compound was obtained following the procedure previously described for compound **2a**. The crude product was purified by silica gel chromatography to give the colorless oil **4d** (675 mg, 78%). ^1^H NMR (600 MHz, CDCl_3_) δ 7.52 (dd, *J* = 4.4, 1.6 Hz, 1H), 7.00 (t, *J* = 2.0 Hz, 1H), 6.21 (dd, *J* = 4.4, 2.4 Hz, 1H), 4.29 (t, *J* = 7.3 Hz, 2H 2H), 4.11 (q, *J* = 7.2 Hz, 2H), 2.27 (t, *J* = 7.5 Hz, 2H), 1.75 (p, *J* = 7.2 Hz, 2H), 1.61 (p, *J* = 7.4 Hz, 2H), 1.37–1.30 (overlap, 4H), 1.24 (t, *J* = 7.2 Hz, 3H). ^13^C NMR (150 MHz, CDCl_3_) δ 173.8, 172.6, 133.1, 124.7, 121.1, 109.1, 96.6, 60.3, 50.7, 34.3, 31.1, 28.8, 26.3, 24.9, 14.4.

#### 3.1.15. Ethyl 7-(2-benzoyl-1*H*-pyrrol-1-yl)heptanoate (**4e**)

Starting from ethyl 7-bromoheptanoate (**1d**) (827 mg, 3.49 mmol) and 2-benzoylpyrrole (498 mg, 2.91 mmol), the title compound was obtained following the procedure previously described for compound **2a**. The crude product was purified by silica gel chromatography to give the colorless oil **4e** (752 mg, 79%). ^1^H NMR (400 MHz, CDCl_3_) δ 7.79–7.75 (overlap, 2H), 7.53–749 (m, 1H), 7.45–7.40 (overlap, 2H), 6.96 (dd, *J* = 2.5, 1.7 Hz, 1H), 6.72 (dd, *J* = 4.0, 1.7 Hz, 1H), 6.14 (dd, *J* = 4.1, 2.5 Hz, 1H), 4.39 (t, *J* = 7.4 Hz, 2H), 4.11 (q, *J* = 7.1 Hz, 2H), 2.27 (t, *J* = 7.5 Hz, 2H), 1.86–1.76 (m, 2H), 1.67–1.56 (m, 2H), 1.38–1.30 (overlap, 4H), 1.24 (t, *J* = 7.1 Hz, 3H). ^13^C NMR (100 MHz, CDCl_3_) δ 186.2, 173.8, 140.3, 131.4, 130.7, 129.9, 129.3 × 2, 128.1 × 2, 123.6, 108.2, 60.3, 49.6, 34.3, 31.7, 28.8, 26.5, 25.0, 14.4.

#### 3.1.16. Ethyl 7-(1*H*-indol-1-yl)heptanoate (**4f**)

Starting from ethyl 7-bromoheptanoate (**1d**) (1.21 g, 5.10 mmol) and indole (497 mg, 4.25 mmol), the title compound was obtained following the procedure previously described for compound **2a**. The crude product was purified by silica gel chromatography to give the colorless oil **4f** (963 mg, 83%). ^1^H NMR (600 MHz, CDCl_3_) δ 7.62 (d, *J* = 7.9 Hz, 1H), 7.32 (d, *J* = 8.3 Hz, 1H), 7.19 (t, *J* = 7.6 Hz, 1H), 7.11–7.04 (overlap, 2H), 6.47(d, *J* = 3.1 Hz, 1H), 4.13–4.06 (overlap, 4H), 2.25 (t, *J* = 7.5 Hz, 2H), 1.82 (p, *J* = 7.1 Hz, 2H), 1.59 (p, *J* = 7.5 Hz, 2H), 1.35–1.28 (overlap, 4H), 1.23 (t, *J* = 7.2 Hz, 3H). ^13^C NMR (150 MHz, CDCl_3_) δ 173.7, 136.0, 128.7, 127.8, 121.4, 121.0, 119.3, 109.4, 101.0, 60.3, 46.3, 34.3, 30.1, 28.8, 26.7, 24.9, 14.3.

#### 3.1.17. Ethyl 7-(5-methyl-1*H*-indol-1-yl)heptanoate (**4g**)

Starting from ethyl 7-bromoheptanoate (**1d**) (870 mg, 3.67 mmol) and 5-methylindole (401 mg, 3.06 mmol), the title compound was obtained following the procedure previously described for compound **2a**. The crude product was purified by silica gel chromatography to give the colorless oil **4g** (677 mg, 77%). ^1^H NMR (600 MHz, CDCl_3_) δ 7.42–7.39 (m, 1H), 7.22 (d, *J* = 8.3 Hz, 1H), 7.05–7.01 (overlap, 2H), 6.39 (dd, *J* = 3.1, 0.9 Hz, 1H), 4.13–4.09 (m, 2H), 4.08 (t, *J* = 7.1 Hz, 2H), 2.45 (s, 3H), 2.26 (t, *J* = 7.5 Hz, 2H), 1.83 (p, *J* = 7.1 Hz, 2H), 1.62–1.57 (m, 2H), 1.40–1.29 (overlap, 4H), 1.25 (t, *J* = 7.1 Hz, 3H). ^13^C NMR (150 MHz, CDCl_3_) δ 173.8, 134.5, 129.0, 128.5, 128.0, 123.1, 120.7, 109.2, 100.4, 60.4, 46.5, 34.4, 30.2, 28.9, 26.8, 24.9, 21.5, 14.4.

#### 3.1.18. Ethyl 7-(6-fluoro-1*H*-indol-1-yl)heptanoate (**4h**)

Starting from ethyl 7-bromoheptanoate (**1d**) (1.39 g, 5.87 mmol) and 6-fluoroindole (660 mg, 4.89 mmol), the title compound was obtained following the procedure previously described for compound **2a**. The crude product was purified by silica gel chromatography to give the colorless oil **4h** (711 mg, 50%). ^1^H NMR (400 MHz, CDCl_3_) δ 7.52 (dd, *J* = 8.6, 5.4 Hz, 1H), 7.06 (d, *J* = 3.2 Hz, 1H), 7.00 (dd, *J* = 10.0, 2.3 Hz, 1H), 6.86 (ddd, *J* = 9.6, 8.6, 2.3 Hz, 1H), 6.46 (dd, *J* = 3.1, 0.9 Hz, 1H), 4.12 (q, *J* = 7.1 Hz, 2H), 4.04 (t, *J* = 7.1 Hz, 2H), 2.27 (t, *J* = 7.5 Hz, 2H), 1.82 (p, *J* = 7.3 Hz, 2H), 1.64–1.58 (m, 2H), 1.40–1.29 (overlap, 4H), 1.25 (t, *J* = 7.1 Hz, 3H). ^13^C NMR (100 MHz, CDCl_3_) δ 173.8, (161.0 158.6), (136.1, 136.0), (128.3, 128.2), 125.1, (121.8, 121.7), (108.2, 107.9), 101.3, (96.0, 95.7), 60.4, 46.5, 34.3, 30.0, 28.8, 26.7, 24.9, 14.4.

#### 3.1.19. Ethyl 7-(5-fluoro-1*H*-indol-1-yl)heptanoate (**4i**)

Starting from ethyl 7-bromoheptanoate (**1d**) (1.05 g, 4.44 mmol) and 5-fluoroindole (500 mg, 3.70 mmol), the title compound was obtained following the procedure previously described for compound **2a**. The crude product was purified by silica gel chromatography to give the colorless oil **4i** (829 mg, 77%). ^1^H NMR (400 MHz, CDCl_3_) δ 7.27–7.23 (m, 1H), 7.22–7.19 (m, 1H), 7.10 (d, *J* = 3.1 Hz, 1H), 6.93 (td, *J* = 9.1, 2.5 Hz, 1H), 6.42 (dd, *J* = 3.1, 0.8 Hz, 1H), 4.13–4.04 (overlap, 4H), 2.26 (t, *J* = 7.4 Hz, 2H), 1.81 (p, *J* = 7.2 Hz, 2H), 1.63–1.55 (m, 2H), 1.37–1.27 (overlap, 4H), 1.24 (t, *J* = 7.1 Hz, 3H). ^13^C NMR (100 MHz, CDCl_3_) δ 173.7, (159.0, 156.7), 132.7, 129.4, (128.9, 128.8), (110.0, 109.7), (109.96, 109.94), (105.8, 105.5), (101.0, 100.9), 60.3, 46.6, 34.3, 30.1, 28.8, 26.7, 24.9, 14.4.

#### 3.1.20. Ethyl 7-(5-chloro-1*H*-indol-1-yl)heptanoate (**4j**)

Starting from ethyl 7-bromoheptanoate (**1d**) (754 mg, 3.18 mmol) and 5-chloroindole (400 mg, 2.65 mmol), the title compound was obtained following the procedure previously described for compound **2a**. The crude product was purified by silica gel chromatography to give the colorless oil **4j** (724 mg, 89%). ^1^H NMR (600 MHz, CDCl_3_) δ 7.58 (d, *J* = 2.0 Hz, 1H), 7.24 (d, *J* = 8.7 Hz, 1H), 7.14 (dd, *J* = 8.7, 2.0 Hz, 1H), 7.10 (d, *J* = 3.1 Hz, 1H), 6.42 (d, *J* = 3.0 Hz, 1H), 4.19–4.02 (overlap, 4H), 2.26 (t, *J* = 7.5 Hz, 2H), 1.82 (p, *J* = 7.2 Hz, 2H), 1.62–1.57 (m, 2H), 1.36–1.30 (overlap, 4H), 1.24 (t, *J* = 7.1 Hz, 3H). ^13^C NMR (150 MHz, CDCl_3_) δ 173.8, 134.5, 129.7, 129.2, 125.1, 121.8, 120.4, 110.5, 100.8, 60.4, 46.6, 34.3, 30.2 28.8, 26.7, 24.9, 14.4.

#### 3.1.21. Ethyl 7-(5-bromo-1*H*-indol-1-yl)heptanoate (**4k**)

Starting from ethyl 7-bromoheptanoate (**1d**) (723 mg, 3.05 mmol) and 5-bromoindole (498 mg, 2.54 mmol), the title compound was obtained following the procedure previously described for compound **2a**. The crude product was purified by silica gel chromatography to give the colorless oil **4k** (715 mg, 80%). ^1^H NMR (600 MHz, CDCl_3_) δ 7.73 (d, *J* = 1.9 Hz, 1H), 7.26 (dd, *J* = 8.8, 1.8 Hz, 1H), 7.18 (d, *J* = 8.7 Hz, 1H), 7.07 (d, *J* = 3.1 Hz, 1H), 6.41 (d, *J* = 3.1 Hz, 1H), 4.11 (q, *J* = 7.1 Hz, 2H), 4.06 (t, *J* = 7.1 Hz, 2H), 2.25 (t, *J* = 7.5 Hz, 2H), 1.80 (p, *J* = 7.2 Hz, 2H), 1.59 (p, *J* = 7.5 Hz, 2H), 1.35–1.27 (overlap, 4H), 1.24 (t, *J* = 7.2 Hz, 3H). ^13^C NMR (150 MHz, CDCl_3_) δ 173.7, 134.7, 130.3, 129.0, 124.3, 123.5, 112.6, 110.9, 100.7, 60.4, 46.6, 34.3, 30.1, 28.8, 26.7, 24.8, 14.4.

#### 3.1.22. Ethyl 7-(5-cyano-1*H*-indol-1-yl)heptanoate (**4l**)

Starting from ethyl 7-bromoheptanoate (**1d**) (1.41 g, 5.94 mmol) and 5-cyanoindole (703 mg, 4.95 mmol), the title compound was obtained following the procedure previously described for compound **2a**. The crude product was purified by silica gel chromatography to give the colorless oil **4l** (1.33 g, 90%). ^1^H NMR (400 MHz, CDCl_3_) δ 7.95–7.92 (m, 1H), 7.45–7.30 (overlap, 2H), 7.20 (d, *J* = 3.2 Hz, 1H), 6.55 (dd, *J* = 3.2, 0.8 Hz, 1H), 4.15–4.06 (overlap, 4H), 2.25 (t, *J* = 7.4 Hz, 2H), 1.83 (p, *J* = 7.2 Hz, 2H), 1.59 (p, *J* = 7.5 Hz, 2H), 1.37–1.27 (overlap, 4H), 1.23 (t, *J* = 7.1 Hz, 3H). ^13^C NMR (100 MHz, CDCl_3_) δ 173.6, 137.5, 130.2, 128.3, 126.6, 124.4, 120.9, 110.3, 102.3 × 2, 60.3, 46.6, 34.2, 30.1, 28.7, 26.6, 24.7, 14.3.

#### 3.1.23. *N*-hydroxy-7-(1*H*-pyrrol-1-yl)heptanamide (**5a**)

Compound **5a** was prepared using similar procedures as for **3a** from compound **4a** (546 mg, 2.45 mmol). The crude product was purified by silica gel chromatography to give the dark brown solid (360 mg, 70%). ^1^H NMR (600 MHz, CDCl_3_) δ 6.65 (t, *J* = 2.1 Hz, 2H), 6.15 (t, *J* = 2.1 Hz, 2H), 3.86 (t, *J* = 7.1 Hz, 2H), 2.09 (t, *J* = 7.5 Hz, 2H), 1.74 (p, *J* = 7.1 Hz, 2H), 1.59 (p, *J* = 7.5 Hz, 2H), 1.34–1.24 (overlap, 4H). ^13^C NMR (150 MHz, CDCl_3_) δ 172.0, 120.6 × 2, 107.9 × 2, 49.5, 32.7, 31.3, 28.6, 26.3, 25.2. HRMS (ESI), *m*/*z*: calcd for C_11_H_17_N_2_O_2_^−^: 209.1296 [M − H]^−^; found: 209.1298.

#### 3.1.24. *N*-hydroxy-7-(2-propionyl-1*H*-pyrrol-1-yl)heptanamide (**5b**)

Compound **5b** was prepared using similar procedures as for **3a** from compound **4b** (399 mg, 1.43 mmol). The crude product was purified by silica gel chromatography to give the white solid (228 mg, 60%). ^1^H NMR (600 MHz, CDCl_3_) δ 6.97 (dd, *J* = 4.1, 1.7 Hz, 1H), 6.84 (t, *J* = 2.1 Hz, 1H), 6.10 (dd, *J* = 4.1, 2.4 Hz, 1H), 4.25 (t, *J* = 7.5 Hz, 2H), 2.81 (q, *J* = 7.4 Hz, 2H), 2.15 (t, *J* = 7.5 Hz, 2H), 1.69 (p, *J* = 7.4 Hz, 2H), 1.61 (p, *J* = 7.4 Hz, 2H), 1.36–1.26 (overlap, 4H), 1.16 (t, *J* = 7.4 Hz, 3H). ^13^C NMR (150 MHz, CDCl_3_) δ 192.4, 171.7, 130.4, 129.7, 119.9, 108.1, 49.8, 32.8, 32.4, 31.1, 28.3, 26.0, 25.1, 9.3. HRMS (ESI), *m*/*z*: calcd for C_14_H_21_N_2_O_3_^−^: 265.1558 [M − H]^−^; found: 265.1557.

#### 3.1.25. *N*-hydroxy-7-(2-(2,2,2-trifluoroacetyl)-1*H*-pyrrol-1-yl)heptanamide (**5c**)

Compound **5c** was prepared using similar procedures as for **3a** from compound **4c** (393 mg, 1.23 mmol). The crude product was purified by silica gel chromatography to give the light brown solid (53 mg, 14%). ^1^H NMR (600 MHz, CDCl_3_ (CD_3_OD)) δ 7.22–7.18 (m, 1H), 7.09–7.06 (m, 1H), 6.24 (t, *J* = 3.4 Hz, 1H), 4.26 (t, *J* = 7.4 Hz, 2H), 2.08 (t, *J* = 7.5 Hz, 2H), 1.68 (p, *J* = 7.4 Hz, 2H), 1.57 (p, *J* = 7.4 Hz, 2H), 1.32–1.25 (overlap, 4H). ^13^C NMR (151 MHz, CDCl_3_ (CD_3_OD)) δ 171.6, (169.8, 169.6), 134.6, (124.86, 124.84, 124.81), 123.9, (118.1, 116.2), 110.4, 50.2, 32.7, 30.9, 28.5, 26.0, 25.2. HRMS (ESI), *m*/*z*: calcd for C_13_H_16_F_3_N_2_O_3_^−^: 305.1119 [M − H]^−^; found: 305.1119.

#### 3.1.26. Methyl 1-(7-(hydroxyamino)-7-oxoheptyl)-1*H*-pyrrole-2-carboxylate (**5d**)

Compound **5d** was prepared using similar procedures as for **3a** from compound **4d** (412 mg, 1.12 mmol). The crude product was purified by silica gel chromatography to give the black solid (48 mg, 16%). ^1^H NMR (600 MHz, CDCl_3_) δ 6.93 (dd, *J* = 3.9, 1.8 Hz, 1H), 6.82 (t, *J* = 2.2 Hz, 1H), 6.09 (dd, *J* = 4.0, 2.5 Hz, 1H), 4.24 (t, *J* = 7.4 Hz, 2H), 3.78 (s, 3H), 2.11 (t, *J* = 7.6 Hz, 2H), 1.72 (p, *J* = 7.4 Hz, 2H), 1.59 (p, *J* = 7.4 Hz, 2H), 1.32–1.24 (overlap, 4H). ^13^C NMR (150 MHz, CDCl_3_) δ 171.8, 161.8, 129.0, 121.4, 118.5, 108.1, 51.2, 49.1, 32.8, 31.3, 28.4, 26.1, 25.2. HRMS (ESI), *m*/*z*: calcd for C_13_H_19_N_2_O_4_^−^: 267.1350 [M − H]^−^; found: 267.1350.

#### 3.1.27. 7-(2-Benzoyl-1*H*-pyrrol-1-yl)-*N*-hydroxyheptanamide (**5e**)

Compound **5e** was prepared using similar procedures as for **3a** from compound **4e** (396 mg, 1.21 mmol). The crude product was purified by silica gel chromatography to give the gray solid (114 mg, 30%). ^1^H NMR (600 MHz, CDCl_3_) δ 7.77–7.70 (overlap, 2H), 7.50 (t, *J* = 7.5 Hz, 1H), 7.43–7.38 (overlap, 2H), 6.96–6.93 (m, 1H), 6.71 (d, *J* = 4.1 Hz, 1H), 6.13 (t, *J* = 3.2 Hz, 1H), 4.32 (t, *J* = 7.5 Hz, 2H), 2.09 (t, *J* = 7.5 Hz, 2H), 1.76 (p, *J* = 7.1 Hz, 2H), 1.57 (t, *J* = 7.3 Hz, 2H), 1.33–1.25 (overlap, 4H). ^13^C NMR (150 MHz, CDCl_3_) δ 186.5, 171.8, 140.1, 131.5, 131.2, 129.7, 129.2 × 2, 128.1 × 2, 124.1, 108.4, 49.5, 32.7, 31.4, 28.4, 26.1, 25.2. HRMS (ESI), *m*/*z*: calcd for C_18_H_21_N_2_O_3_^−^: 313.1558 [M − H]^−^; found: 313.1557.

#### 3.1.28. *N*-hydroxy-7-(1*H*-indol-1-yl)heptanamide (**5f**)

Compound **5f** was prepared using similar procedures as for **3a** from compound **4f** (399 mg, 1.46 mmol). The crude product was purified by silica gel chromatography to give the brown solid (205 mg, 54%). ^1^H NMR (600 MHz, CDCl_3_) δ 7.62 (d, *J* = 7.9 Hz, 1H), 7.32 (d, *J* = 8.2 Hz, 1H), 7.20 (t, *J* = 7.6 Hz, 1H), 7.09 (t, *J* = 7.5 Hz, 1H), 7.06 (d, *J* = 3.1 Hz, 1H), 6.47 (d, *J* = 3.1 Hz, 1H), 4.09 (t, *J* = 7.0 Hz, 2H), 1.99 (t, *J* = 7.5 Hz, 2H), 1.80 (p, *J* = 7.1 Hz, 2H), 1.55 (q, *J* = 7.4 Hz, 2H), 1.30–1.23 (overlap, 4H). ^13^C NMR (150 MHz, CDCl_3_) δ 171.7, 136.1, 128.7, 128.0, 121.5, 121.1, 119.4, 109.5, 101.1, 46.4, 32.7, 30.0, 28.6, 26.6, 25.1. HRMS (ESI), *m*/*z*: calcd for C_15_H_19_N_2_O_2_^−^: 259.1452 [M − H]^−^; found: 259.1452.

#### 3.1.29. *N*-hydroxy-7-(5-methyl-1*H*-indol-1-yl)heptanamide (**5g**)

Compound **5g** was prepared using similar procedures as for **3a** from compound **4g** (100 mg, 0.35 mmol). The crude product was purified by silica gel chromatography to give the brown solid (53 mg, 55%). ^1^H NMR (400 MHz, CDCl_3_) δ 7.42 (s, 1H), 7.22 (d, *J* = 8.3 Hz, 1H), 7.05–7.00 (overlap, 2H), 6.39 (d, *J* = 3.0 Hz, 1H), 4.03 (t, *J* = 7.0 Hz, 2H), 2.46 (s, 3H), 1.96 (t, *J* = 7.5 Hz, 2H), 1.80–1.70 (m, 2H), 1.56–1.46 (m, 2H), 1.28–1.20 (overlap, 4H). ^13^C NMR (100 MHz, CDCl_3_) δ 171.9, 134.4, 128.9, 128.5, 128.0, 123.1, 120.7, 109.2, 100.4, 46.3, 32.7, 30.0, 28.6, 26.5, 25.2, 21.5. HRMS (ESI), *m*/*z*: calcd for C_16_H_21_N_2_O_2_^−^: 273.1609 [M − H]^−^; found: 273.1608.

#### 3.1.30. 7-(6-Fluoro-1*H*-indol-1-yl)-*N*-hydroxyheptanamide (**5h**)

Compound **5h** was prepared using similar procedures as for **3a** from compound **4h** (296 mg, 1.02 mmol). The crude product was purified by silica gel chromatography to give the white solid (119 mg, 42%). ^1^H NMR (600 MHz, CDCl_3_) δ 7.50 (dd, *J* = 8.6, 5.4 Hz, 1H), 7.03 (d, *J* = 3.1 Hz, 1H), 6.98 (dd, *J* = 10.0, 2.3 Hz, 1H), 6.85 (td, *J* = 9.1, 2.2 Hz, 1H), 6.44 (d, *J* = 3.1 Hz, 1H), 4.00 (t, *J* = 7.1 Hz, 2H), 2.04 (t, *J* = 7.4 Hz, 2H), 1.76 (p, *J* = 7.0 Hz, 2H), 1.55 (p, *J* = 7.1 Hz, 2H), 1.30–1.20 (overlap, 4H). ^13^C NMR (150 MHz, CDCl_3_) δ 171.7, (160.6, 159.0), (136.1, 136.0), (128.4, 128.3), 125.1, (121.8, 121.7), (108.2, 108.0), 101.30, (95.9, 95.8), 46.5, 32.8, 29.9, 28.6, 26.6, 25.2. HRMS (ESI), *m*/*z*: calcd for C_15_H_18_FN_2_O_2_^−^: 277.1358 [M − H]^−^; found: 277.1356.

#### 3.1.31. 7-(5-Fluoro-1*H*-indol-1-yl)-*N*-hydroxyheptanamide (**5i**)

Compound **5i** was prepared using similar procedures as for **3a** from compound **4i** (499 mg, 1.71 mmol). The crude product was purified by silica gel chromatography to give the light brown solid (243 mg, 51%). ^1^H NMR (600 MHz, CDCl_3_) δ 7.25–7.23 (m, 1H), 7.21 (dd, *J* = 8.9, 4.3 Hz, 1H), 7.09 (d, *J* = 3.1 Hz, 1H), 6.94 (td, *J* = 9.1, 2.5 Hz, 1H), 6.42 (d, *J* = 3.0 Hz, 1H), 4.06 (t, *J* = 7.0 Hz, 2H), 2.04 (t, *J* = 7.5 Hz, 2H), 1.79 (p, *J* = 7.1 Hz, 2H), 1.57 (p, *J* = 7.4 Hz, 2H), 1.30–1.23 (overlap, 4H). ^13^C NMR (150 MHz, CDCl_3_) δ 171.5, (158.7 157.1), 132.7, 129.5, (128.9, 128.8), (110.1, 109.8), 110.0, (105.8, 105.7), (101.1, 101.0), 46.6, 32.8, 30.0, 28.7, 26.6, 25.1. HRMS (ESI), *m*/*z*: calcd for C_15_H_18_FN_2_O_2_^−^: 277.1358 [M − H]^−^; found: 277.1356.

#### 3.1.32. 7-(5-Chloro-1*H*-indol-1-yl)-*N*-hydroxyheptanamide (**5j**)

Compound **5j** was prepared using similar procedures as for **3a** from compound **4j** (305 mg, 0.99 mmol). The crude product was purified by silica gel chromatography to give the brown solid (70 mg, 24%). ^1^H NMR (600 MHz, CDCl_3_) δ 7.56 (d, *J* = 2.0 Hz, 1H), 7.20 (d, *J* = 8.6 Hz, 1H), 7.12 (dd, *J* = 8.6, 2.0 Hz, 1H), 7.05 (d, *J* = 3.0 Hz, 1H), 6.39 (d, *J* = 2.9 Hz, 1H), 3.98 (t, *J* = 7.0 Hz, 2H), 2.06–1.90 (m, 2H), 1.73–1.64 (m, 2H), 1.52–1.42 (m, 2H), 1.23–1.12 (overlap, 4H). ^13^C NMR (150 MHz, CDCl_3_) δ 172.0, 134.4, 129.6, 129.3, 125.0, 121.7, 120.3, 110.5, 100.7, 46.4, 32.7, 30.0, 28.5, 26.5, 25.2. HRMS (ESI), *m*/*z*: calcd for C_15_H_18_ClN_2_O_2_^−^: 293.1062 [M − H]^−^; found: 293.1062.

#### 3.1.33. 7-(5-Bromo-1*H*-indol-1-yl)-*N*-hydroxyheptanamide (**5k**)

Compound **5k** was prepared using similar procedures as for **3a** from compound **4k** (398 mg, 1.13 mmol). The crude product was purified by silica gel chromatography to give the brown solid (77 mg, 20%). ^1^H NMR (600 MHz, CDCl_3_) δ 7.73–7.68 (m, 1H), 7.26–7.19 (m, 1H), 7.15–7.11 (m, 1H), 7.01 (d, *J* = 2.8 Hz, 1H), 6.37 (d, *J* = 3.0 Hz, 1H), 3.97 (t, *J* = 7.1 Hz, 2H), 1.97 (t, *J* = 7.5 Hz, 2H), 1.68 (t, *J* = 7.3 Hz, 2H), 1.47 (p, *J* = 7.1 Hz, 2H), 1.21–1.11 (overlap, 4H). ^13^C NMR (150 MHz, CDCl_3_) δ 171.9, 134.7, 130.3, 129.1, 124.2, 123.4, 112.6, 111.0, 100.7, 46.4, 32.7, 30.0, 28.5, 26.5, 25.2. HRMS (ESI), *m*/*z*: calcd for C_15_H_18_BrN_2_O_2_^−^: 337.0557 [M − H]^−^; found: 337.0556.

#### 3.1.34. 7-(5-Cyano-1*H*-indol-1-yl)-*N*-hydroxyheptanamide (**5l**)

Compound **5l** was prepared using similar procedures as for **3a** from compound **4l** (409 mg, 1.37 mmol). The crude product was purified by silica gel chromatography to give the gray solid (43 mg, 11%). ^1^H NMR (600 MHz, CD_3_OD) δ 7.93–790 (m, 1H), 7.42 (d, *J* = 8.6 Hz, 1H), 7.37 (d, *J* = 8.6 Hz, 1H), 7.27 (d, *J* = 3.3 Hz, 1H), 6.54 (d, *J* = 3.2 Hz, 1H), 4.14 (t, *J* = 7.1 Hz, 2H), 2.05–2.00 (m, 2H), 1.83–1.76 (m, 2H), 1.56 (p, *J* = 7.4 Hz, 2H), 1.34–1.24 (overlap, 4H). ^13^C NMR (150 MHz, CD_3_OD) δ 171.0, 137.3, 130.0, 127.9, 125.9, 123.5, 120.3, 109.9, 101.5, 100.9, 45.8, 32.1, 29.5, 28.0, 25.9, 24.8. HRMS (ESI), *m*/*z*: calcd for C_16_H_18_N_3_O_2_^−^: 284.1405 [M − H]^−^; found: 284.1403.

#### 3.1.35. Methyl 4-(2-bromoethyl)benzoate (**7**)

To a solution of 4-(2-bromoethyl)benzoic acid (**6**) (500 mg, 2.19 mmol) in MeOH (10 mL) was added thionyl chloride (0.3 mL), and the resulting mixture was stirred under refluxing condition for 2 h. The reaction was complete detected by TLC. The solvent was evaporated under reduced pressure and diluted with H_2_O (20 mL). The mixture was extracted with ethyl acetate (3 × 10 mL). The combined organic layers were washed with brine, dried by anhydrous Na_2_SO_4_, filtered, and evaporated under reduced pressure to afford the crude product, which was purified by column chromatography eluting with petroleum ether/ethyl acetate (10:1) to give **7** as a colorless oil (513 mg, 97%). ^1^H NMR (400 MHz, CDCl_3_) δ 8.02–7.98 (overlap, 2H), 7.32–7.27 (overlap, 2H), 3.91 (s, 3H), 3.59 (t, *J* = 7.4 Hz, 2H), 3.23 (t, *J* = 7.4 Hz, 2H). ^13^C NMR (125 MHz, CDCl_3_) δ 167.0, 144.1, 130.0 × 2, 128.8 × 2, 128.5, 52.2, 39.2, 32.2.

#### 3.1.36. Methyl 4-(2-(2-acetyl-1*H*-pyrrol-1-yl)ethyl)benzoate (**8**)

To a solution of 2-acetyl pyrrole (196 mg, 1.80 mmol) in dry toluene (15 mL) was added potassium hydroxide (61 mg, 1.09 mmol) and 18-crown-6 (79 mg, 0.30 mmol). The reaction solution was refluxed and stirred for 1 h. Compound **7** (300 mg, 1.24 mmol) was added, and the mixture was stirred overnight. The reaction was complete detected by TLC. The solvent was evaporated under reduced pressure and diluted with H_2_O (20 mL). The mixture was extracted with ethyl acetate (3 × 15 mL). The combined organic layers were washed with brine, dried by anhydrous Na_2_SO_4_, filtered, and the solvent was evaporated under reduced pressure to afford the crude product, which was purified by column chromatography eluting with petroleum ether/ethyl acetate (10:1) to give **8** as a colorless oil (130 mg, 39%). ^1^H NMR (400 MHz, CDCl_3_) δ 7.96–7.87 (overlap, 2H), 7.21–7.12 (overlap, 2H), 6.98 (dd, *J* = 4.1, 1.7 Hz, 1H), 6.58 (t, *J* = 2.2 Hz, 1H), 6.05 (dd, *J* = 4.1, 2.5 Hz, 1H), 4.51 (t, *J* = 7.2 Hz, 2H), 3.90 (s, 3H), 3.07 (t, *J* = 7.3 Hz, 2H), 2.46 (s, 3H). ^13^C NMR (125 MHz, CDCl_3_) δ 188.5, 167.2, 144.1, 130.6, 130.0, 129.9 × 2, 129.2 × 2, 128.6, 120.8, 108.1, 52.2, 51.3, 38.2, 27.4.

#### 3.1.37. 4-(2-(2-Acetyl-1*H*-pyrrol-1-yl)ethyl)benzoic Acid (**9**)

To a solution of compound **8** (130 mg, 0.48 mmol) in H_2_O (2 mL) and MeOH (8 mL) was added lithium hydroxide (23 mg, 0.96 mmol). The reaction mixture was stirred at 40 °C until complete as indicated by TLC. Then the mixture was acidified by adding 1 M HCl solution and extracted with ethyl acetate (3 × 10 mL). The combined organic layers were washed with brine, dried by anhydrous Na_2_SO_4_, filtered, and concentrated under reduced pressure to give the crude product, which was further purified by column chromatography eluting with 2%–10% MeOH/DCM to give **9** as a white solid (100 mg, 81%). ^1^H NMR (400 MHz, CDCl_3_) δ 8.03–7.98 (overlap, 2H), 7.24–7.18 (overlap, 2H), 7.00 (dd, *J* = 4.0, 1.7 Hz, 1H), 6.59 (m, 1H), 6.06 (dd, *J* = 4.1, 2.5 Hz, 1H), 4.53 (t, *J* = 7.2 Hz, 2H), 3.10 (t, *J* = 7.2 Hz, 2H), 2.48 (s, 3H). ^13^C NMR (125 MHz, CDCl_3_) δ 188.6, 171.7, 145.1, 130.7, 130.6 × 2, 130.0, 129.3 × 2, 127.8, 120.9, 108.2, 51.2, 38.2, 27.4.

#### 3.1.38. 4-(2-(2-Acetyl-1*H*-pyrrol-1-yl)ethyl)-*N*-((tetrahydro-2H-pyran-2-yl)oxy) Benzamide (**10**)

To a solution of **9** (100 mg, 0.39 mmol) and *O*-(tetrahydro-2*H*-pyran-2-yl)hydroxylamine (91 mg, 0.78 mmol) in dichloromethane (10 mL) was added HATU (372 mg, 0.98 mmol) and DIPEA (126 mg, 0.98 mmol). The resulting mixture was stirred at room temperature overnight. The reaction was complete detected by TLC. The reaction mixture was then quenched with H_2_O (20 mL). The mixture was extracted with ethyl acetate (3 × 15 mL). The combined organic layers were washed with brine, dried by anhydrous Na_2_SO_4_, filtered, and concentrated under reduced pressure to afford the crude product, which was purified by column chromatography eluting with 1%–5% DCM/MeOH to give **10** as a colorless oil (50 mg, 36%). ^1^H NMR (400 MHz, CDCl_3_) δ 9.09 (s, 1H), 7.68–7.62 (overlap, 2H), 7.18–7.13 (overlap, 2H), 6.97 (d, *J* = 4.1 Hz, 1H), 6.57 (t, *J* = 2.1 Hz, 1H), 6.03 (t, *J* = 3.3 Hz, 1H), 5.09–5.02 (m, 1H), 4.48 (t, *J* = 7.2 Hz, 2H), 4.03–3.95 (m, 1H), 3.65–3.59 (m, 1H), 3.03 (t, *J* = 7.2 Hz, 2H), 2.45 (s, 3H), 1.91–1.81 (overlap, 3H), 1.68–1.55 (overlap, 3H). ^13^C NMR (150 MHz, CDCl_3_) δ 188.6, 166.0, 143.1, 130.7 × 2, 130.2, 129.9, 129.4 × 2, 127.5, 120.9, 108.2, 102.8, 62.9, 51.3, 38.0, 28.2, 27.4, 25.1, 18.8.

#### 3.1.39. 4-(2-(2-Acetyl-1*H*-pyrrol-1-yl)ethyl)-*N*-hydroxybenzamide (**11**)

To a solution of compound **10** (50 mg, 0.14 mmol) in MeOH (6 mL) was added *p*-toluenesulfonic acid (2.5 mg, 0.014 mmol). The reaction mixture was stirred at room temperature for 3 h. The reaction was complete detected by TLC. The solvent was evaporated under reduced pressure. The residue was pulped with dichloromethane and ethyl acetate (1:1) and filtered to give **11** as a brown solid (12 mg, 32%). ^1^H NMR (400 MHz, CD_3_OD) δ 7.68–7.58 (overlap, 2H), 7.25–7.17 (overlap, 2H), 7.11 (d, *J* = 4.2 Hz, 1H), 6.85–6.78 (m, 1H), 6.08–6.03 (m, 1H), 4.54 (t, *J* = 7.2 Hz, 2H), 3.03 (t, *J* = 7.2 Hz, 2H), 2.43 (s, 3H). ^13^C NMR (125 MHz, CD_3_OD) δ 190.5, 144.1, 132.7, 131.7, 131.0, 130.3 × 2, 128.2 × 2, 122.9, 109.3, 51.9, 38.8, 27.1. HRMS (ESI), *m*/*z*: calcd for C_15_H_15_N_2_O^−^: 271.1088 [M − H]^−^; found: 271.1087.

#### 3.1.40. Methyl (*E*)-3-(4-((2-acetyl-1*H*-pyrrol-1-yl)methyl)phenyl)acrylate (**13**)

Starting from methyl (*E*)-3-(4-(bromomethyl)phenyl)acrylate (**12**) (278 mg, 1.09 mmol) and 2-acetyl pyrrole (99 mg, 0.91 mmol), the title compound was obtained following the procedure previously described for compound **2a**. The crude product was purified by silica gel chromatography to give the colorless oil **13** (206 mg, 80%). ^1^H NMR (600 MHz, CDCl_3_) δ 7.64 (d, *J* = 16.0 Hz, 1H), 7.45–7.42 (overlap, 2H), 7.11–7.07 (overlap, 2H), 7.02 (dd, *J* = 4.1, 1.7 Hz, 1H), 6.92 (dd, *J* = 2.6, 1.7 Hz, 1H), 6.39 (d, *J* = 16.0 Hz, 1H), 6.21 (dd, *J* = 4.1, 2.6 Hz, 1H), 5.58 (s, 2H), 3.79 (s, 3H), 2.40 (s, 3H). ^13^C NMR (150 MHz, CDCl_3_) δ 188.5, 167.5, 144.5, 140.9, 133.7, 130.6, 130.4, 128.5 × 2, 127.5 × 2, 120.6, 117.8, 108.9, 52.5, 51.8, 27.4.

#### 3.1.41. (*E*)-3-(4-((2-acetyl-1*H*-pyrrol-1-yl)methyl)phenyl)-*N*-hydroxyacrylamide (**14**)

Starting from **13** (43 mg, 0.15 mmol), the title compound was obtained following the procedure previously described for compound **11**. All the intermediates were only extracted and dried without further purification. The final crude product was purified by silica gel chromatography to give the white solid **14** (15 mg, 35% yield over three steps). ^1^H NMR (500 MHz, CD_3_OD) δ 7.52 (d, *J* = 15.8 Hz, 1H), 7.48–7.43 (overlap, 2H), 7.18–7.14 (overlap, 2H), 7.08–7.05 (overlap, 2H), 6.42 (d, *J* = 15.8 Hz, 1H), 6.24 (dd, *J* = 4.0, 2.7 Hz, 1H), 5.59 (s, 2H), 2.37 (s, 3H). ^13^C NMR (125 MHz, CD_3_OD) δ 190.4, 166.3, 142.2, 141.2, 135.2, 133.0, 131.3, 128.9 × 2, 128.3 × 2, 122.8, 118.3, 109.9, 53.2, 27.0. HRMS (ESI), *m*/*z*: calcd for C_16_H_15_N_2_O^−^: 283.1088 [M − H]^−^; found: 283.1086.

#### 3.1.42. 1-(1-(Prop-2-yn-1-yl)-1*H*-pyrrol-2-yl)ethanone (**16**)

Starting from propargyl bromide (**15**) (577 mg, 4.85 mmol) and 2-acetyl pyrrole (440 mg, 4.04 mmol), the title compound was obtained following the procedure previously described for compound **2a**. The crude product was purified by silica gel chromatography to give the colorless oil **16** (529 mg, 89%). ^1^H NMR (400 MHz, CDCl_3_) δ 7.17 (t, *J* = 2.2 Hz, 1H), 6.97 (dd, *J* = 4.1, 1.7 Hz, 1H), 6.18 (dd, *J* = 4.1, 2.6 Hz, 1H), 5.19 (d, *J* = 2.5 Hz, 2H), 2.43–2.40 (overlap, 4H). ^13^C NMR (100 MHz, CDCl_3_) δ 188.7, 130.0, 129.3, 120.6, 108.7, 78.3, 74.0, 38.9, 27.2.

#### 3.1.43. 4-(2-Azidoethyl)benzoic Acid (**17**)

To a solution of 4-(2-bromoethyl)benzoic acid (**6**) (684 mg, 3.00 mmol) in THF (25 mL) was added azido trimethyl silane (518 mg, 4.50 mmol) and tetrabutylammonium fluoride trihydrate (1.26 g, 4.50 mmol). The mixture was stirred at room temperature overnight. Upon completion of the reaction, acetic acid (1 mL) and H_2_O (15 mL) were added. The mixture was extracted with ethyl acetate (3 × 15 mL). The combined organic layers were washed with brine, dried by anhydrous Na_2_SO_4_, filtered, and concentrated in vacuo. The residue was purified by column chromatography eluting with petroleum ether/ethyl acetate (5:1) to give **17** as a yellow oil (558 mg, 97%). ^1^H NMR (400 MHz, CDCl_3_) δ 8.11–8.05 (overlap, 2H), 7.36–7.30 (overlap, 2H), 3.56 (t, *J* = 7.1 Hz, 2H), 2.97 (t, *J* = 7.1 Hz, 2H). ^13^C NMR (100 MHz, CDCl_3_) δ 172.4, 144.6, 130.7 × 2, 129.1 × 2, 128.0, 52.0, 35.5.

#### 3.1.44. 4-(2-(4-((2-Acetyl-1*H*-pyrrol-1-yl)methyl)-1*H*-1,2,3-triazol-1-yl)ethyl)benzoic Acid (**18**)

To a solution of compounds **16** (441 mg, 3.00 mmol) and **17** (478 mg, 2.50 mmol) in DMF (30 mL) was added copper (II) sulfate pentahydrate (31 mg, 0.125 mmol) and sodium L-ascorbate (50 mg, 0.25 mmol) dissolved in H_2_O (10 mL). The reaction mixture was stirred at room temperature overnight. The reaction was complete detected by TLC. Then, H_2_O (30 mL) was added, and the mixture was extracted with ethyl acetate (3 × 15 mL). The combined organic layers were washed with brine, dried by anhydrous Na_2_SO_4_, filtered, and concentrated in vacuo, affording the crude product, which was purified by column chromatography eluting with 1%–5% DCM/MeOH to give **18** as a yellow oil (740 mg, 88%). ^1^H NMR (400 MHz, CDCl_3_) δ 8.00–7.94 (overlap, 2H), 7.49 (s, 1H), 7.19–7.15 (m, 1H), 7.14–7.09 (overlap, 2H), 6.98 (dd, *J* = 4.1, 1.7 Hz, 1H), 6.16 (dd, *J* = 4.1, 2.6 Hz, 1H), 5.59 (s, 2H), 4.56 (t, *J* = 7.2 Hz, 2H), 3.25 (t, *J* = 7.2 Hz, 2H), 2.42 (s, 3H). ^13^C NMR (100 MHz, CDCl_3_) δ 189.0, 170.2, 144.4, 142.7, 131.2, 130.7 × 2, 129.5, 128.9, 128.8 × 2, 123.8, 121.2, 109.0, 51.3, 44.1, 36.8, 27.2.

#### 3.1.45. 4-(2-(4-((2-Acetyl-1*H*-pyrrol-1-yl)methyl)-1*H*-1,2,3-triazol-1-yl)ethyl)-*N*-(2-aminophenyl)benzamide (**19a**)

To a solution of **18** (34 mg, 0.10 mmol) and 1,2-diaminobenzene (11 mg, 0.10 mmol) in dichloromethane (10 mL) was added EDCI (23 mg, 0.12 mmol) and DMAP (1.22 mg, 0.01 mmol). The reaction mixture was stirred at room temperature until complete as indicated by TLC. Then, the mixture was quenched with H_2_O (15 mL) and extracted with dichloromethane (3 × 10 mL). The combined organic layers were washed with brine, dried by Na_2_SO_4_, filtered, and then concentrated under reduced pressure to give the crude product, which was purified by column chromatography eluting with petroleum ether/ethyl acetate (3:1) to give **19a** as a light brown solid (15 mg, 35%). ^1^H NMR (400 MHz, CDCl_3_) δ 8.18 (s, 1H), 7.79–7.72 (overlap, 2H), 7.31 (d, *J* = 7.9 Hz, 1H), 7.27 (s, 1H), 7.12–7.09 (overlap, 4H), 6.96 (dd, *J* = 4.1, 1.7 Hz, 1H), 6.87–6.80 (overlap, 2H), 6.16 (dd, *J* = 4.1, 2.6 Hz, 1H), 5.54 (s, 2H), 4.53 (t, *J* = 7.0 Hz, 2H), 3.22 (t, *J* = 7.0 Hz, 2H), 2.39 (s, 3H). ^13^C NMR (125 MHz, CDCl_3_) δ 188.9, 165.6, 144.5, 141.2, 140.7, 133.3, 131.1, 129.7, 129.1 × 2, 128.0 × 2, 127.4, 125.3, 124.7, 123.5, 121.0, 119.9, 118.6, 109.0, 51.4, 44.3, 36.6, 27.3. HRMS (ESI), *m*/*z*: calcd for C_24_H_23_N_6_O_2_^−^: 427.1888 [M − H]^−^; found: 427.1888.

#### 3.1.46. 4-(2-(4-((2-Acetyl-1*H*-pyrrol-1-yl)methyl)-1*H*-1,2,3-triazol-1-yl)ethyl)-*N*-(2-amino-4-fluorophenyl)benzamide (**19b**)

Compound **19b** was prepared using similar procedures as for **19a** from compound **18** (34 mg, 0.10 mmol) and 3,4-diaminofluorobenzene (12.6 mg, 0.10 mmol). The crude product was purified by silica gel chromatography to give the brown solid (23 mg, 51%). ^1^H NMR (400 MHz, CDCl_3_) δ 8.07 (s, 1H), 7.77–7.71 (overlap, 2H), 7.26 (s, 1H), 7.16 (dd, *J* = 8.6, 5.8 Hz, 1H), 7.11–7.05 (overlap, 3H), 6.96 (dd, *J* = 4.0, 1.7 Hz, 1H), 6.54–6.47 (overlap, 2H), 6.16 (dd, *J* = 4.1, 2.6 Hz, 1H), 5.54 (s, 2H), 4.54 (t, *J* = 7.0 Hz, 2H), 3.22 (t, *J* = 7.0 Hz, 2H), 2.39 (s, 3H). ^13^C NMR (125 MHz, CDCl_3_) δ 189.0, 166.0, (163.0, 161.1), 144.6, (143.1, 143.0), 141.4, 133.0, 131.2, 129.7, 129.2 × 2, 128.0 × 2, (127.4, 127.3), 123.5, 121.1, 120.1, 109.0, (106.3, 106.1), (104.7, 104.5), 51.4, 44.3, 36.6, 27.3. HRMS (ESI), *m*/*z*: calcd for C_24_H_22_FN_6_O_2_^−^: 445.1794 [M − H]^−^; found: 445.1794.

#### 3.1.47. 4-(2-(4-((2-Acetyl-1*H*-pyrrol-1-yl)methyl)-1*H*-1,2,3-triazol-1-yl)ethyl)-*N*-(2-amino-4-chlorophenyl)benzamide (**19c**)

Compound **19c** was prepared using similar procedures as for **19a** from compound **18** (34 mg, 0.10 mmol) and 4-chloro-*o*-phenylenediamine (14.2 mg, 0.10 mmol). The crude product was purified by silica gel chromatography to give the brown solid (12 mg, 26%). ^1^H NMR (400 MHz, CDCl_3_) δ 8.16 (s, 1H), 7.77–7.72 (overlap, 2H), 7.24 (s, 1H), 7.21 (d, *J* = 8.4 Hz, 1H), 7.11–7.09 (m, 1H), 7.09–7.05 (overlap, 2H), 6.97 (dd, *J* = 4.1, 1.7 Hz, 1H), 6.83 (d, *J* = 2.3 Hz, 1H), 6.78 (d, *J* = 8.3, 2.2 Hz, 1H), 6.16 (dd, *J* = 4.1, 2.5 Hz, 1H), 5.54 (s, 2H), 4.54 (t, *J* = 7.0 Hz, 2H), 3.23 (t, *J* = 7.0 Hz, 2H), 2.39 (s, 3H). ^13^C NMR (150 MHz, CDCl_3_) δ 189.0, 166.0, 144.5, 142.1, 141.4, 132.9, 132.5, 131.2, 129.6, 129.1 × 2, 128.0 × 2, 126.7, 123.5, 123.1, 121.1, 119.6, 117.9, 109.0, 51.3, 44.3, 36.6, 27.3. HRMS (ESI), *m*/*z*: calcd for C_24_H_22_ClN_6_O_2_^−^: 461.1498 [M − H]^−^; found: 461.1500.

#### 3.1.48. 4-(2-(4-((2-Acetyl-1*H*-pyrrol-1-yl)methyl)-1*H*-1,2,3-triazol-1-yl)ethyl)-*N*-(2-amino-4,5-difluorophenyl)benzamide (**19d**)

Compound **19d** was prepared using similar procedures as for **19a** from compound **18** (34 mg, 0.10 mmol) and 4,5-difluorophenylene-1,2-diamine (14.4 mg, 0.10 mmol). The crude product was purified by silica gel chromatography to give the brown solid (16 mg, 35%). ^1^H NMR (400 MHz, CDCl_3_) δ 8.32 (s, 1H), 7.78–7.70 (overlap, 2H), 7.27 (s, 1H), 7.11–7.05 (overlap, 3H), 6.97 (dd, *J* = 4.1, 1.6 Hz, 1H), 6.68–6.60 (m, 1H), 6.15 (dd, *J* = 4.1, 2.5 Hz, 1H), 5.53 (s, 2H), 4.53 (t, *J* = 6.9 Hz, 2H), 3.22 (t, *J* = 7.0 Hz, 2H), 2.39 (s, 3H). ^13^C NMR (150 MHz, CDCl_3_) δ 189.0, 165.9, 147.6, 144.6, 142.7, 141.5, 137.0, 132.8, 131.2, 129.6, 129.2 × 2, 128.0 × 2, 123.4, 121.2, (120.7, 120.6), (114.2, 114.0), 109.0, (106.8, 106.6), 51.3, 44.3, 36.6, 27.3. HRMS (ESI), *m*/*z*: calcd for C_24_H_21_F_2_N_6_O_2_^−^: 463.1700 [M − H]^−^; found: 463.1700.

#### 3.1.49. 4-(2-(4-((2-Acetyl-1*H*-pyrrol-1-yl)methyl)-1*H*-1,2,3-triazol-1-yl)ethyl)-*N*-(2-amino-4-methoxyphenyl)benzamide (**19e**)

Compound **19e** was prepared using similar procedures as for **19a** from compound **18** (34 mg, 0.10 mmol) and 4-methoxy-*o*-phenylenediamine (13.8 mg, 0.10 mmol). The crude product was purified by silica gel chromatography to give the brown solid (28 mg, 62%). ^1^H NMR (400 MHz, CDCl_3_) δ 8.13 (s, 1H), 7.77–7.71 (overlap, 2H), 7.29 (s, 1H), 7.10–7.03 (overlap, 4H), 6.96 (dd, *J* = 4.1, 1.7 Hz, 1H), 6.39–6.31 (m, 2H), 6.15 (dd, *J* = 4.1, 2.6 Hz, 1H), 5.53 (s, 2H), 4.52 (t, *J* = 7.0 Hz, 2H), 3.75 (s, 3H), 3.20 (t, *J* = 7.0 Hz, 2H), 2.39 (s, 3H). ^13^C NMR (100 MHz, CDCl_3_) δ 188.9, 166.1, 159.2, 144.5, 143.0, 141.0, 133.1, 131.1, 129.6, 129.0 × 2, 128.0 × 2, 127.2, 123.5, 121.0, 117.2, 108.9, 104.9, 103.0, 55.5, 51.3, 44.2, 36.6, 27.3. HRMS (ESI), *m*/*z*: calcd for C_25_H_25_N_6_O_3_^−^: 457.1994 [M − H]^−^; found: 457.1992.

#### 3.1.50. Methyl 4-(4-(2-(4-((2-acetyl-1*H*-pyrrol-1-yl)methyl)-1*H*-1,2,3-triazol-1-yl)ethyl)benzamido)-3-aminobenzoate (**19f**)

Compound **19f** was prepared using similar procedures as for **19a** from compound **18** (34 mg, 0.10 mmol) and methyl 3,4-diaminobenzoate (16.6 mg, 0.10 mmol). The crude product was purified by silica gel chromatography to give the brown solid (12 mg, 25%). ^1^H NMR (400 MHz, CD_3_OD) δ 7.85–7.82 (overlap, 3H), 7.72 (dd, *J* = 8.5, 2.0 Hz, 1H), 7.53 (s, 1H), 7.18–7.14 (overlap, 2H), 7.13–7.11 (m, 1H), 7.11–7.08 (m 1H), 6.85 (d, *J* = 8.5 Hz, 1H), 6.18 (dd, *J* = 4.2, 2.5 Hz, 1H), 5.55 (s, 2H), 4.65 (t, *J* = 6.8 Hz, 2H), 3.83 (s, 3H), 3.25 (t, *J* = 6.8 Hz, 2H), 2.39 (s, 3H). ^13^C NMR (125 MHz, CD_3_OD) δ 190.6, 169.0, 168.7, 149.8, 143.1, 133.9, 133.6, 132.4, 130.9, 130.5 × 2, 130.0 × 2, 129.2 × 2, 125.0, 123.1, 122.7, 119.4, 116.5, 110.0, 52.2 × 2, 44.9, 37.2, 27.1. HRMS (ESI), *m*/*z*: calcd for C_26_H_25_N_6_O_4_^−^: 485.1943 [M − H]^−^; found: 485.1945.

#### 3.1.51. 4-(2-(2-Acetyl-1*H*-pyrrol-1-yl)ethyl)-*N*-(2-aminophenyl)benzamide (**20**)

Compound **20** was prepared using similar procedures as for **10** from compound **9** (32 mg, 0.12 mmol) and 1,2-diaminobenzene (26 mg, 0.24 mmol). The crude product was purified by silica gel chromatography to give the light brown solid (27 mg, 65%). ^1^H NMR (400 MHz, CDCl_3_) δ 7.85–7.77 (overlap, 2H), 7.32 (d, *J* = 7.8 Hz, 1H), 7.23–7.18 (overlap, 2H), 7.11–7.06 (m, 1H), 6.99 (dd, *J* = 4.0, 1.7 Hz, 1H), 6.92–6.84 (overlap, 2H), 6.61 (t, *J* = 2.2 Hz, 1H), 6.06 (dd, *J* = 4.1, 2.5 Hz, 1H), 4.51 (t, *J* = 7.3 Hz, 2H), 3.07 (t, *J* = 7.2 Hz, 2H), 2.47 (s, 3H). ^13^C NMR (150 MHz, CDCl_3_) δ 188.6, 166.0, 143.1, 139.4, 132.3, 130.7 × 2, 130.0, 129.5 × 2, 127.7, 127.4, 125.5, 125.3, 120.9, 120.8, 119.1, 108.2, 51.3, 38.0, 27.4. HRMS (ESI), *m*/*z*: calcd for C_21_H_20_N_3_O_2_^−^: 346.1561 [M − H]^−^; found: 346.1560.

### 3.2. Biological Evaluations

#### 3.2.1. HDAC Inhibition Assay

HDAC1 (#50001, BPS, San Diego, USA), HDAC3 (#50003, BPS, San Diego, CA, USA) or HDAC6 (#50006, BPS, San Diego, USA), H3(1-21)K9Ac (#AS-64361, Anaspec, Fremont, CA, USA) substrate and test compounds were added into each well (1 × Enzymatic buffer preparation: 50 mM Tris-HCl pH 8.0, 137 mM NaCl, 2.7 mM KCl, 1 mM MgCl_2_, 0.01% Tween20), incubated at room temperature for 1 h in the dark. Then, SA-XL665 (#610SAXLG, Cisbio, Bedford, MA, USA) and anti-H3K9me0-Eu(K) (#61KB0KAD, Cisbio, USA) were added. A multi-label microplate analysis system (PerkinElmer Envision, Waltham, MA, USA) detected the fluorescence values at 620 nm and 665 nm, and calculated the HTRF signal ratio (665 nm/620 nm) of each well. The IC_50_ value of the compounds was calculated using GraphPad 7.0 software. Each set of experiments was independently repeated twice, with two replicate wells per concentration each time. The results were displayed as Mean ± SD.

#### 3.2.2. Cell Viability Assay

The CCK8 (#AC11L054, Life ilab, Shanghai, China) method was used to detect the cells’ proliferation. Cells were seeded into a 96-well culture plate at an appropriate density. After treatment with different concentrations of agents for 72 h, 10 μL of CCK8 was added to each well, and their optical density (OD value) was measured at a wavelength of 450 nm using SpectraMax 190 (Sunnyvale, CA, USA) microplate reader. IC_50_ values were estimated using the four-parameter method. Each set of experiments was repeated independently three times with three replicate wells per concentration each time. The results were displayed as Mean ± SD.

#### 3.2.3. Western Immunoblotting

RPMI-8226 cells were seeded in six-well plates and treated with compounds for 24 h. Cells were lysed with 1× SDS loading buffer (50 mM Tris pH 6.8, 100 mM DTT, 2% SDS, 0.1% bromophenol blue, 10% glycerol), and then centrifuged at 12,000 rpm for 5 min at 4 °C. Cell extracts were probed with antibodies Ac-Tubulin (#5335T, CST, Kansas City, MO, USA), α-Tubulin (#2144S, CST, Kansas City, MO, USA), AcH3 (#ab39139, active motif, Carlsbad, CA, USA), Histone H3 (#17168-1-AP, Proteintech, Chicago, IL, USA), GAPDH (#60004-1-1g, Proteintech, Chicago, IL, USA), Goat Anti-Mouse IgG, H&L Chain Specific Peroxidase Conjugate (#401215, Calbiochem, Darmstadt, Germany), Goat Anti-Rabbit IgG, or H&L Chain Specific Peroxidase Conjugate (#401353, Calbiochem, Germany), then detected using Bio-RAD power pal Basic (#041BR66615, BioRad Laboratories, Hercules, CA, USA), Bio-RAD Mini-PROTEAN Tetrade System (#IEC/EN 61010-1, BioRad Laboratories, Hercules, CA, USA), or Bio-RAD Chemi Touch Imagin (BioRad Laboratories, Hercules, CA, USA).

#### 3.2.4. Apoptosis Assay

RPMI-8226 cells were treated with indicated compounds for 48 h. Annexin V-FITC and PI double-staining detection kits were used to determine apoptotic cells. All stained cells analyzed using BD FlowJo 10.8 software and Annexin V+/PI− is regarded as early apoptotic cells, while Annexin V+/PI+ (A211-02, Vazyme, Nanjing, China) is regarded as late apoptotic cells. Data results were obtained from three independent experiments.

### 3.3. Molecular Docking and Molecular Dynamics Simulation Studies

The docking was conducted in AutoDock4.2. The PDB code 1C3S was downloaded from the Protein Data Bank for molecular docking. In the Protein Preparation Wizard, HDAC1 protein was prepared by removing H_2_O and adding hydrogen. Results analysis was presented using PyMol (http://www.pymol.org/, accessed on 7 May 2024) and Discovery Studio 4.5. Molecular dynamics simulations were performed using Desmond.

### 3.4. Pharmacokinetic Analysis

Specific procedures were referred to the reported method [48]. ICR mice (*n* = 6) were fasted for 12 h before administration and remained fasting for 2 h. The animal room environment was controlled (target conditions: temperature 18 to 29 °C, relative humidity 30 to 70%). Temperature and relative humidity were monitored daily. An electronic time-controlled lighting system was used to provide a 12 h light/12 h dark cycle. Compound **20** was administered at doses of 5 mg/kg (po) and 1 mg/kg (iv). The time points for blood sample collection were 0.25, 0.50, 1.00, 2.00, 4.00, 8.00, and 24 h post dose for the po-administrated group, and the time points were 0.05, 0.25, 0.75, 2.00, 4.00, 8.00, and 24 h for the iv group. For each point, 20 μL of plasma were transferred into a clean tube, 200 μL of MeOH/ACN (50/50, *v*/*v*) containing IS were added, and they were thoroughly vortexed by a vortex mixer for 1 min. The mixture was centrifuged at 15000 rpm for 5 min, and then 20 μL of supernatant was mixed with 20 μL of water for the analysis by LC-MS.

## 4. Conclusions

In this study, a new class of HDACis bearing the natural product-inspired *N*-linked 2-acetylpyrrole cap group were designed, synthesized, and biologically evaluated leveraging the molecular hybridization strategy. Most of the synthetic compounds exhibited significant HDAC inhibitory effects and potent anti-proliferation against cancer cells. Among them, compound **20** showed high potency against RPMI-8226 cells with an IC_50_ value of 2.89 ± 0.43 μM, which was better than that of chidamide (IC_50_ = 10.23 ± 1.02 μM). Compound **20** promoted apoptosis in RPMI-8226 cells in a dose-dependent manner, exhibiting a superior inhibitory effect compared to the identical concentration of chidamide. The molecular docking study revealed that **20** could bind tightly to the active site of HDAC1, and the hydrogen bond connecting the carbonyl oxygen of the 2-acetylpyrrole cap group with Phe198 underscores the significance of incorporating this natural product-inspired cap group. The results of molecular dynamics simulations indicated a robust conformational stability in the docked complex. Additionally, **20** possesses drug similarity and bioavailability scores suitable for in vivo experiments, as determined by the SwissADME algorithm. Similar to entinostat (MS-275) [48], preliminary pharmacokinetic evaluations showed that compound **20**’s properties did not fully meet expectations, thereby limiting its further in vivo efficacy studies. Nevertheless, **20** can serve as a promising lead compound for further optimization in the development of novel HDAC-targeted anticancer agents.

Most medicinal chemistry modifications of HDACis centered on the cap and linker parts. The cap group extending into the solvent region offers versatility for substitution with diverse structures aimed at enhancing the efficacy and pharmacokinetic properties. Meanwhile, the linker’s design can modulate molecular flexibility and accessibility to the zinc ion within the enzyme active site [39]. With this understanding, we will use **20** as the lead compound and prioritize modifications to both the cap group and linker to develop new HDACis that widen the therapeutic window and optimize pharmacokinetic properties.

## Figures and Tables

**Table 1:** Structures of compounds **3a**–**e**, **11**, and **14**, and their inhibitory activities against HDACs.

Compound	IC_50_ ± SD (nM)		
	HDAC1	HDAC3	HDAC6
**3a**	>10,000	>10,000	>10,000
**3b**	426.05 ± 13.79	>10,000	>10,000
**3c**	117.52 ± 30.81	137.85 ± 18.63	82.31 ± 15.16
**3d**	51.91 ± 6.73	45.53 ± 0.23	14.25 ± 1.24
**3e**	82.37 ± 31.15	84.96 ± 1.21	15.48 ± 2.90
**11** ^a^	77.60	79.90	89.45
**14**	800.35 ± 50.35	457.28 ± 172.99	33.68 ± 14.78
**SAHA**	13.37 ± 2.14	9.78 ± 1.28	6.84 ± 1.99

^a^ (%) Inhibition of three HDAC isomers at 10 μM.

**Table 2:** Structures of compounds **5a**–**l** and their inhibitory activities against HDACs.

Compound	IC_50_ ± SD (nM)		
	HDAC1	HDAC3	HDAC6
**5a**	101.47 ± 25.71	129.00 ± 51.61	16.37 ± 6.31
**5b**	54.17 ± 8.74	58.16 ± 4.64	7.67 ± 1.06
**5c**	36.84 ± 5.87	26.56 ± 1.90	4.22 ± 0.10
**5d**	51.98 ± 21.86	49.50 ± 2.92	11.18 ± 4.76
**5e**	13.73 ± 4.39	3.08 ± 0.97	9.74 ± 6.37
**5f** ^a^	96.85	94.40	92.60
**5g** ^a^	96.10	93.45	91.55
**5h** ^a^	96.45	93.25	92.40
**5i**	4.48 ± 2.43	1.82 ± 0.78	≤1.70
**5j** ^a^	96.25	93.35	92.35
**5k** ^a^	96.20	92.15	90.30
**5l**	2.42 ± 0.36	1.18 ± 0.06	2.31 ± 0.34
**SAHA**	13.37 ± 2.14	9.78 ± 1.28	6.84 ± 1.99

^a^ (%) Inhibition of three HDAC isomers at 1 μM.

**Table 3:** Structures of compounds **19a**–**f**, and **20**, and their inhibitory activities against HDACs.

Compound	IC_50_ ± SD (nM)		
	HDAC1	HDAC3	HDAC6
**19a**	1096.84 ± 143.67	1394.09 ± 373.05	>10,000
**19b**	>10,000	>10,000	>10,000
**19c**	>10,000	>10,000	>10,000
**19d**	>10,000	>10,000	>10,000
**19e**	>10,000	>10,000	>10,000
**19f**	>10,000	>10,000	>10,000
**20**	441.26 ± 78.15	1076.99 ± 229.06	>10,000
**Chidamide**	156.57 ± 46.83	1000.32 ± 37.84	Not determined

**Table 4:** Proliferation inhibition of compounds **3d**, **5a**–**l**, **11**, **14**, **19a**, and **20** against cancer cells.

Compound	IC_50_ ± SD (μM)		
	HCT-116	HL-60	RPMI-8226
**3d**	>30	>30	>30
**5a**	26.39 ± 4.1	19.83 ± 1.88	>30
**5b**	23.02 ± 7.03	12.93 ± 0.75	19.9 ± 7.71
**5c**	12.35 ± 2.20	8.56 ± 1.47	11.89 ± 1.58
**5d**	>30	21.84 ± 10.69	>30
**5e**	19.84 ± 8.32	7.5 ± 1.55	17.94 ± 6.51
**5f**	>30	>30	>30
**5g**	>30	>30	>30
**5h**	>30	>30	>30
**5i**	>30	>30	>30
**5j**	>30	>30	>30
**5k**	>30	>30	>30
**5l**	>30	>30	>30
**11**	17.12 ± 8.69	28.03 ± 0.56	11.66 ± 1.75
**14**	8.63 ± 0.82	8.16 ± 1.96	8.85 ± 2.30
**19a**	13.23 ± 2.48	8.32 ± 0.37	10.04 ± 0.52
**20**	9.08 ± 0.58	4.71 ± 0.24	2.89 ± 0.43
**Chidamide**	4.67 ± 0.53	0.5 ± 0.08	10.23 ± 1.02
**SAHA**	0.75 ± 0.05	0.72 ± 0.08	0.90 ± 0.40

**Table 5:** Prediction of physicochemical properties and pharmacokinetic properties for “drug-likeness” for **20** and chidamide.

Compound	MW	Rb	HBA	HBD	Bs	TPSA	CLogP	GI	P-gp	Drug-Like
**20**	347.41	7	2	2	0.55	77.12	2.86	High	Yes	Yes
**Chidamide**	390.41	8	4	3	0.55	97.11	2.74	High	Yes	Yes

MW: molecular weight; Rb: number of rotatable bonds; HBA: number of H-bond acceptors; HBD: number of H-bond donors; Bs: bioavailability score; TPSA: topological polar surface area in Å^2^ (≤140 Å^2^); CLogP: consensus LogP (average of iLOGP, XLOGP3, WLOGP, MLOGP and Silicos-IT LogP); GI: gastrointestinal absorption; P-gp: P-glycoprotein substrates; Drug-like: drug-likeness indices from Lipinski (Pfizer), Veber (GSK), Ghose (Amgen), Muegge (Bayer), and Egan (Pharmacia) filters.

**Table 6:** Pharmacokinetic parameters of compound **20** in vivo ^a^.

20	ICR Mice (*n* = 6)	
	iv	po
dose (mg/kg) ^b^	1	5
*T*_1/2_ (h)	0.192 ± 0.012	1.55 ± 0.51
*C*_max_ (ng/mL)		48.3 ± 31.1
*T*_max_ (h)		0.333 ± 0.144
AUC_last_ (h * ng/mL)	163 ± 3	40.7 ± 21.8
AUC_INF_ (h * ng/mL)	163 ± 3	47.2 ± 26.6
CL (mL/min/kg)	102 ± 2	
MRT_INF_ (h)	0.181 ± 0.006	1.84 ± 0.42
*V*_ss_ (mL/kg)	1112 ± 52	
*F* (%)		5.00

^a^ **20**: average values ± SD of six mice (iv dose and po dose). ^b^ Solution administration, po formulation: 0.5% MC; iv formulation: 5% DMSO, 5% EtOH, PEG300 40%, saline 50%.

## Data Availability

Data are contained within the article and Supplementary Materials.

## Supplementary Materials

The following supporting information can be downloaded at: https://www.mdpi.com/article/10.3390/molecules29194653/s1, Figure S1: Prediction of the physicochemical properties of compound **20** and chidamide, Figures S2–S79: NMR and HR-MS spectrum of all target compounds. Figure S80. The whole un-cropped images of the original western blots in Figure 3. Figure S81. The STR identification results of the three cells were correct. Table S1. General information about the cells used in the experiments.
