# Supplementary material for: Design, Synthesis, and Biological Evaluation of HDAC Inhibitors Containing Natural Product-Inspired N-Linked 2-Acetylpyrrole Cap
Source: Molecules. 2024 Sep 30;29(19):4653. doi: 10.3390/molecules29194653 (PMC11477621; doi:10.3390/molecules29194653)
Supplement: Supplementary file 1 [file molecules-29-04653-s001.zip › molecules-3147440-supplementary.pdf]

Supplementary Materials

Design, Synthesis, and Biological Evaluation of HDAC Inhibitors Containing Natural Product-Inspired *N*-Linked 2-Acetylpyrrole Cap

Han Zhang [1,2,†], Qianqian Shen [3,†], Zhu Hu [2,†], Pei-Qian Wu [2], Yi Chen [3,4,*], Jin-Xin Zhao [2,4,*] and Jian-Min Yue [1,2,4,*]

[1] School of Chinese Materia Medica, Nanjing University of Chinese Medicine, 138 Xianlin Road, Nanjing, Jiangsu, 210023, China; zhanghan@simm.ac.cn (H.Z.)
[2] Ethnomedicine and Biofunctional Molecule Research Center, State Key Laboratory of Drug Research, Shanghai Institute of Materia Medica, Chinese Academy of Sciences, 555 Zuchongzhi Road, Shanghai 201203, China; huzhu@simm.ac.cn (Z.H.); wupeiqian@simm.ac.cn (P.-Q. W.)
[3] Division of Antitumor Pharmacology, State Key Laboratory of Chemical Biology, Shanghai Institute of Materia Medica, Chinese Academy of Sciences, 501 Haike Road, Shanghai 201203, China; shenqianqian@simm.ac.cn (Q.S.)
[4] Shandong Laboratory of Yantai Drug Discovery, Bohai Rim Advanced Research Institute for Drug Discovery, 198 East Binhai Road, Yantai, Shandong 264117, China
* Correspondence: ychen@simm.ac.cn (Y. C.), jxzhao@simm.ac.cn (J.-X. Z.), jmyue@simm.ac.cn (J.-M. Y.)
† The authors contributed equally to this paper.

Table of Contents

Figure S1. Physicochemical properties, lipophilicity, water solubility, pharmacokinetics, drug-likeness, and medicinal chemistry of 20 and chidamide predicted using SwissADME.... 6

Figure S2. HRESIMS spectrum of compound 3a. 6

Figure S3. ^{1}H NMR spectrum of compound 3a. 7

Figure S4. ^{13}C NMR spectrum of compound 3a. 7

Figure S5. HRESIMS spectrum of compound 3b. 8

Figure S6. ^{1}H NMR spectrum of compound 3b. 8

Figure S7. ^{13}C NMR spectrum of compound 3b. 9

Figure S8. HRESIMS spectrum of compound 3c. 9

Figure S9. ^{1}H NMR spectrum of compound 3c. 10

Figure S10. ^{13}C NMR spectrum of compound 3c. 10

Figure S11. HRESIMS spectrum of compound 3d. 11

Figure S12. ^{1}H NMR spectrum of compound 3d. 11

Figure S13. ^{13}C NMR spectrum of compound 3d. 12

Figure S14. HRESIMS spectrum of compound 3e. 12

Figure S15. ^{1}H NMR spectrum of compound 3e. 13

Figure S16. ^{13}C NMR spectrum of compound 3e. 13

Figure S17. HRESIMS spectrum of compound 5a. 14

Figure S18. ^{1}H NMR spectrum of compound 5a. 14

Figure S19. ^{13}C NMR spectrum of compound 5a. 15

Figure S20. HRESIMS spectrum of compound 5b. 15

Figure S21. ^{1}H NMR spectrum of compound 5b. 16

Figure S22. ^{13}C NMR spectrum of compound 5b. 16

Figure S23. HRESIMS spectrum of compound 5c. 17

Figure S24. ^{1}H NMR spectrum of compound 5c. 17

Figure S25. ^{13}C NMR spectrum of compound 5c. 18

Figure S26. HRESIMS spectrum of compound 5d. 18

Figure S27. ^{1}H NMR spectrum of compound 5d. 19

Figure S28. ^{13}C NMR spectrum of compound 5d. 19

Figure S29. HRESIMS spectrum of compound 5e. 20

Figure S30. ^{1}H NMR spectrum of compound 5e. 20
Figure S31. ^{13}C NMR spectrum of compound 5e. 21
Figure S32. HRESIMS spectrum of compound 5f. 21
Figure S33. ^{1}H NMR spectrum of compound 5f. 22
Figure S34. ^{13}C NMR spectrum of compound 5f. 22
Figure S35. HRESIMS spectrum of compound 5g. 23
Figure S36. ^{1}H NMR spectrum of compound 5g. 23
Figure S37. ^{13}C NMR spectrum of compound 5g. 24
Figure S38. HRESIMS spectrum of compound 5h. 24
Figure S39. ^{1}H NMR spectrum of compound 5h. 25
Figure S40. ^{13}C NMR spectrum of compound 5h. 25
Figure S41. HRESIMS spectrum of compound 5i. 26
Figure S42. ^{1}H NMR spectrum of compound 5i. 26
Figure S43. ^{13}C NMR spectrum of compound 5i. 27
Figure S44. HRESIMS spectrum of compound 5j. 27
Figure S45. ^{1}H NMR spectrum of compound 5j. 28
Figure S46. ^{13}C NMR spectrum of compound 5j. 28
Figure S47. HRESIMS spectrum of compound 5k. 29
Figure S48. ^{1}H NMR spectrum of compound 5k. 29
Figure S49. ^{13}C NMR spectrum of compound 5k. 30
Figure S50. HRESIMS spectrum of compound 5l. 30
Figure S51. ^{1}H NMR spectrum of compound 5l. 31
Figure S52. ^{13}C NMR spectrum of compound 5l. 31
Figure S53. HRESIMS spectrum of compound 11. 32
Figure S54. ^{1}H NMR spectrum of compound 11. 32
Figure S55. ^{13}C NMR spectrum of compound 11. 33
Figure S56. HRESIMS spectrum of compound 14. 33
Figure S57. ^{1}H NMR spectrum of compound 14. 34
Figure S58. ^{13}C NMR spectrum of compound 14. 34
Figure S59. HRESIMS spectrum of compound 19a. 35
Figure S60. ^{1}H NMR spectrum of compound 19a. 35
Figure S61. ^{13}C NMR spectrum of compound 19a. 36

Figure S62. HRESIMS spectrum of compound 19b. 36
Figure S63. ^{1}H NMR spectrum of compound 19b. 37
Figure S64. ^{13}C NMR spectrum of compound 19b. 37
Figure S65. HRESIMS spectrum of compound 19c. 38
Figure S66. ^{1}H NMR spectrum of compound 19c. 38
Figure S67. ^{13}C NMR spectrum of compound 19c. 39
Figure S68. HRESIMS spectrum of compound 19d. 39
Figure S69. ^{1}H NMR spectrum of compound 19d. 40
Figure S70. ^{13}C NMR spectrum of compound 19d. 40
Figure S71. HRESIMS spectrum of compound 19e. 41
Figure S72. ^{1}H NMR spectrum of compound 19e. 41
Figure S73. ^{13}C NMR spectrum of compound 19e. 42
Figure S74. HRESIMS spectrum of compound 19f. 42
Figure S75. ^{1}H NMR spectrum of compound 19f. 43
Figure S76. ^{13}C NMR spectrum of compound 19f. 43
Figure S77. HRESIMS spectrum of compound 20. 44
Figure S78. ^{1}H NMR spectrum of compound 20. 44
Figure S79. ^{13}C NMR spectrum of compound 20. 45
Figure S80. The whole un-cropped images of the original western blots in Figure 3. 46
Figure S81. The STR identification results of the three cells were correct. 47
Table S1. General information about the cells used in the experiments. 48

20

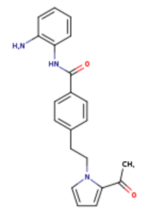

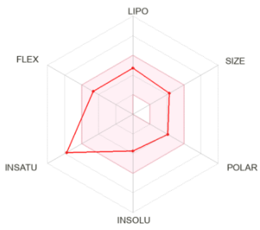

SMILES O=C(c1ccc(cc1)CCn1cccc1C(=O)C)Nc1ccccc1N

Physicochemical Properties	
Formula	C21H21N3O2
Molecular weight	347.41 g/mol
Num. heavy atoms	26
Num. arom. heavy atoms	17
Fraction Csp3	0.14
Num. rotatable bonds	7
Num. H-bond acceptors	2
Num. H-bond donors	2
Molar Refractivity	103.80
TPSA	77.12 Å²

Lipophilicity	
Log $P_{o/w}$ (iLOGP)	2.87
Log $P_{o/w}$ (XLOGP3)	2.72
Log $P_{o/w}$ (WLOGP)	3.59
Log $P_{o/w}$ (MLOGP)	2.00
Log $P_{o/w}$ (SILICOS-IT)	3.14
Consensus Log $P_{o/w}$	2.86

Water Solubility	
Log *S* (ESOL)	-3.73
Solubility	6.48e-02 mg/ml ; 1.86e-04 mol/l
Class	Soluble
Log *S* (Ali)	-3.99
Solubility	3.53e-02 mg/ml ; 1.02e-04 mol/l
Class	Soluble
Log *S* (SILICOS-IT)	-6.57
Solubility	9.44e-05 mg/ml ; 2.72e-07 mol/l
Class	Poorly soluble

Pharmacokinetics	
GI absorption	High
BBB permeant	Yes
P-gp substrate	Yes
CYP1A2 inhibitor	Yes
CYP2C19 inhibitor	Yes
CYP2C9 inhibitor	Yes
CYP2D6 inhibitor	Yes
CYP3A4 inhibitor	Yes
Log K_p (skin permeation)	-6.49 cm/s

Druglikeness	
Lipinski	Yes; 0 violation
Ghose	Yes
Veber	Yes
Egan	Yes
Muegge	Yes
Bioavailability Score	0.55

Medicinal Chemistry	
PAINS	0 alert
Brenk	1 alert: aniline
Leadlikeness	Yes
Synthetic accessibility	2.39

Chidamide

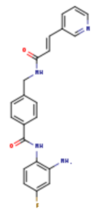

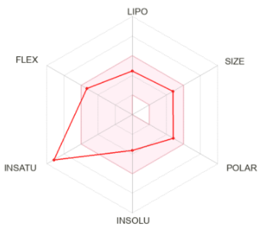

SMILES O=C(/C=C/c1cccnc1)NCc1ccc(cc1)C(=O)Nc1ccc(cc1N)F

Physicochemical Properties	
Formula	C22H19FN4O2
Molecular weight	390.41 g/mol
Num. heavy atoms	29
Num. arom. heavy atoms	18
Fraction Csp3	0.05
Num. rotatable bonds	8
Num. H-bond acceptors	4
Num. H-bond donors	3
Molar Refractivity	110.01
TPSA	97.11 Å²

Lipophilicity	
Log $P_{o/w}$ (iLOGP)	2.51
Log $P_{o/w}$ (XLOGP3)	2.30
Log $P_{o/w}$ (WLOGP)	3.36
Log $P_{o/w}$ (MLOGP)	2.08
Log $P_{o/w}$ (SILICOS-IT)	3.45
Consensus Log $P_{o/w}$	2.74

Water Solubility	
Log *S* (ESOL)	-3.64
Solubility	8.93e-02 mg/ml ; 2.29e-04 mol/l
Class	Soluble
Log *S* (Ali)	-3.98
Solubility	4.11e-02 mg/ml ; 1.05e-04 mol/l
Class	Soluble
Log *S* (SILICOS-IT)	-7.41
Solubility	1.54e-05 mg/ml ; 3.94e-08 mol/l
Class	Poorly soluble

Pharmacokinetics	
GI absorption	High
BBB permeant	No
P-gp substrate	Yes
CYP1A2 inhibitor	Yes
CYP2C19 inhibitor	Yes
CYP2C9 inhibitor	Yes
CYP2D6 inhibitor	Yes
CYP3A4 inhibitor	Yes
Log K_p (skin permeation)	-7.05 cm/s

Druglikeness	
Lipinski	Yes; 0 violation
Ghose	Yes
Veber	Yes
Egan	Yes
Muegge	Yes
Bioavailability Score	0.55

Medicinal Chemistry	
PAINS	0 alert
Brenk	2 alerts: aniline, michael_acceptor_1
Leadlikeness	No; 2 violations: MW>350, Rotors>7
Synthetic accessibility	2.84

Figure S1. Physicochemical properties, lipophilicity, water solubility, pharmacokinetics, drug-likeness, and medicinal chemistry of **20** and chidamide predicted using SwissADME.

**Data Filename**	N_ESIH202306765.d	**Sample Name**	D4-H39
**Sample ID**		**Position**	P1-B2
**Instrument Name**	Agilent 6520 Q-TOF	**Acq Method**	20160324_MS_ESIH_NEG_1min.m
**Acquired Time**	11/27/2023 1:34:26 PM	**DA Method**	ESI-HR-20231114.m
**Comment**	ESIH by fangsu		

User Spectra

**Fragmentor Voltage**	**Collision Energy**	**Ionization Mode**
165	0	ESI

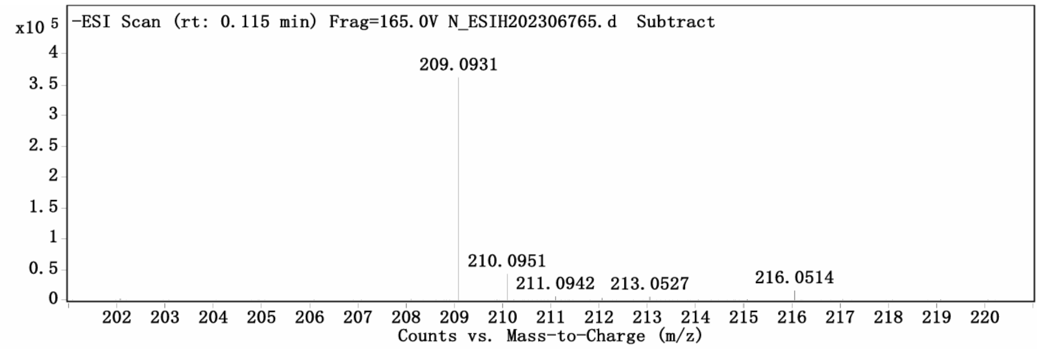

Formula Calculator Results

m/z	Calc m/z	Diff (mDa)	Diff (ppm)	Ion Formula	Ion
209.0931	209.0932	0.04	0.2	C10 H13 N2 O3	(M-H)-

--- End Of Report ---

Figure S2. HRESIMS spectrum of compound **3a**.

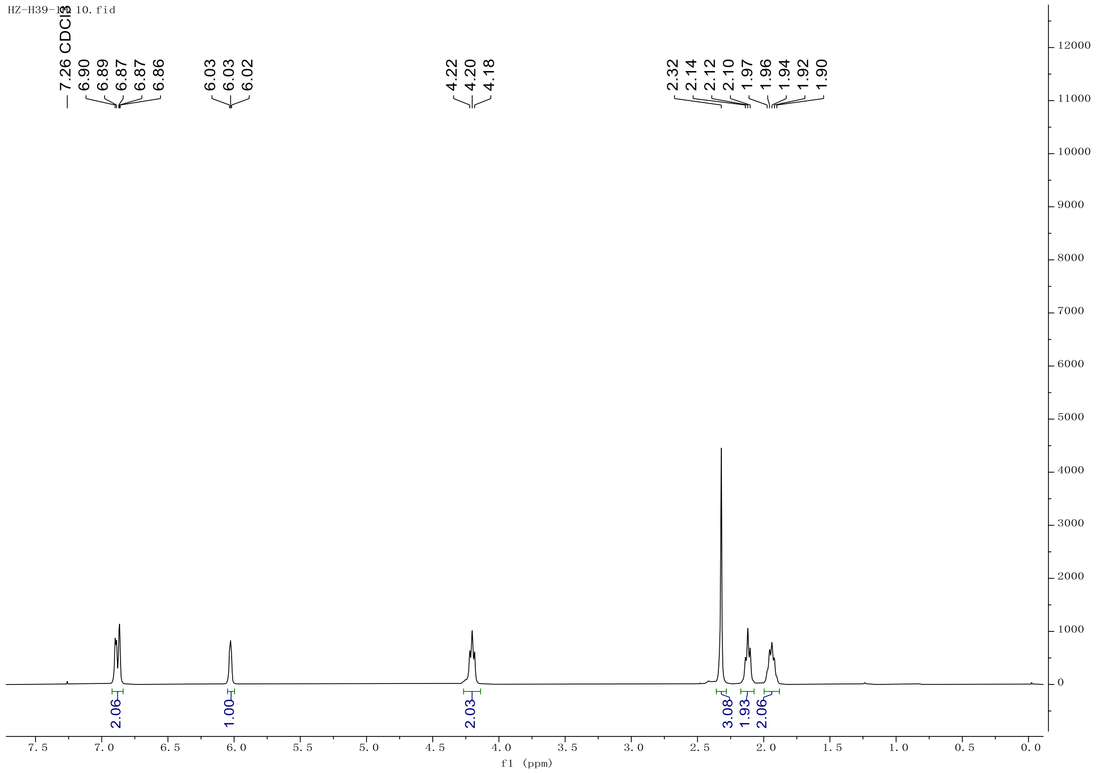

Figure S3. ^{1}H NMR spectrum of compound **3a**.

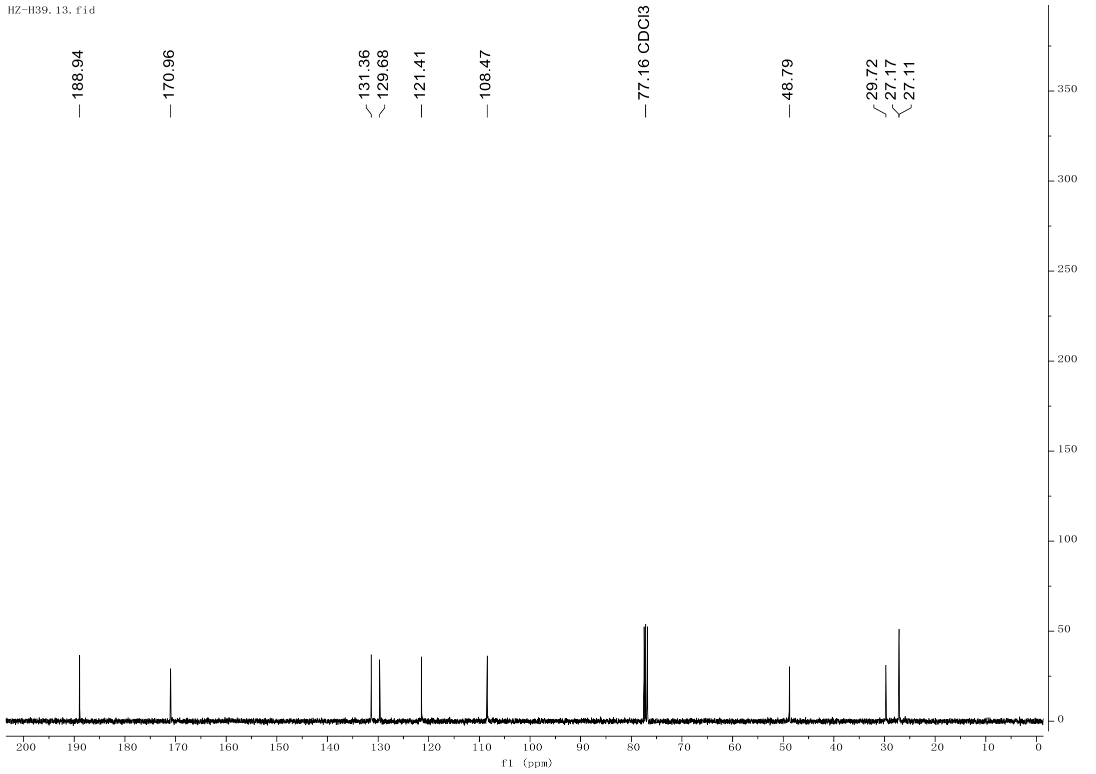

Figure S4. ^{13}C NMR spectrum of compound **3a**.

**Data Filename**	N_ESIH202306766.d	**Sample Name**	D4-H30
**Sample ID**		**Position**	P1-B3
**Instrument Name**	Agilent 6520 Q-TOF	**Acq Method**	20160324_MS_ESIH_NEG_1min.m
**Acquired Time**	11/27/2023 1:35:41 PM	**DA Method**	ESI-HR-20231114.m
**Comment**	ESIH by fangsu		

User Spectra

Fragmentor Voltage	Collision Energy	Ionization Mode
165	0	ESI

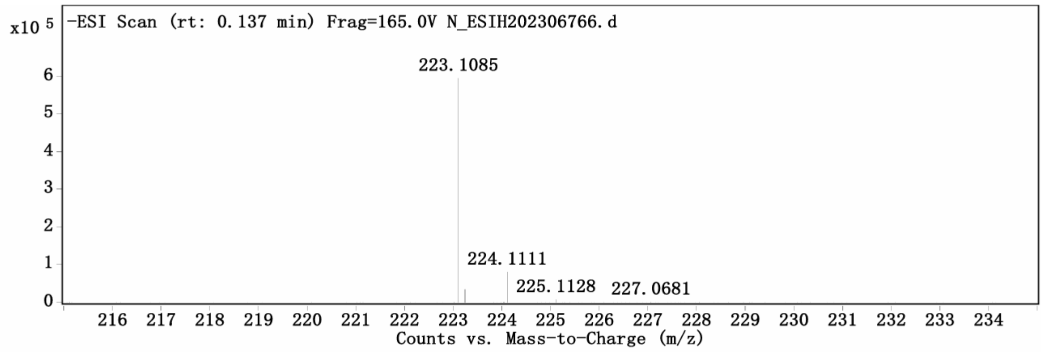

Formula Calculator Results

m/z	Calc m/z	Diff (mDa)	Diff (ppm)	Ion Formula	Ion
223.1085	223.1088	0.33	1.47	C11 H15 N2 O3	(M-H)-

--- End Of Report ---

Figure S5. HRESIMS spectrum of compound **3b**.

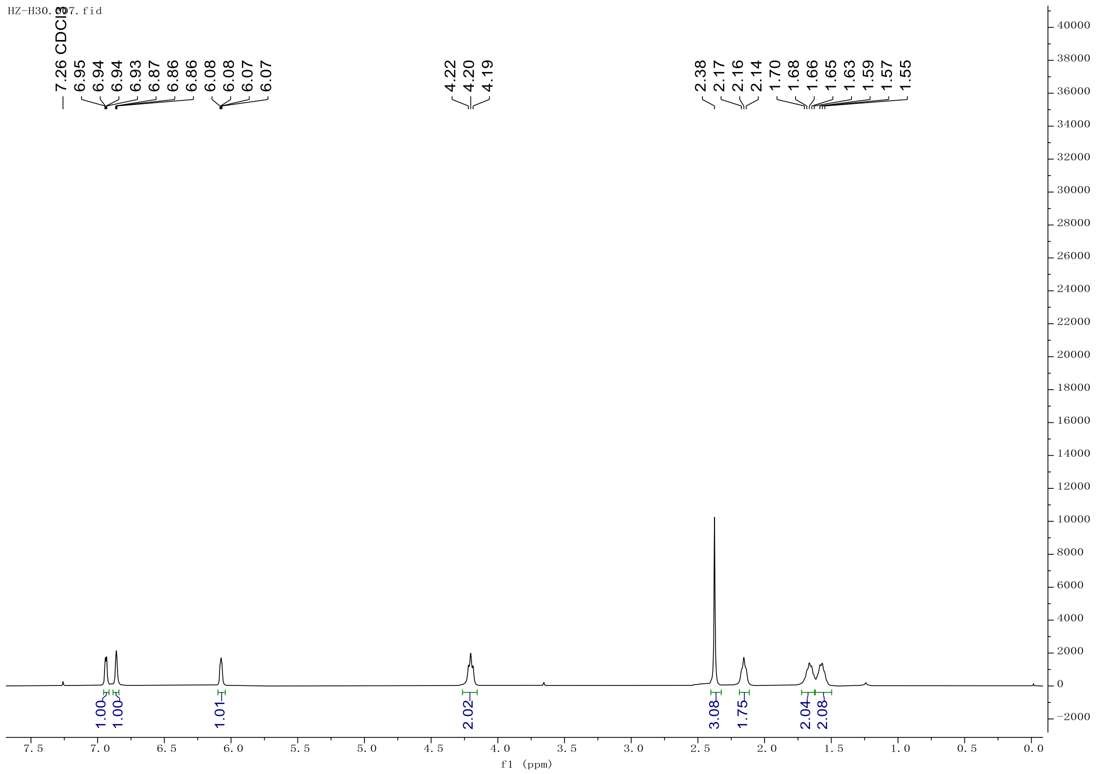

Figure S6. ^{1}H NMR spectrum of compound **3b**.

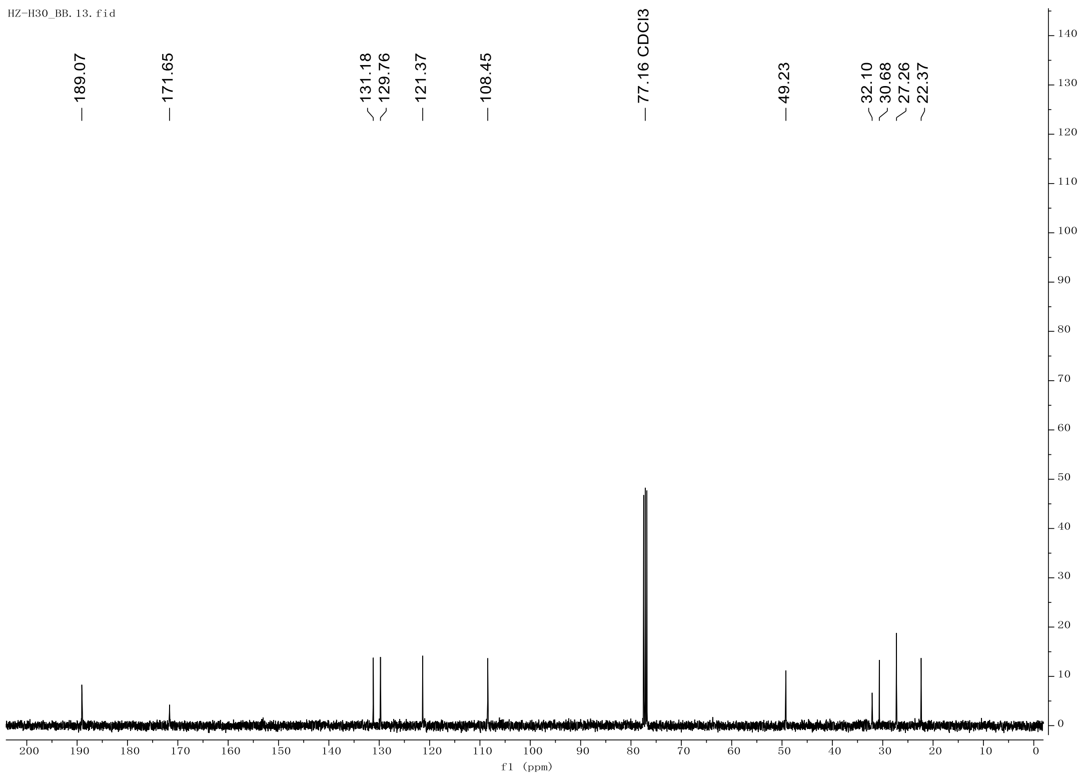

Figure S7. ^{13}C NMR spectrum of compound **3b**.

**Data Filename**	ESIH202400868.d	**Sample Name**	D4-D16
**Sample ID**		**Position**	P1-C1
**Instrument Name**	Agilent 6520 Q-TOF	**Acq Method**	20160322_MS_ESIH_POS_1min.m
**Acquired Time**	2/26/2024 4:17:44 PM	**DA Method**	ESI-HR-20231114.m
**Comment**	ESIH by fangsu		

User Spectra

Fragmentor Voltage	Collision Energy	Ionization Mode
170	0	ESI

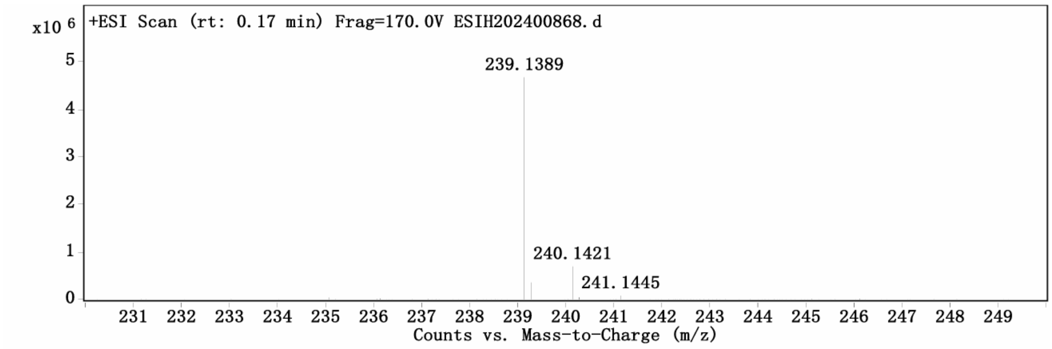

Formula Calculator Results

m/z	Calc m/z	Diff (mDa)	Diff (ppm)	Ion Formula	Ion
239.1389	239.139	0.09	0.36	C12 H19 N2 O3	(M+H)+

--- End Of Report ---

Figure S8. HRESIMS spectrum of compound **3c**.

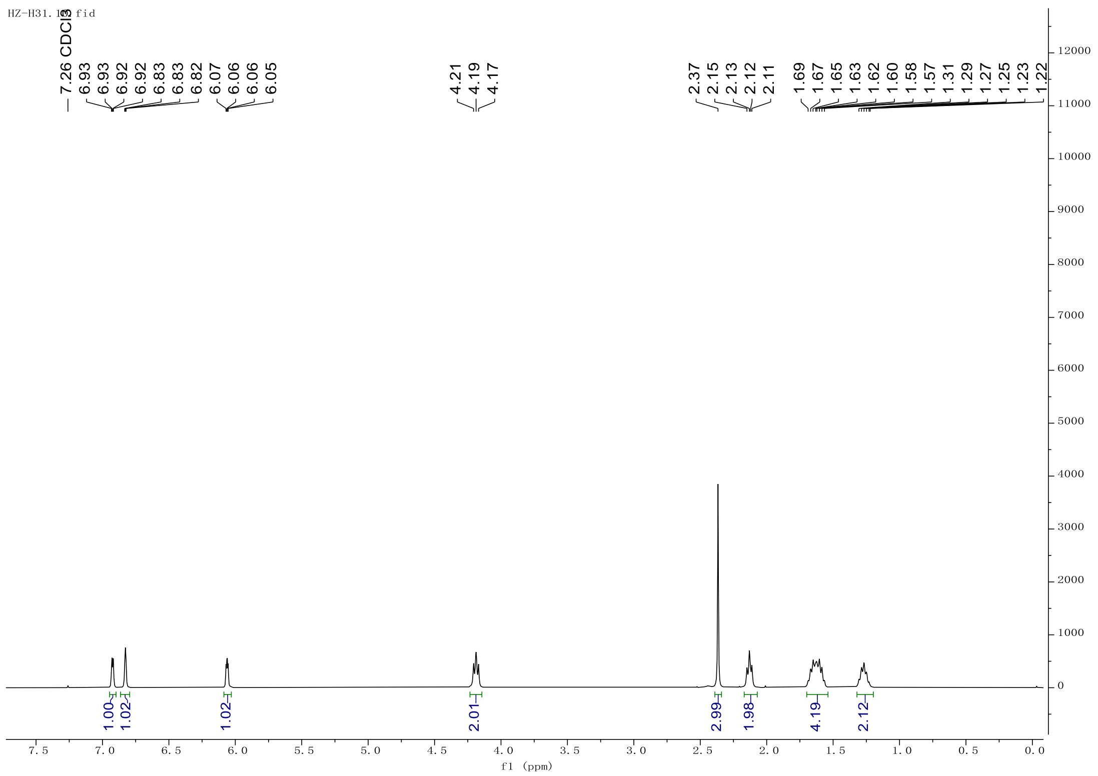

Figure S9. ^{1}H NMR spectrum of compound **3c**.

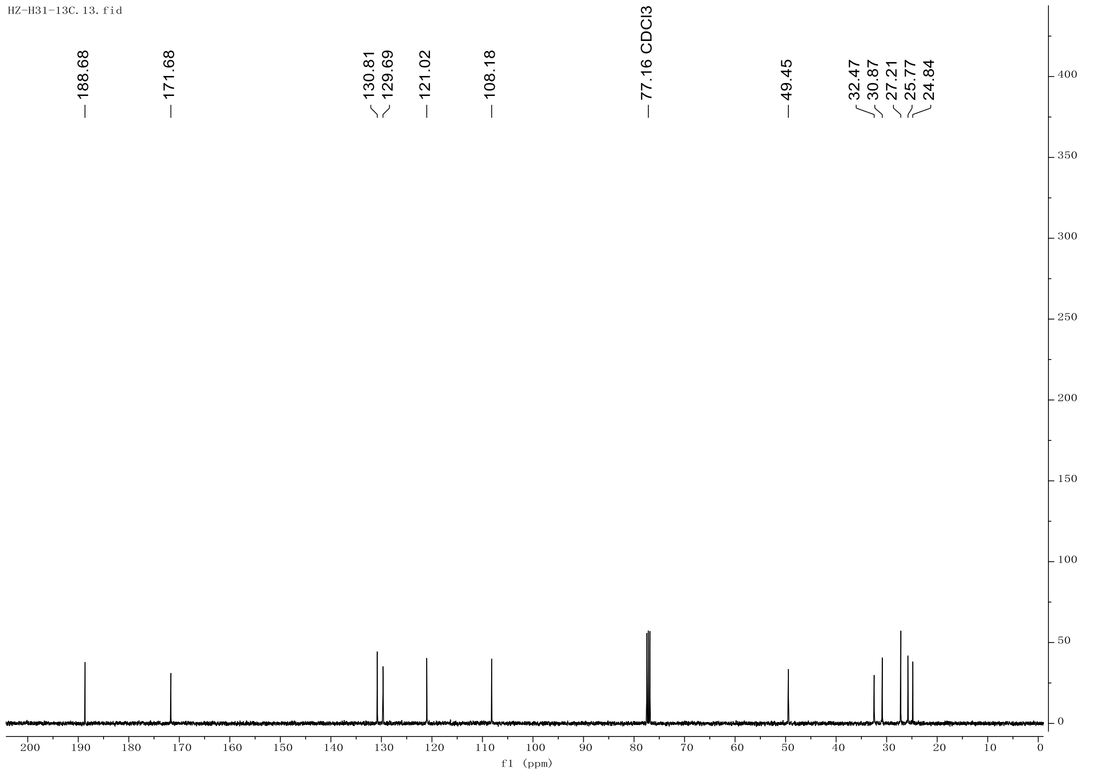

Figure S10. ^{13}C NMR spectrum of compound **3c**.

**Data Filename**	N_ESIH202306768.d	**Sample Name**	D4-H32
**Sample ID**		**Position**	P1-B5
**Instrument Name**	Agilent 6520 Q-TOF	**Acq Method**	20160324_MS_ESIH_NEG_1min.m
**Acquired Time**	11/27/2023 1:38:12 PM	**DA Method**	ESI-HR-20231114.m
**Comment**	ESIH by fangsu		

User Spectra

Fragmentor Voltage	Collision Energy	Ionization Mode
165	0	ESI

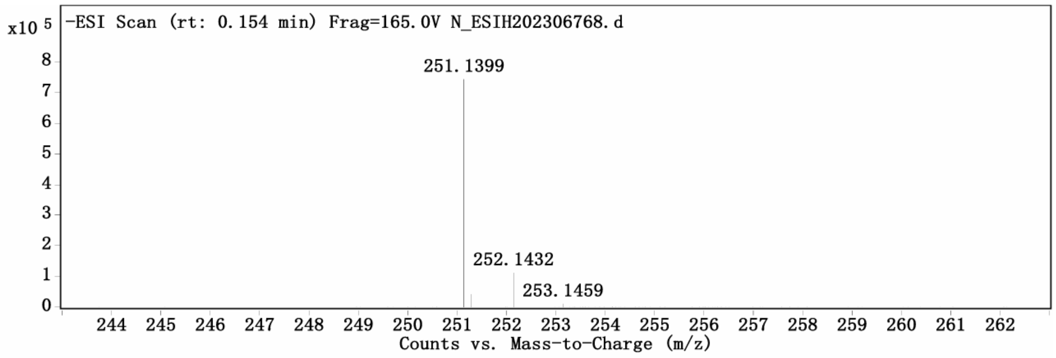

Formula Calculator Results

m/z	Calc m/z	Diff (mDa)	Diff (ppm)	Ion Formula	Ion
251.1399	251.1401	0.19	0.77	C13 H19 N2 O3	(M-H)-

--- End Of Report ---

Figure S11. HRESIMS spectrum of compound **3d**.

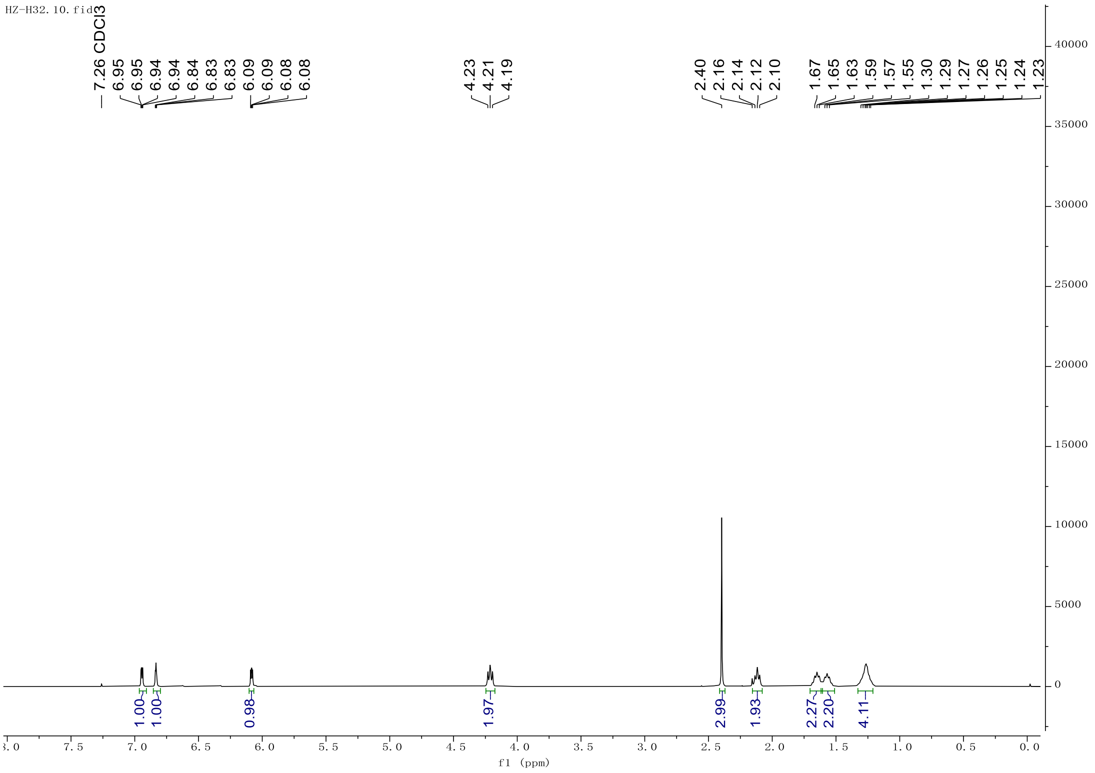

Figure S12. ^{1}H NMR spectrum of compound **3d**.

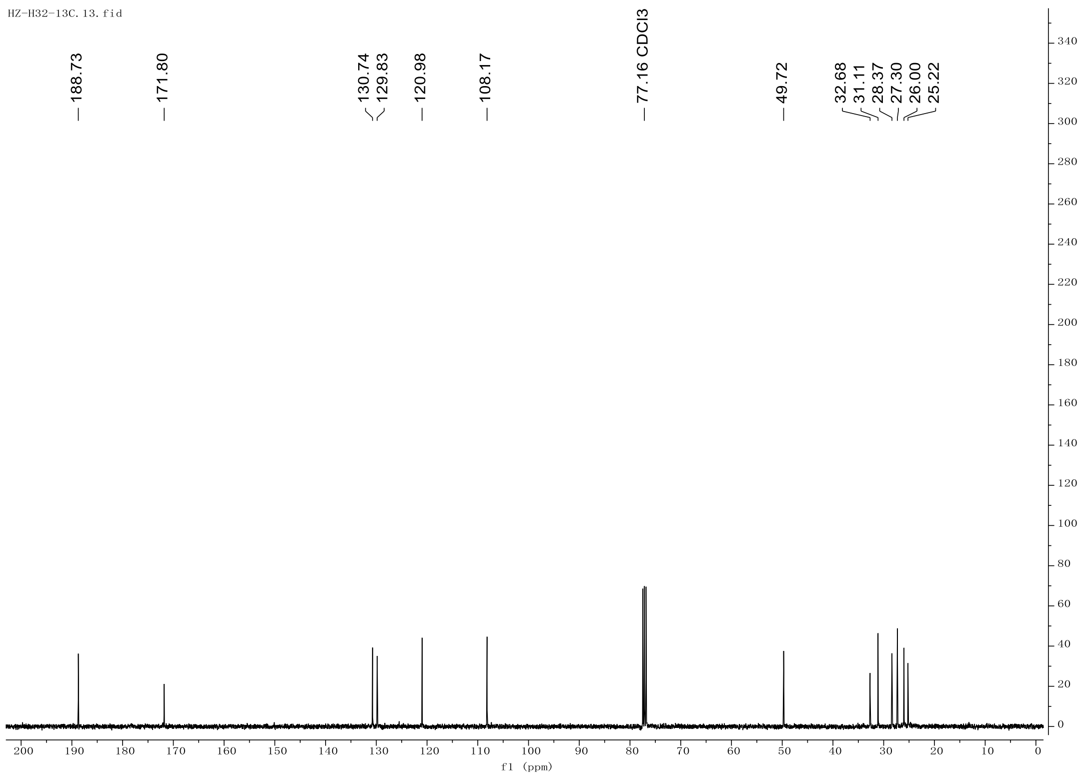

Figure S13. ^{13}C NMR spectrum of compound **3d**.

**Data Filename**	N_ESIH202306780.d	**Sample Name**	D4-D18
**Sample ID**		**Position**	P1-C8
**Instrument Name**	Agilent 6520 Q-TOF	**Acq Method**	20160324_MS_ESIH_NEG_1min.m
**Acquired Time**	11/27/2023 1:53:29 PM	**DA Method**	ESI-HR-20231114.m
**Comment**	ESIH by fangsu		

User Spectra

Fragmentor Voltage	Collision Energy	Ionization Mode
165	0	ESI

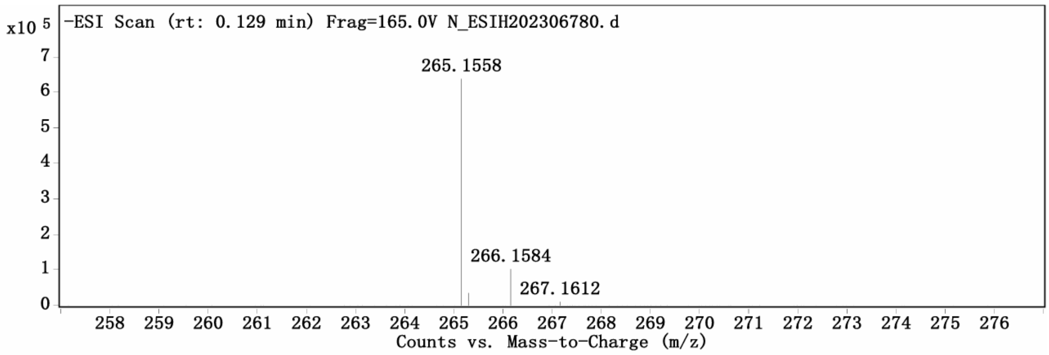

Formula Calculator Results

m/z	Calc m/z	Diff (mDa)	Diff (ppm)	Ion Formula	Ion
265.1558	265.1558	-0.02	-0.08	C14 H21 N2 O3	(M-H)-

--- End Of Report ---

Figure S14. HRESIMS spectrum of compound **3e**.

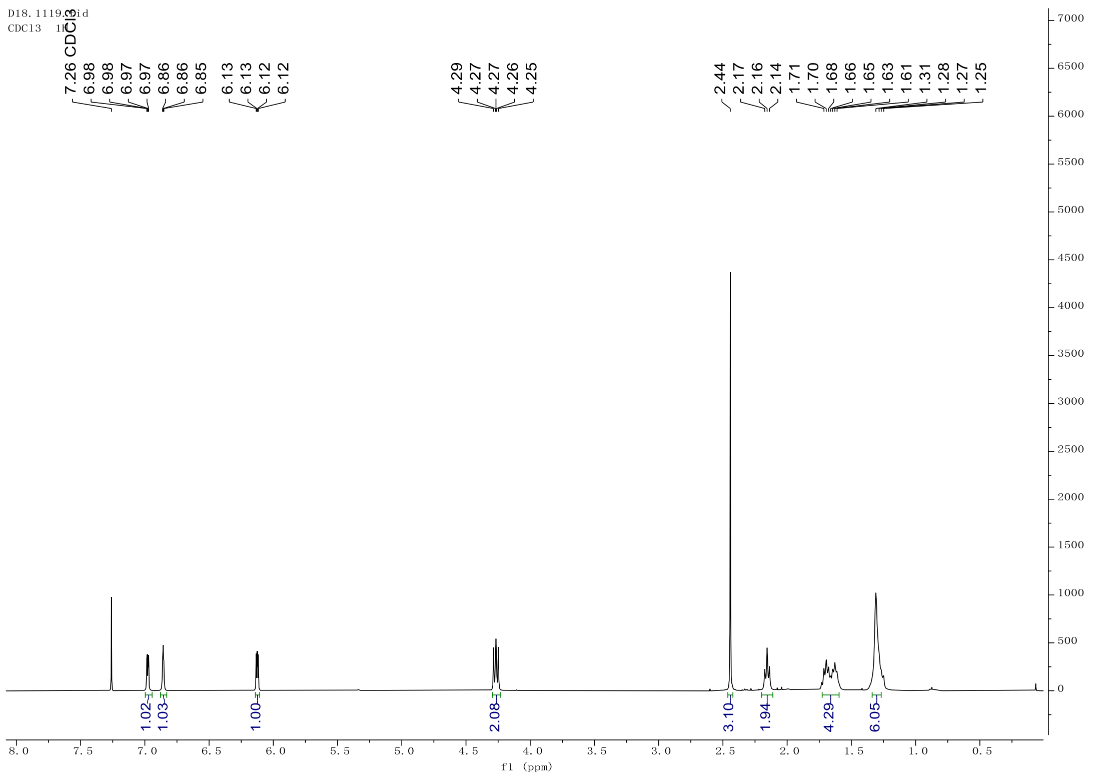

Figure S15. ^{1}H NMR spectrum of compound **3e**.

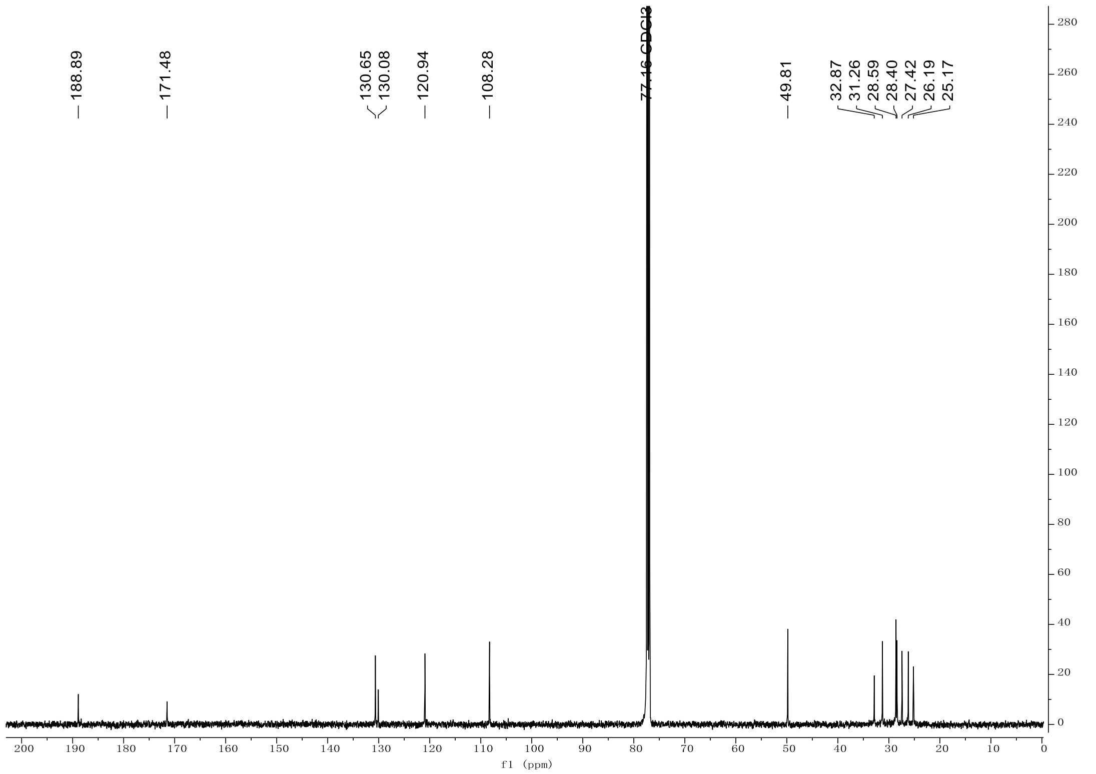

Figure S16. ^{13}C NMR spectrum of compound **3e**.

**Data Filename**	N_ESIH202306794.d	**Sample Name**	D4-D64
**Sample ID**		**Position**	P1-E4
**Instrument Name**	Agilent 6520 Q-TOF	**Acq Method**	20160324_MS_ESIH_NEG_1min.m
**Acquired Time**	11/27/2023 2:11:17 PM	**DA Method**	ESI-HR-20231114.m
**Comment**	ESIH by fangsu		

User Spectra

Fragmentor Voltage	Collision Energy	Ionization Mode
165	0	ESI

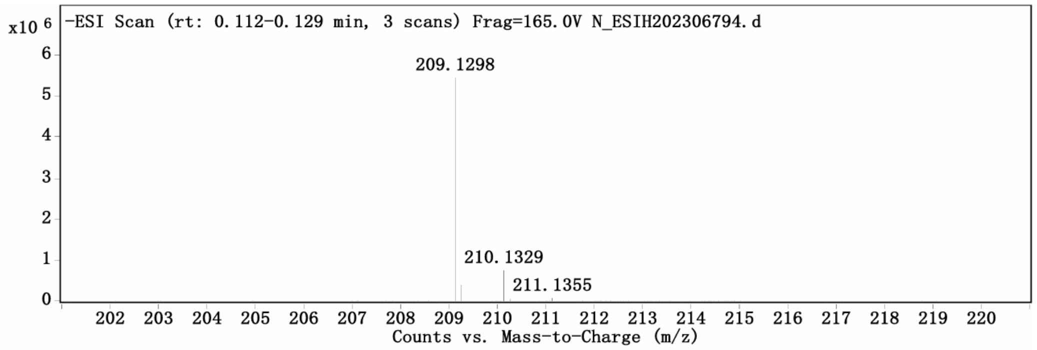

Formula Calculator Results

m/z	Calc m/z	Diff (mDa)	Diff (ppm)	Ion Formula	Ion
209.1298	209.1296	-0.28	-1.33	C11 H17 N2 O2	(M-H)-

--- End Of Report ---

Figure S17. HRESIMS spectrum of compound **5a**.

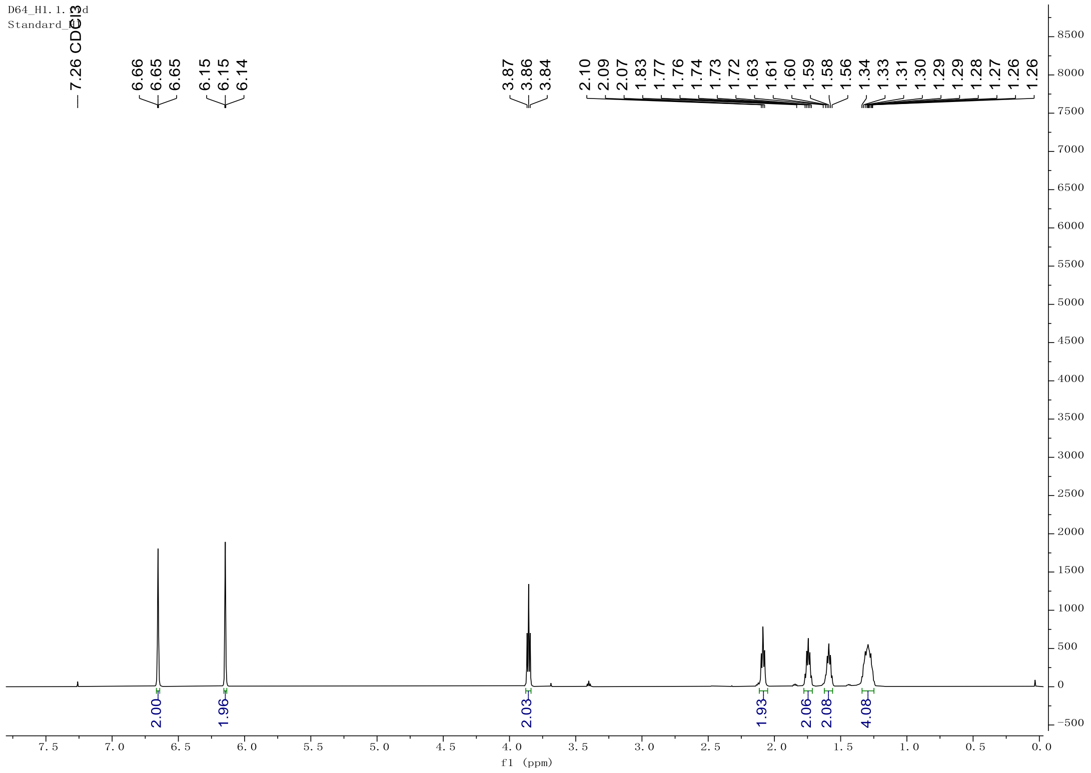

Figure S18. ^{1}H NMR spectrum of compound **5a**.

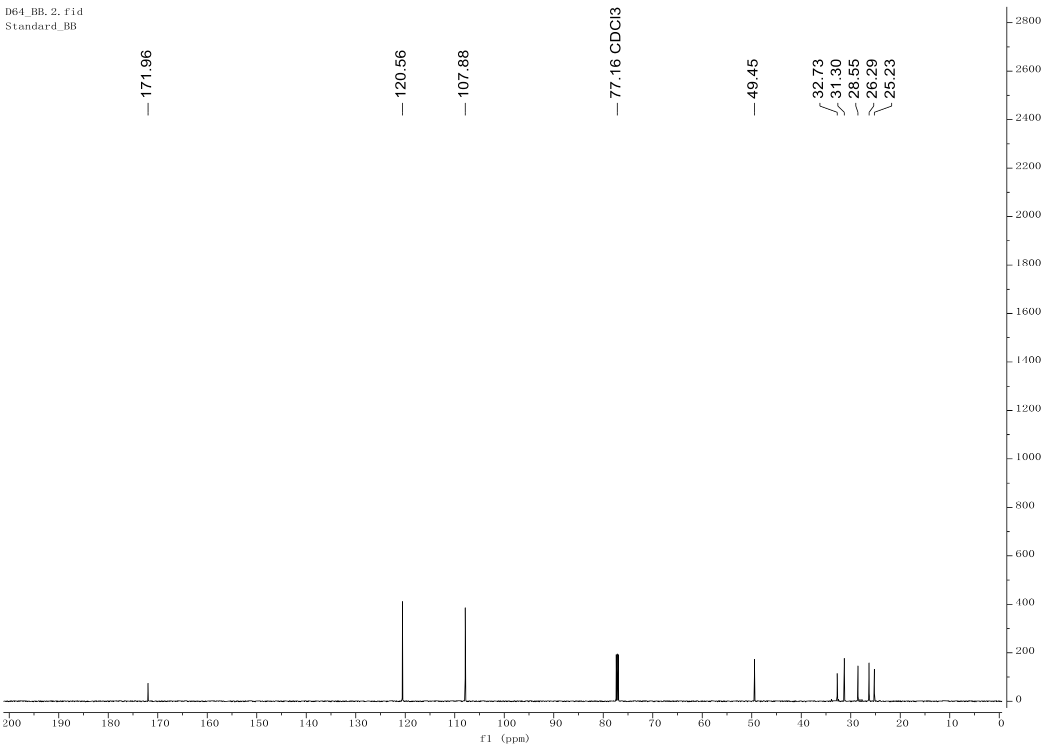

Figure S19. ^{13}C NMR spectrum of compound **5a**.

**Data Filename**	N_ESIH202306790.d	**Sample Name**	D4-D60
**Sample ID**		**Position**	P1-D9
**Instrument Name**	Agilent 6520 Q-TOF	**Acq Method**	20160324_MS_ESIH_NEG_1min.m
**Acquired Time**	11/27/2023 2:06:10 PM	**DA Method**	ESI-HR-20231114.m
**Comment**	ESIH by fangsu		

User Spectra

Fragmentor Voltage	Collision Energy	Ionization Mode
165	0	ESI

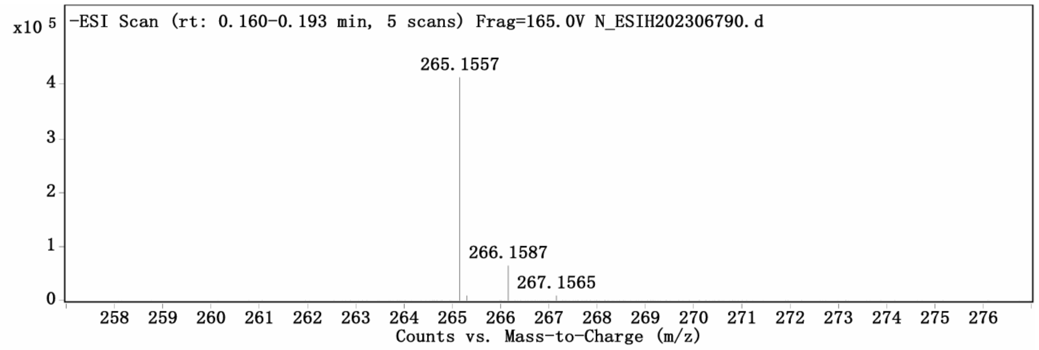

Formula Calculator Results

m/z	Calc m/z	Diff (mDa)	Diff (ppm)	Ion Formula	Ion
265.1557	265.1558	0.04	0.16	C14 H21 N2 O3	(M-H)-

--- End Of Report ---

Figure S20. HRESIMS spectrum of compound **5b**.

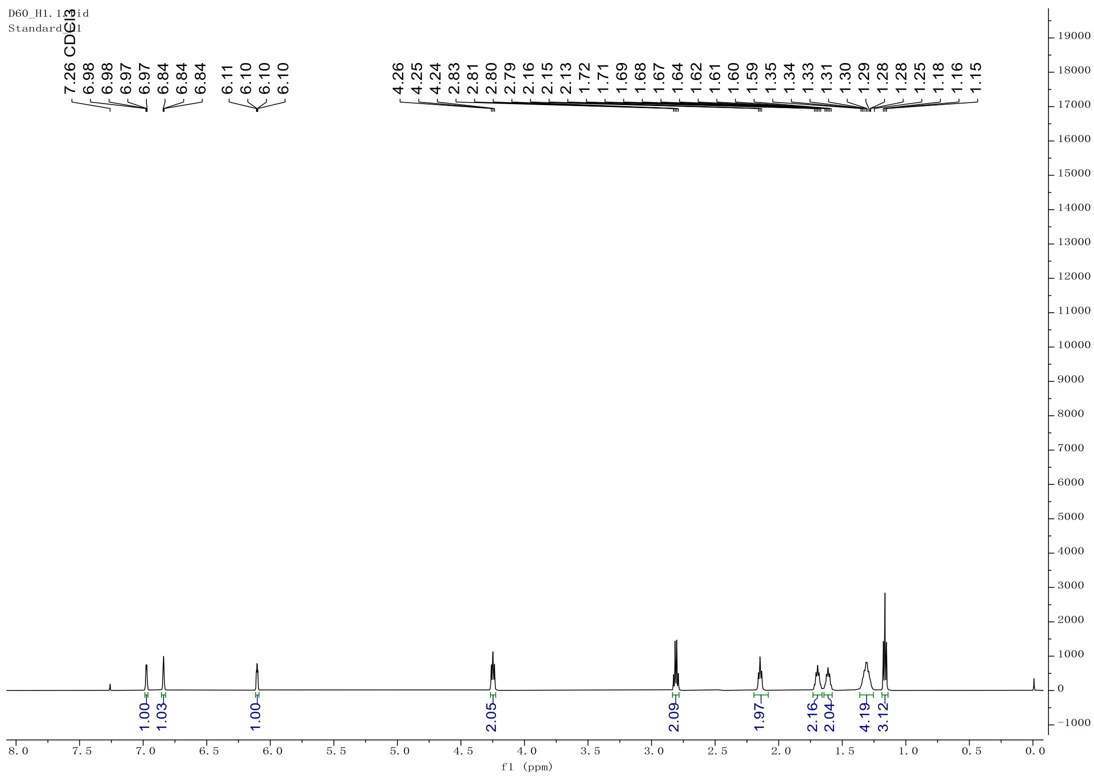

Figure S21. ^{1}H NMR spectrum of compound **5b**.

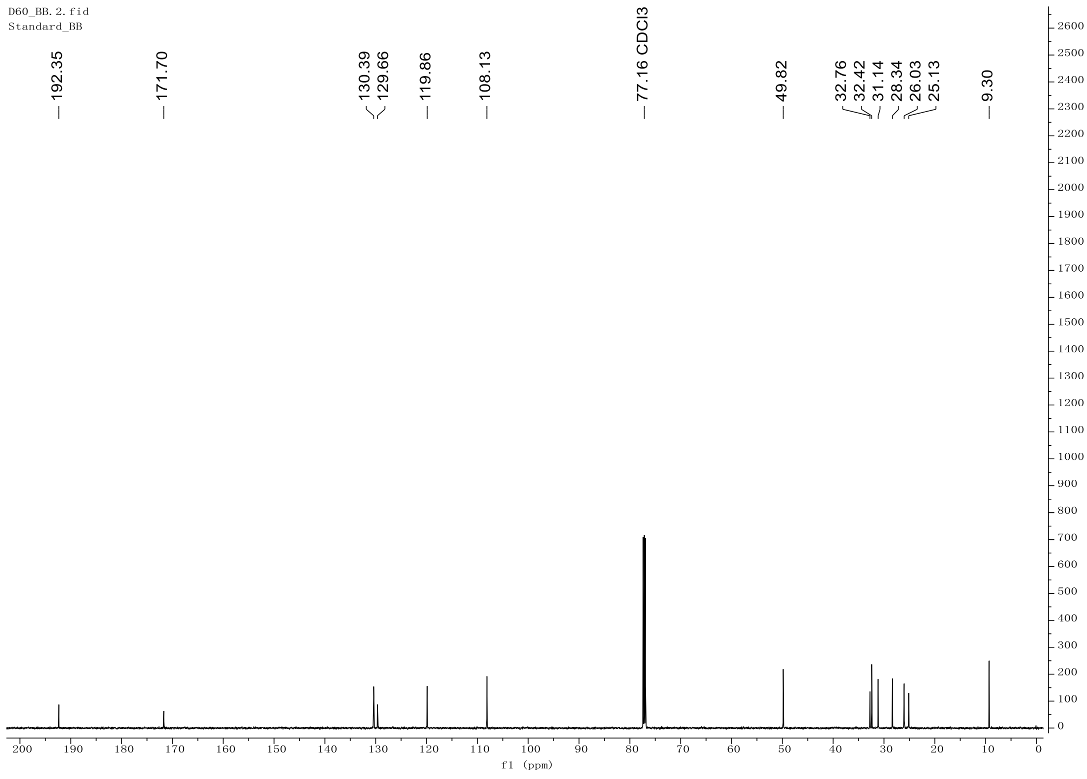

Figure S22. ^{13}C NMR spectrum of compound **5b**.

**Data Filename**	N_ESIH202306788.d	**Sample Name**	D4-D58
**Sample ID**		**Position**	P1-D7
**Instrument Name**	Agilent 6520 Q-TOF	**Acq Method**	20160324_MS_ESIH_NEG_1min.m
**Acquired Time**	11/27/2023 2:03:38 PM	**DA Method**	ESI-HR-20231114.m
**Comment**	ESIH by fangsu		

User Spectra

Fragmentor Voltage	Collision Energy	Ionization Mode
165	0	ESI

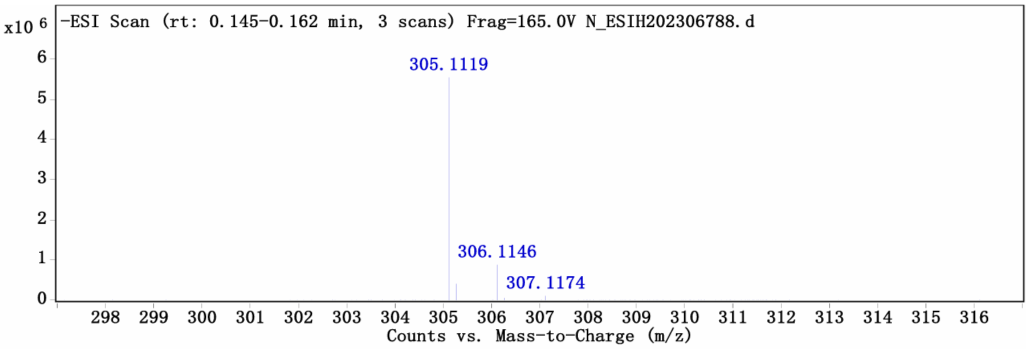

Formula Calculator Results

m/z	Calc m/z	Diff (mDa)	Diff (ppm)	Ion Formula	Ion
305.1119	305.1119	-0.05	-0.15	C13 H16 F3 N2 O3	(M-H)-

--- End Of Report ---

Figure S23. HRESIMS spectrum of compound **5c**.

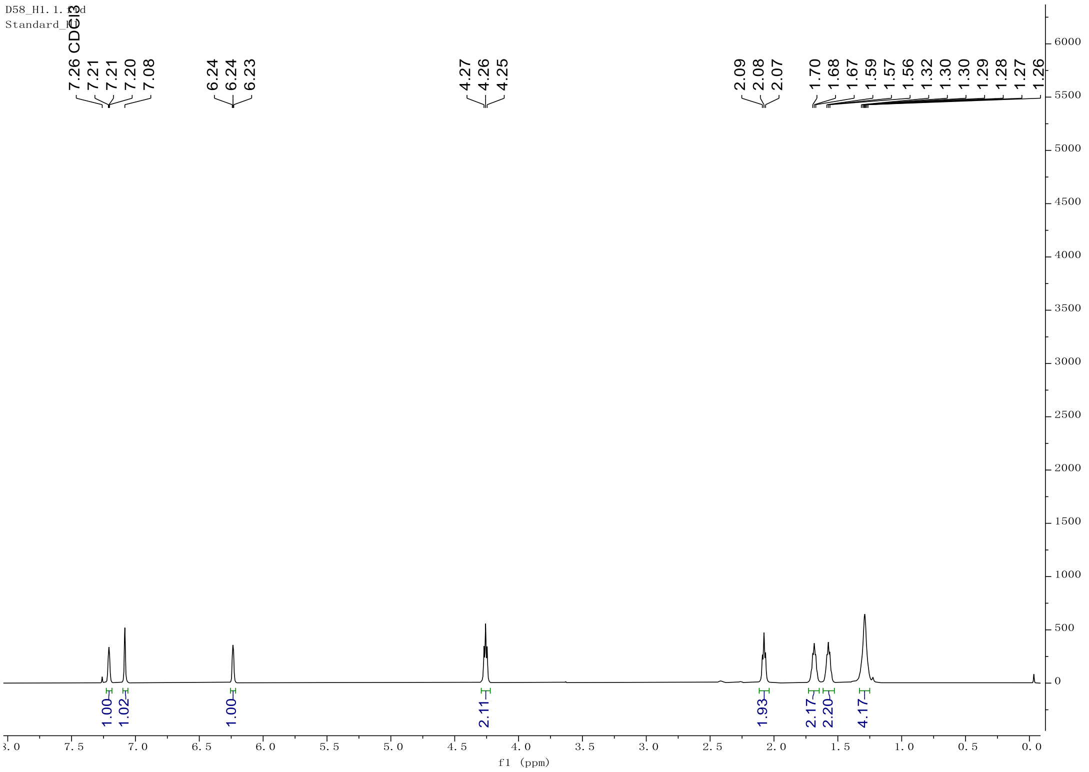

Figure S24. ^{1}H NMR spectrum of compound **5c**.

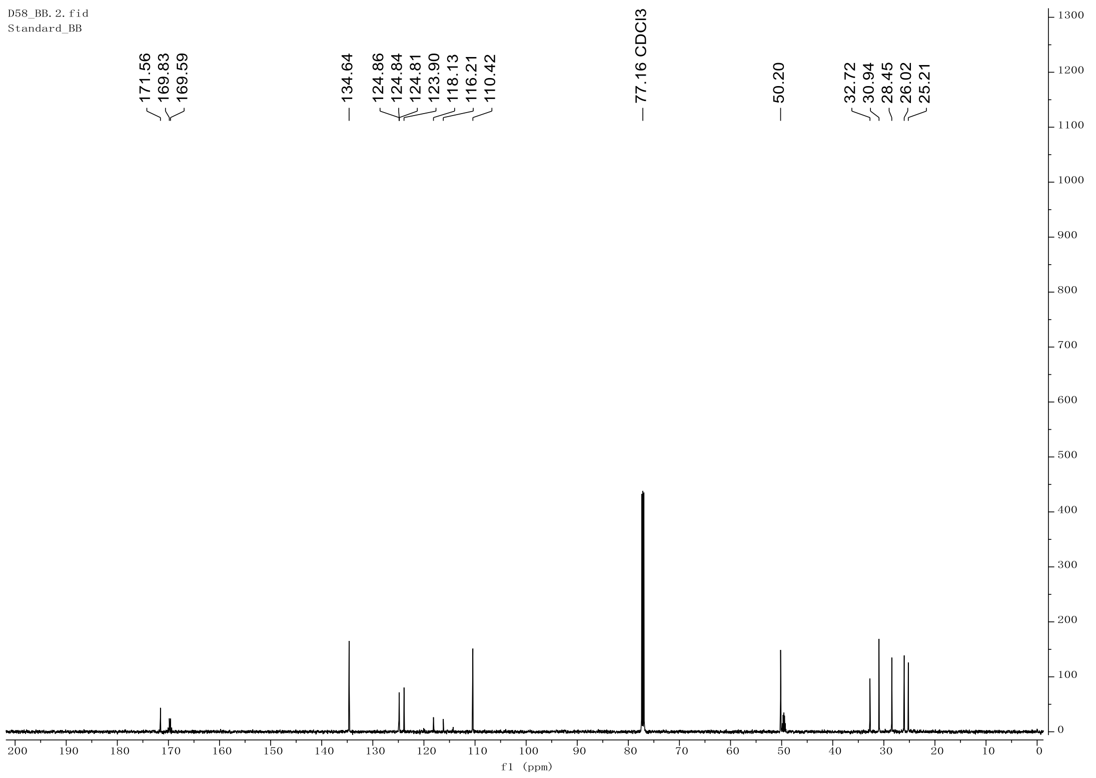

Figure S25. ^{13}C NMR spectrum of compound **5c**.

**Data Filename**	N_ESIH202306789.d	**Sample Name**	D4-D59
**Sample ID**		**Position**	P1-D8
**Instrument Name**	Agilent 6520 Q-TOF	**Acq Method**	20160324_MS_ESIH_NEG_1min.m
**Acquired Time**	11/27/2023 2:04:54 PM	**DA Method**	ESI-HR-20231114.m
**Comment**	ESIH by fangsu		

User Spectra

Fragmentor Voltage	Collision Energy	Ionization Mode
165	0	ESI

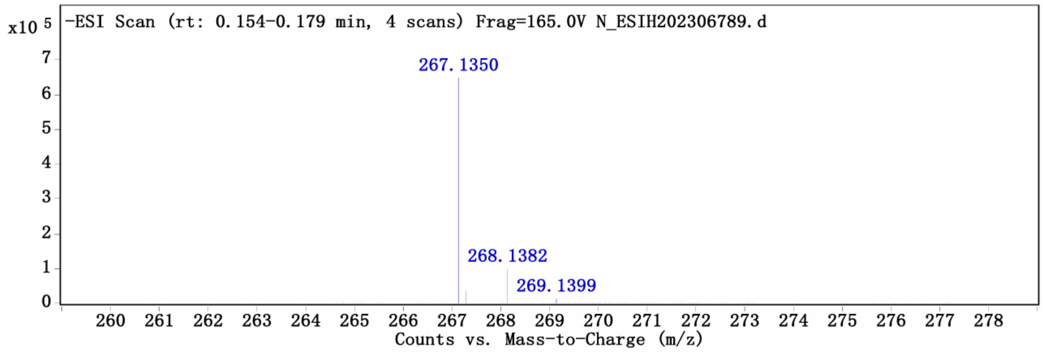

Formula Calculator Results

m/z	Calc m/z	Diff (mDa)	Diff (ppm)	Ion Formula	Ion
267.135	267.135	0.07	0.27	C13 H19 N2 O4	(M-H)-

--- End Of Report ---

Figure S26. HRESIMS spectrum of compound **5d**.

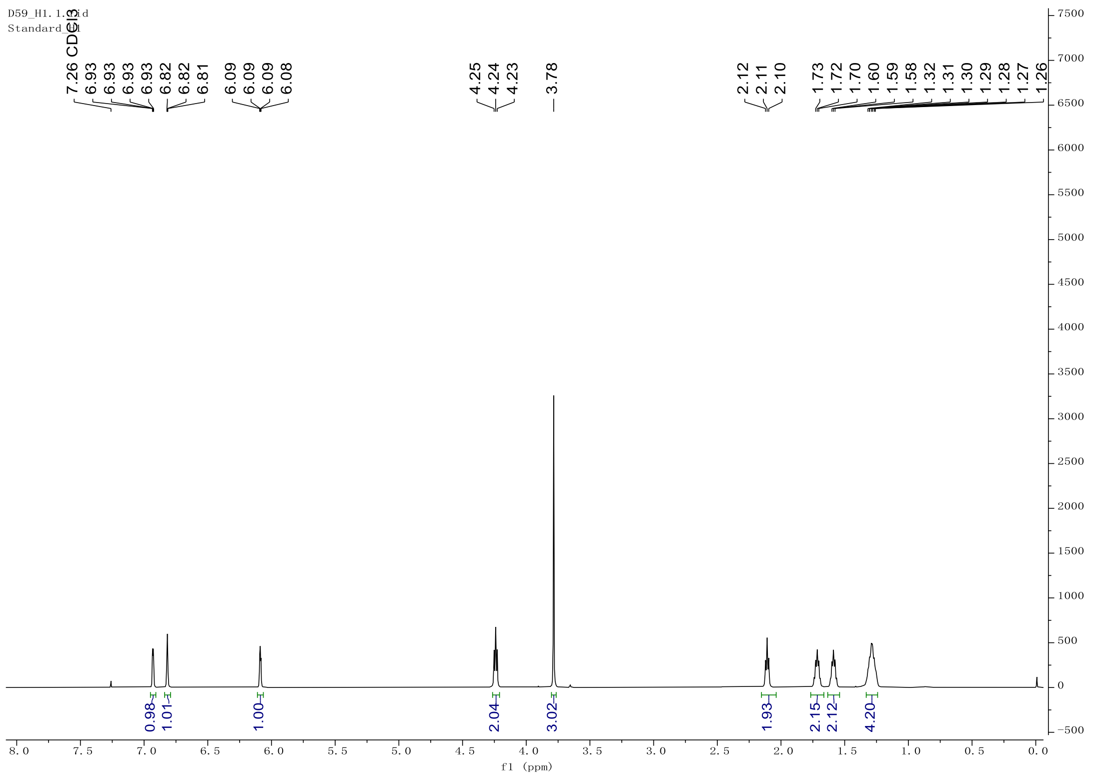

Figure S27. ^{1}H NMR spectrum of compound **5d**.

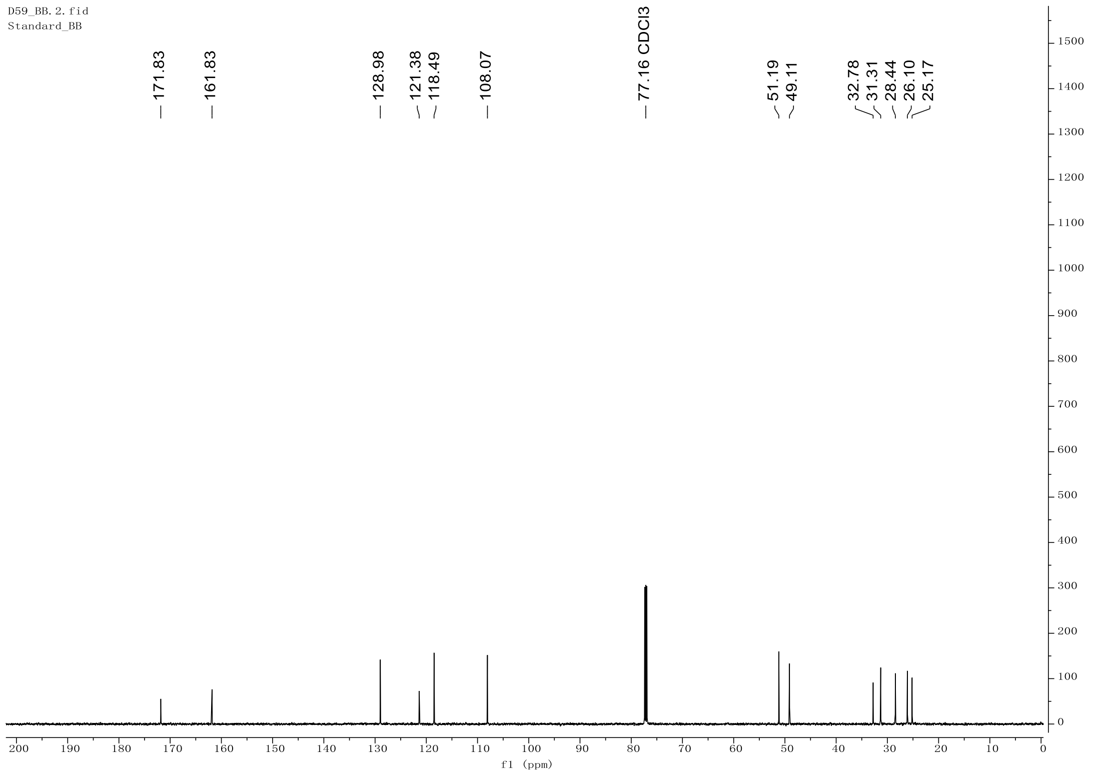

Figure S28. ^{13}C NMR spectrum of compound **5d**.

**Data Filename**	N_ESIH202306793.d	**Sample Name**	D4-D63
**Sample ID**		**Position**	P1-E3
**Instrument Name**	Agilent 6520 Q-TOF	**Acq Method**	20160324_MS_ESIH_NEG_1min.m
**Acquired Time**	11/27/2023 2:10:00 PM	**DA Method**	ESI-HR-20231114.m
**Comment**	ESIH by fangsu		

User Spectra

Fragmentor Voltage	Collision Energy	Ionization Mode
165	0	ESI

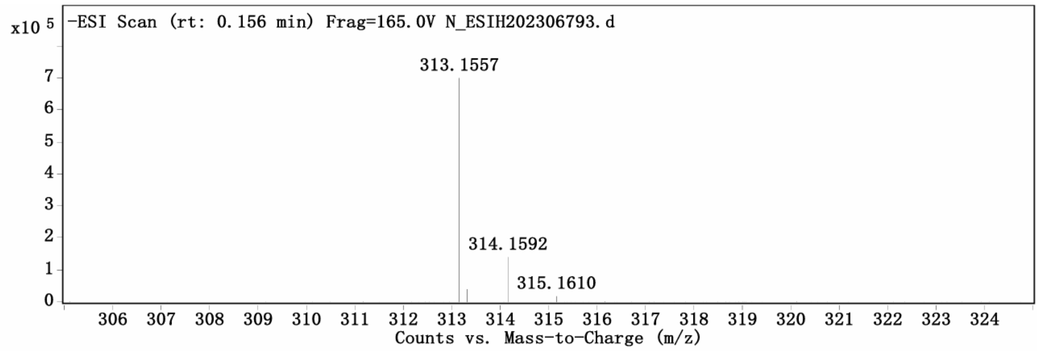

Formula Calculator Results

m/z	Calc m/z	Diff (mDa)	Diff (ppm)	Ion Formula	Ion
313.1557	313.1558	0.04	0.13	C18 H21 N2 O3	(M-H)-

--- End Of Report ---

Figure S29. HRESIMS spectrum of compound **5e**.

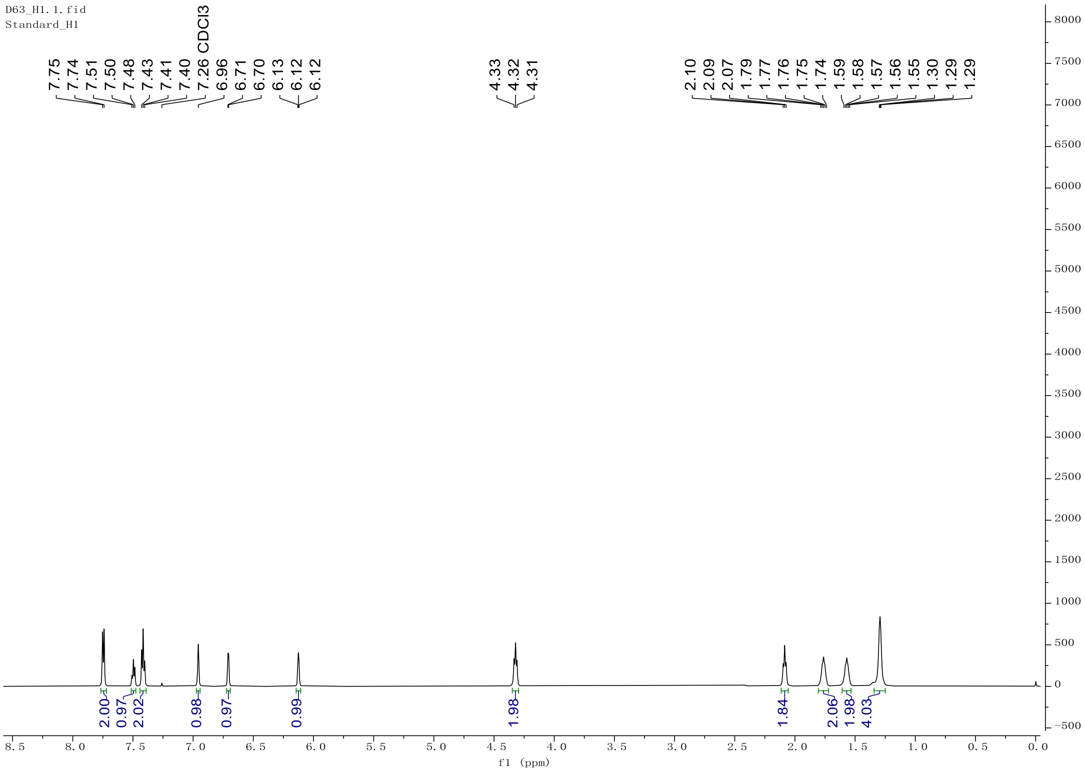

Figure S30. ^{1}H NMR spectrum of compound **5e**.

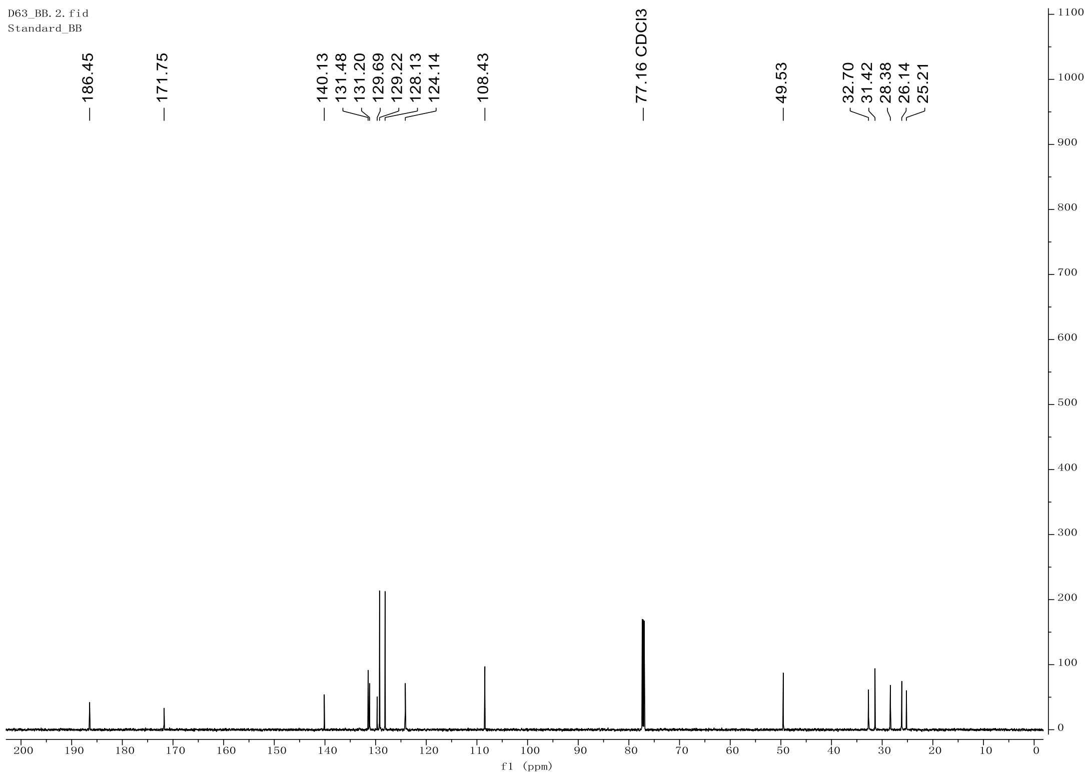

Figure S31. ^{13}C NMR spectrum of compound **5e**.

**Data Filename**	N_ESIH202306791.d	**Sample Name**	D4-D61
**Sample ID**		**Position**	P1-E1
**Instrument Name**	Agilent 6520 Q-TOF	**Acq Method**	20160324_MS_ESIH_NEG_1min.m
**Acquired Time**	11/27/2023 2:07:25 PM	**DA Method**	ESI-HR-20231114.m
**Comment**	ESIH by fangsu		

User Spectra

Fragmentor Voltage	Collision Energy	Ionization Mode
165	0	ESI

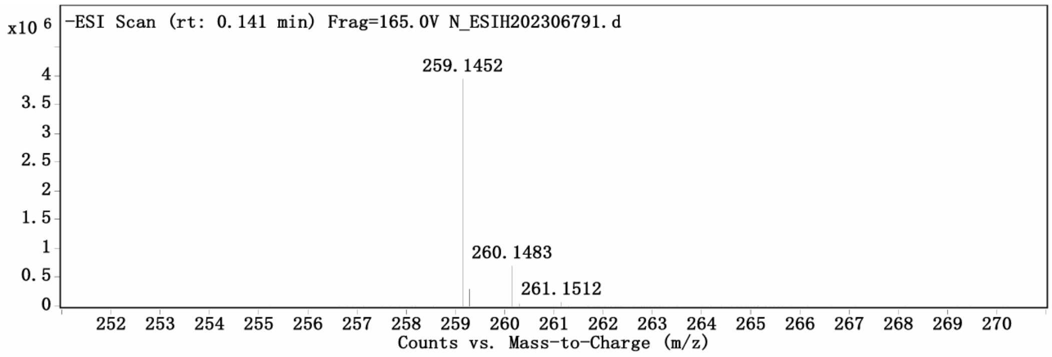

Formula Calculator Results

m/z	Calc m/z	Diff (mDa)	Diff (ppm)	Ion Formula	Ion
259.1452	259.1452	0.04	0.16	C15 H19 N2 O2	(M-H)-

--- End Of Report ---

Figure S32. HRESIMS spectrum of compound **5f**.

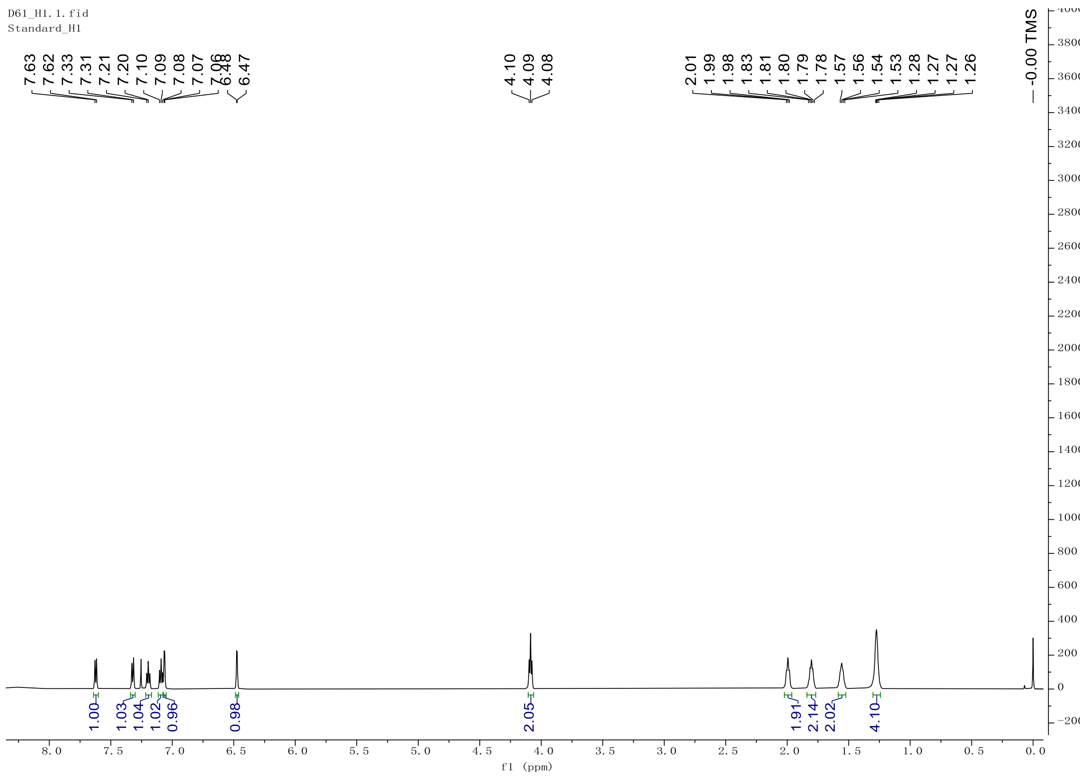

Figure S33. ^{1}H NMR spectrum of compound **5f**.

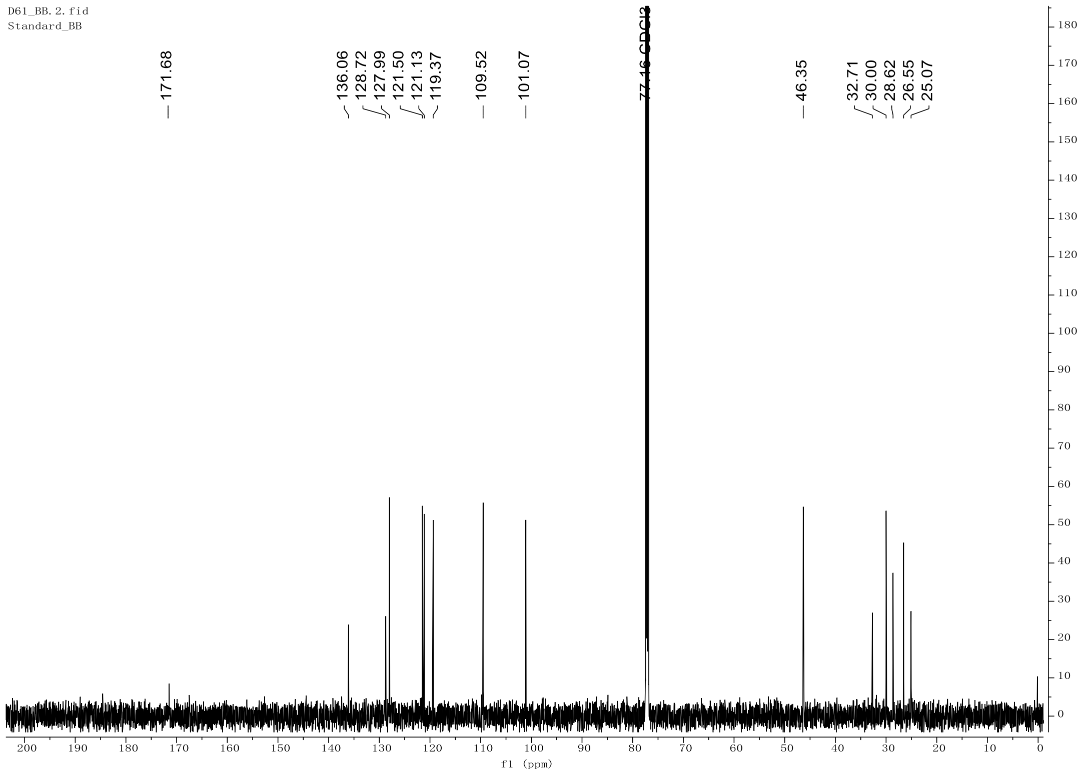

Figure S34. ^{13}C NMR spectrum of compound **5f**.

**Data Filename**	N_ESIH202306784.d	**Sample Name**	D4-D54
**Sample ID**		**Position**	P1-D3
**Instrument Name**	Agilent 6520 Q-TOF	**Acq Method**	20160324_MS_ESIH_NEG_1min.m
**Acquired Time**	11/27/2023 1:58:36 PM	**DA Method**	ESI-HR-20231114.m
**Comment**	ESIH by fangsu		

User Spectra

Fragmentor Voltage	Collision Energy	Ionization Mode
165	0	ESI

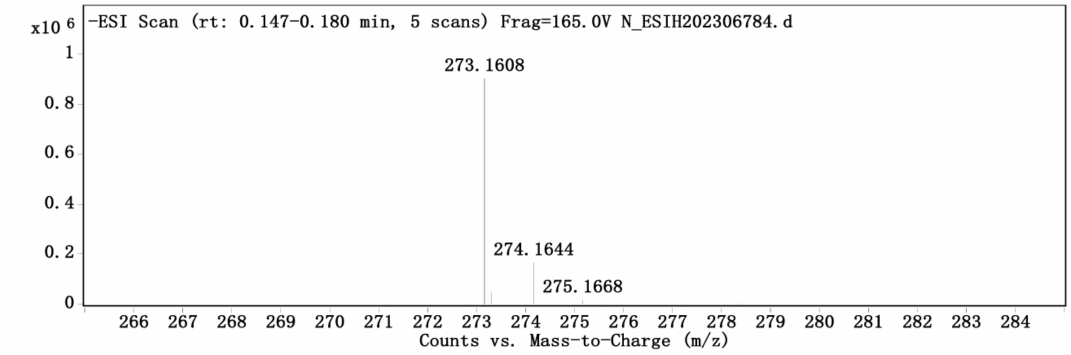

Formula Calculator Results

m/z	Calc m/z	Diff (mDa)	Diff (ppm)	Ion Formula	Ion
273.1608	273.1609	0.08	0.3	C16 H21 N2 O2	(M-H)-

--- End Of Report ---

Figure S35. HRESIMS spectrum of compound **5g**.

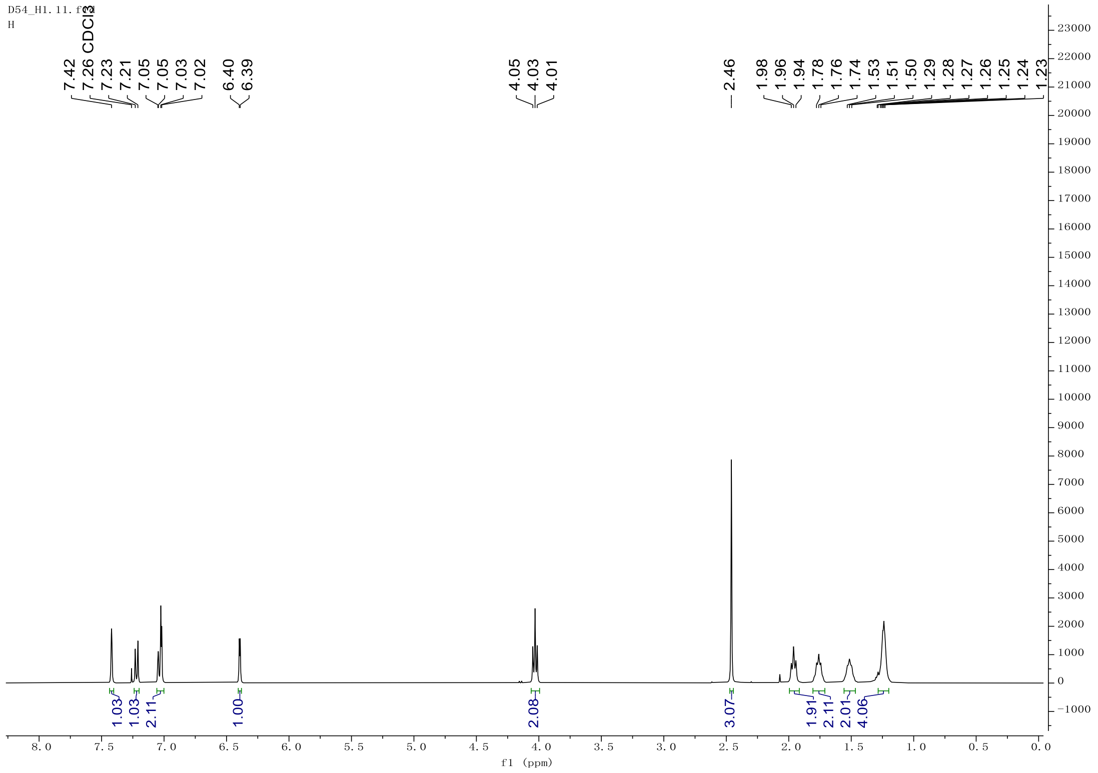

Figure S36. ^{1}H NMR spectrum of compound **5g**.

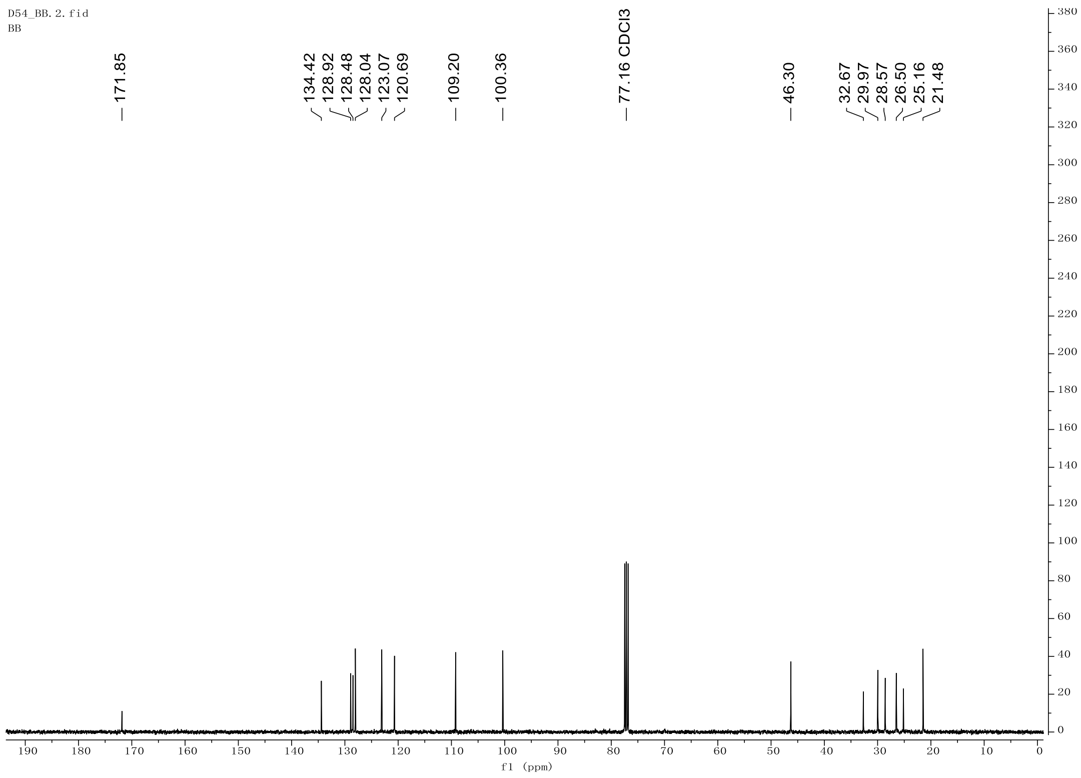

Figure S37. ^{13}C NMR spectrum of compound **5g**.

**Data Filename**	N_ESIH202306786.d	**Sample Name**	D4-D56
**Sample ID**		**Position**	P1-D5
**Instrument Name**	Agilent 6520 Q-TOF	**Acq Method**	20160324_MS_ESIH_NEG_1min.m
**Acquired Time**	11/27/2023 2:01:06 PM	**DA Method**	ESI-HR-20231114.m
**Comment**	ESIH by fangsu		

User Spectra

Fragmentor Voltage	Collision Energy	Ionization Mode
165	0	ESI

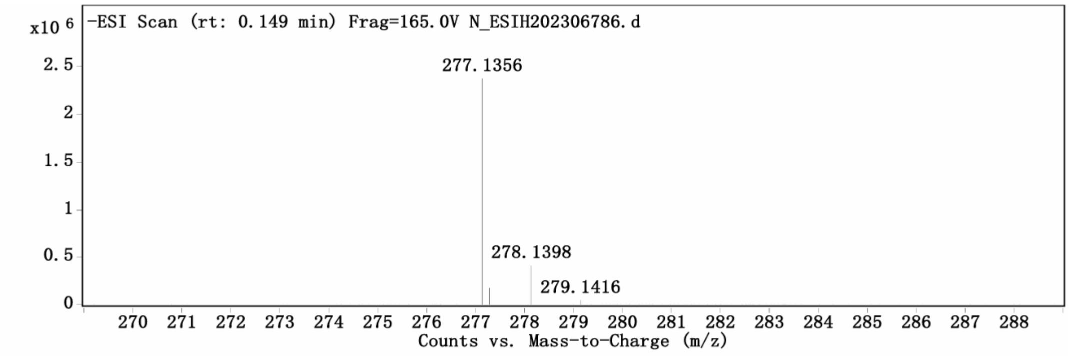

Formula Calculator Results

m/z	Calc m/z	Diff (mDa)	Diff (ppm)	Ion Formula	Ion
277.1356	277.1358	0.17	0.61	C15 H18 F N2 O2	(M-H)-

--- End Of Report ---

Figure S38. HRESIMS spectrum of compound **5h**.

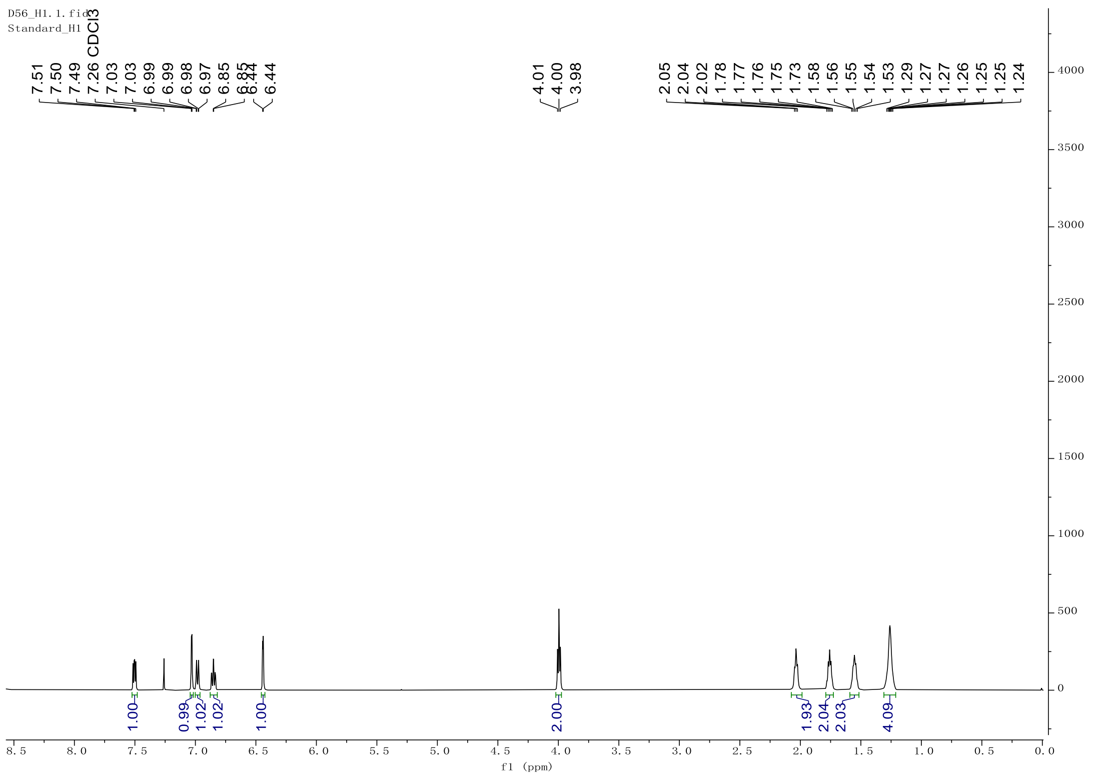

Figure S39. ^{1}H NMR spectrum of compound **5h**.

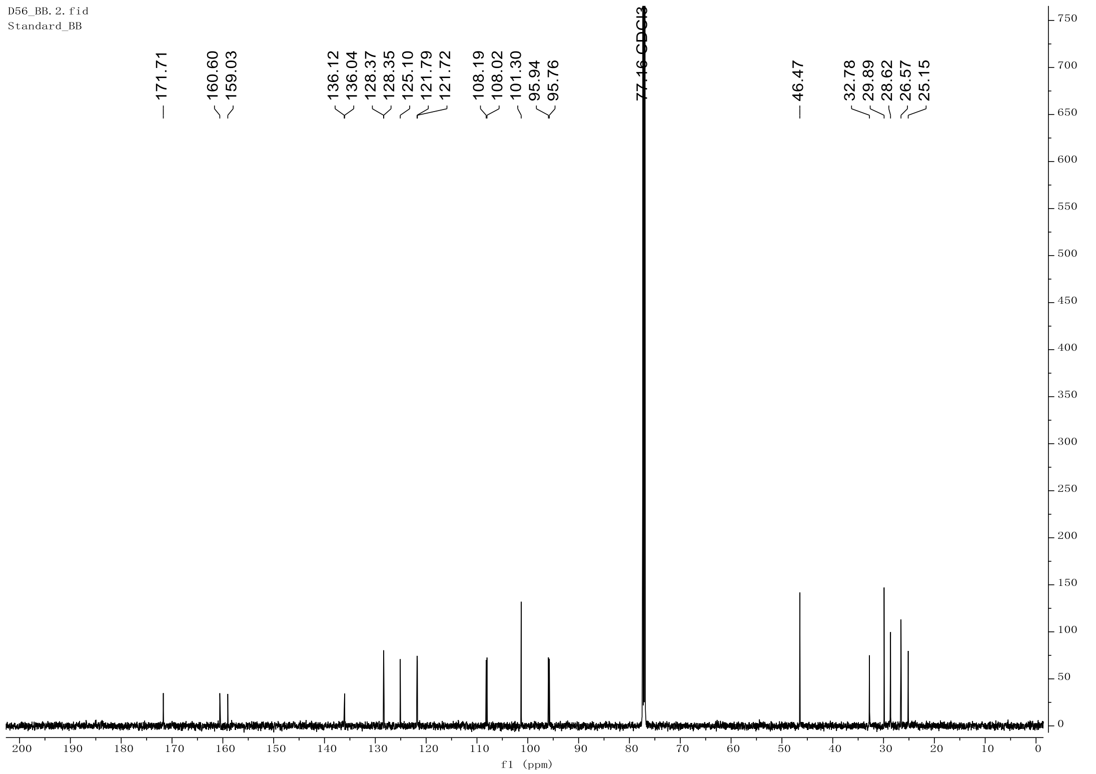

Figure S40. ^{13}C NMR spectrum of compound **5h**.

**Data Filename**	N_ESIH202306795.d	**Sample Name**	D4-D65
**Sample ID**		**Position**	P1-E5
**Instrument Name**	Agilent 6520 Q-TOF	**Acq Method**	20160324_MS_ESIH_NEG_1min.m
**Acquired Time**	11/27/2023 2:12:33 PM	**DA Method**	ESI-HR-20231114.m
**Comment**	ESIH by fangsu		

User Spectra

Fragmentor Voltage	Collision Energy	Ionization Mode
165	0	ESI

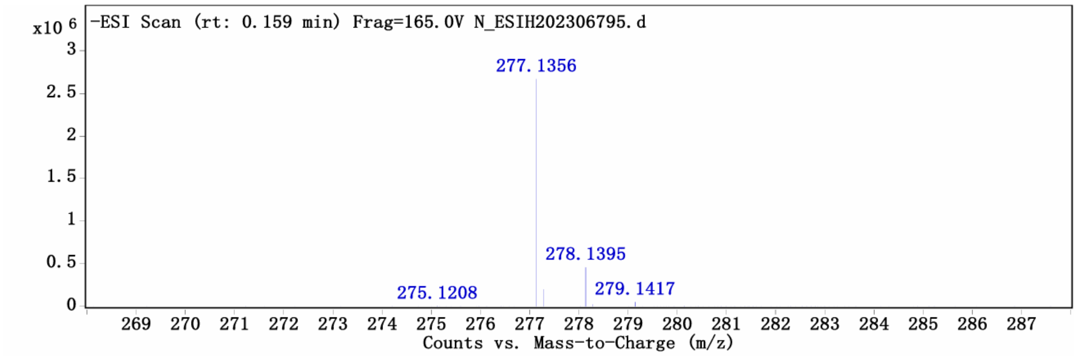

Formula Calculator Results

m/z	Calc m/z	Diff (mDa)	Diff (ppm)	Ion Formula	Ion
277.1356	277.1358	0.23	0.81	C15 H18 F N2 O2	(M-H)-

--- End Of Report ---

Figure S41. HRESIMS spectrum of compound **5i**.

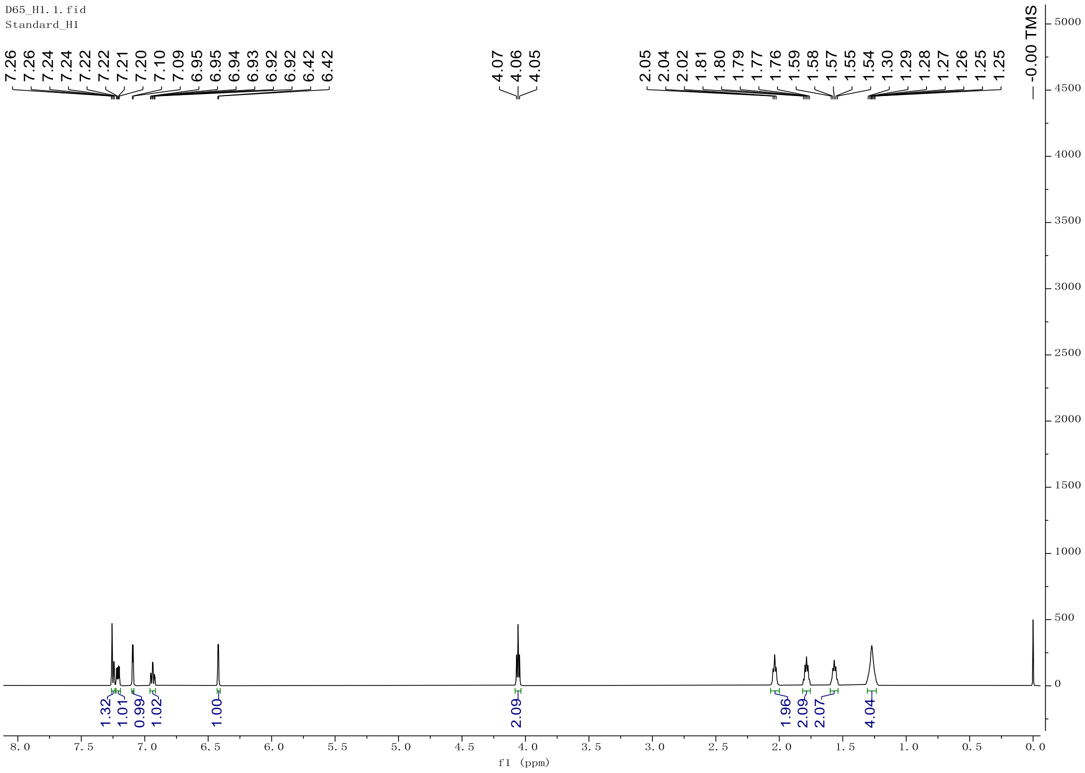

Figure S42. ^{1}H NMR spectrum of compound **5i**.

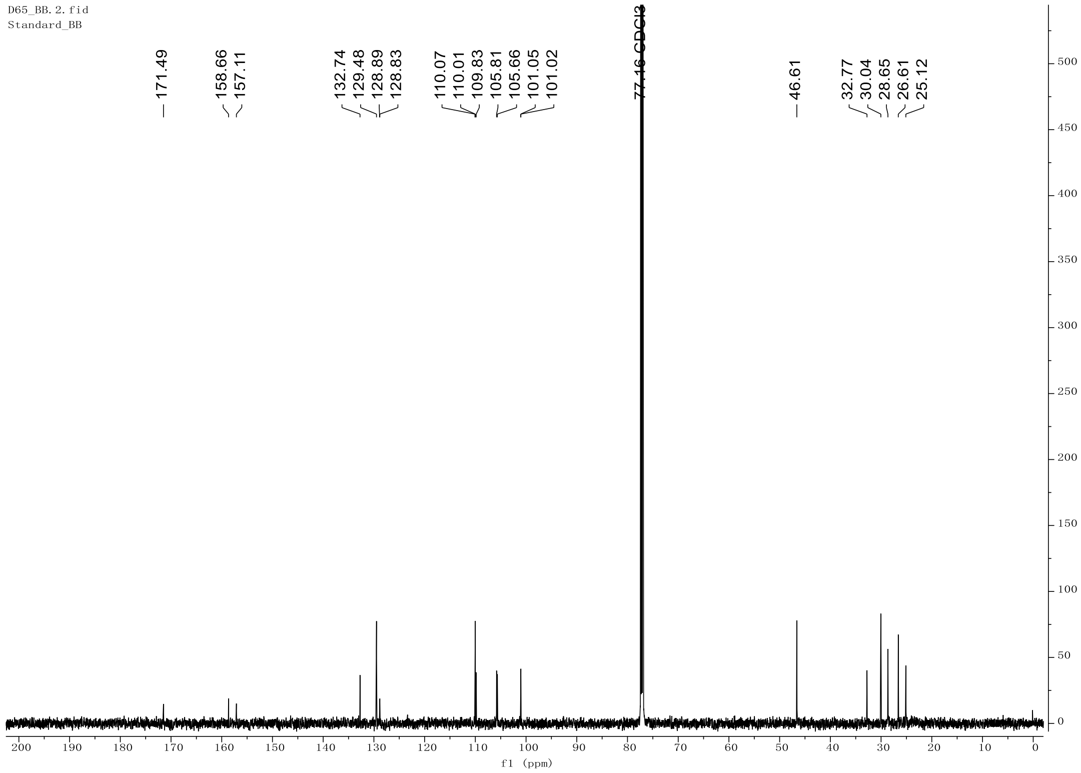

Figure S43. ^{13}C NMR spectrum of compound **5i**.

**Data Filename**	N_ESIH202306785.d	**Sample Name**	D4-D55
**Sample ID**		**Position**	P1-D4
**Instrument Name**	Agilent 6520 Q-TOF	**Acq Method**	20160324_MS_ESIH_NEG_1min.m
**Acquired Time**	11/27/2023 1:59:51 PM	**DA Method**	ESI-HR-20231114.m
**Comment**	ESIH by fangsu		

User Spectra

Fragmentor Voltage	Collision Energy	Ionization Mode
165	0	ESI

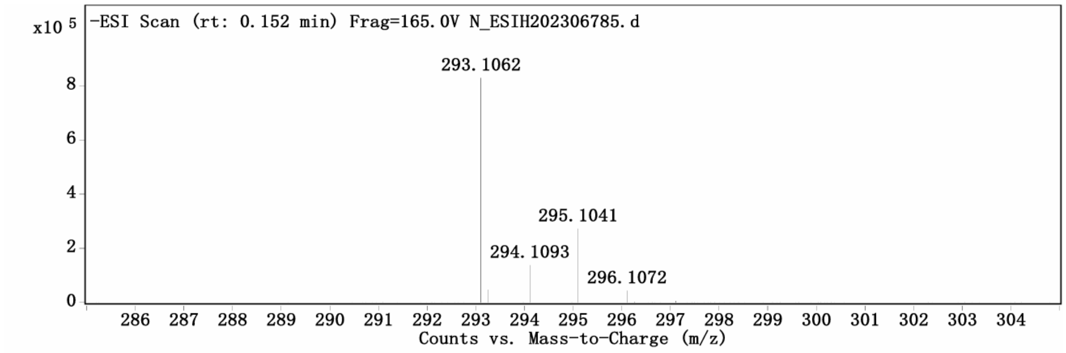

Formula Calculator Results

m/z	Calc m/z	Diff (mDa)	Diff (ppm)	Ion Formula	Ion
293.1062	293.1062	0.04	0.12	C15 H18 Cl N2 O2	(M-H)-

--- End Of Report ---

Figure S44. HRESIMS spectrum of compound **5j**.

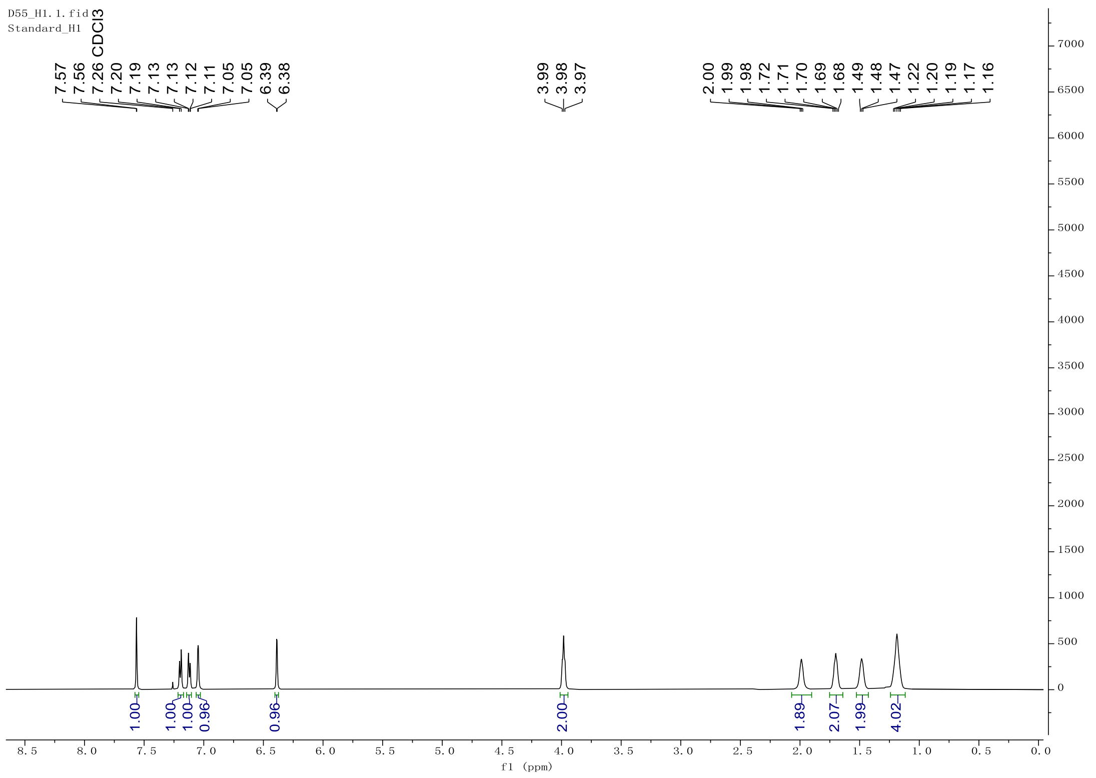

Figure S45. ^{1}H NMR spectrum of compound **5j**.

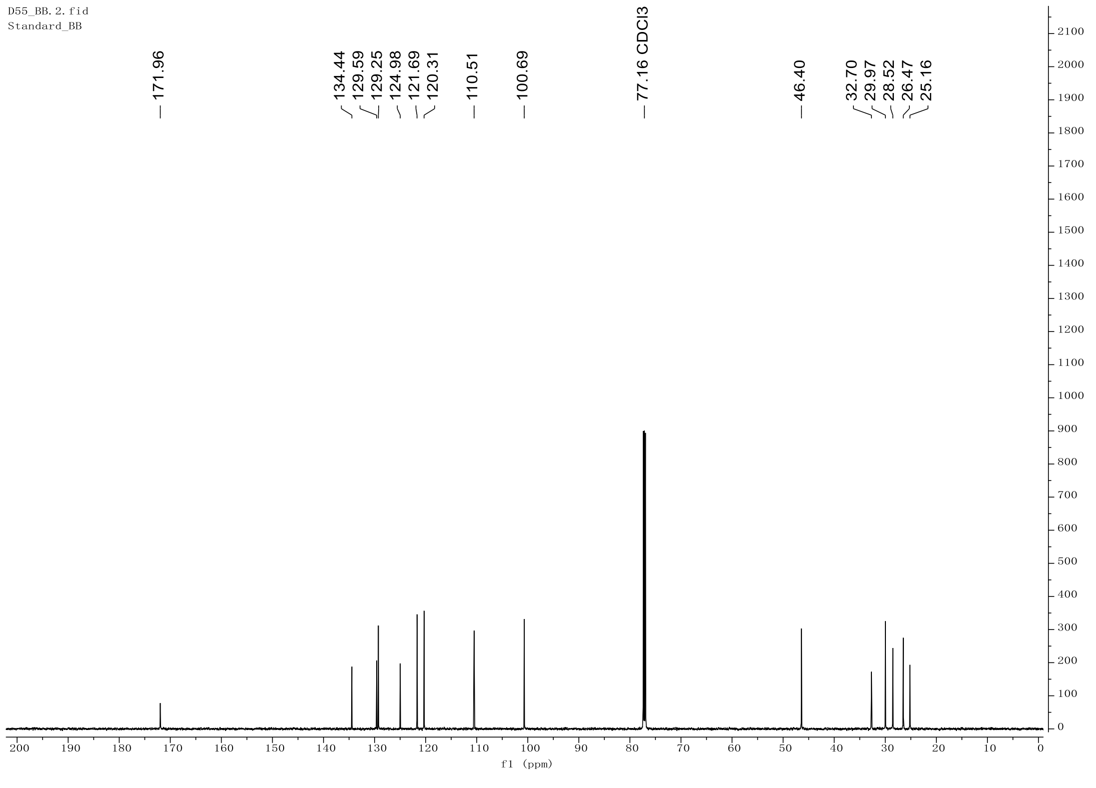

Figure S46. ^{13}C NMR spectrum of compound **5j**.

**Data Filename**	N_ESIH202306792.d	**Sample Name**	D4-D62
**Sample ID**		**Position**	P1-E2
**Instrument Name**	Agilent 6520 Q-TOF	**Acq Method**	20160324_MS_ESIH_NEG_1min.m
**Acquired Time**	11/27/2023 2:08:42 PM	**DA Method**	ESI-HR-20231114.m
**Comment**	ESIH by fangsu		

User Spectra

Fragmentor Voltage	Collision Energy	Ionization Mode
165	0	ESI

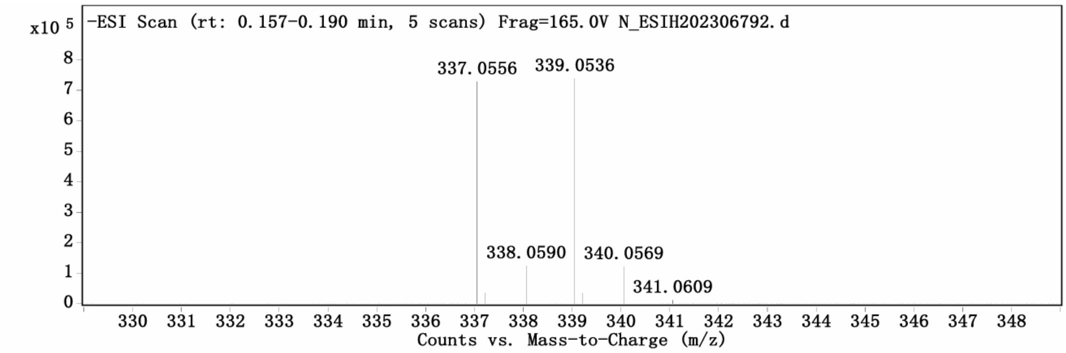

Formula Calculator Results

m/z	Calc m/z	Diff (mDa)	Diff (ppm)	Ion Formula	Ion
337.0556	337.0557	0.13	0.39	C15 H18 Br N2 O2	(M-H)-

--- End Of Report ---

Figure S47. HRESIMS spectrum of compound **5k**.

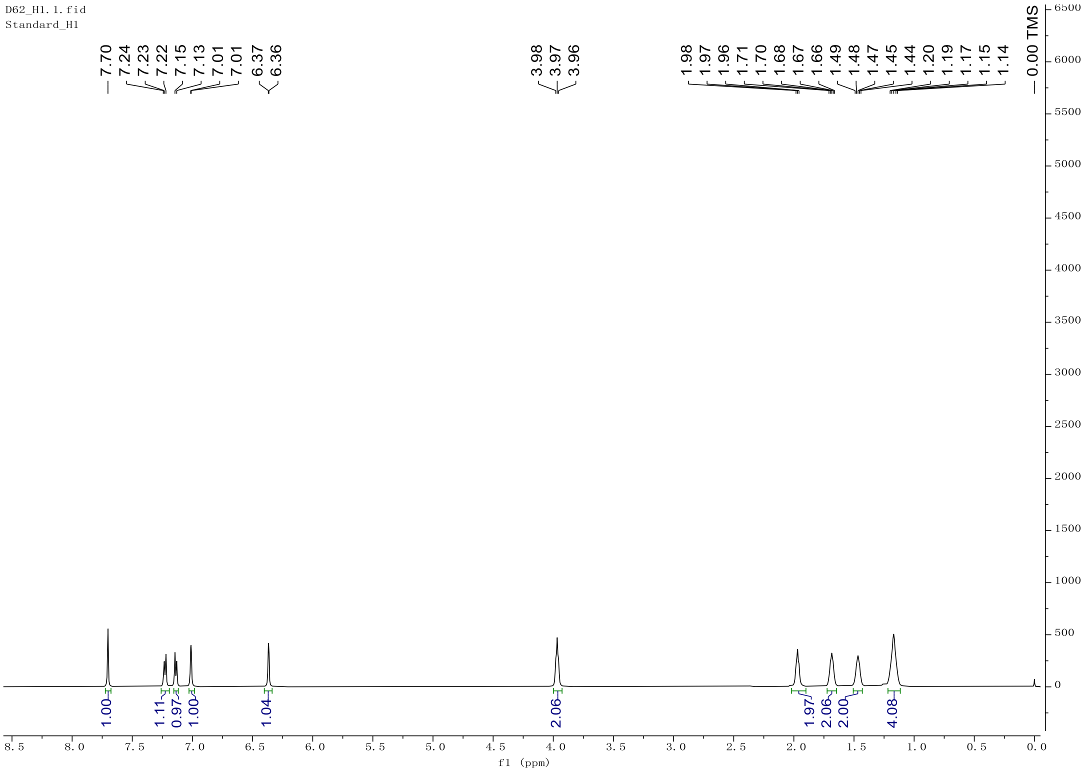

Figure S48. ^{1}H NMR spectrum of compound **5k**.

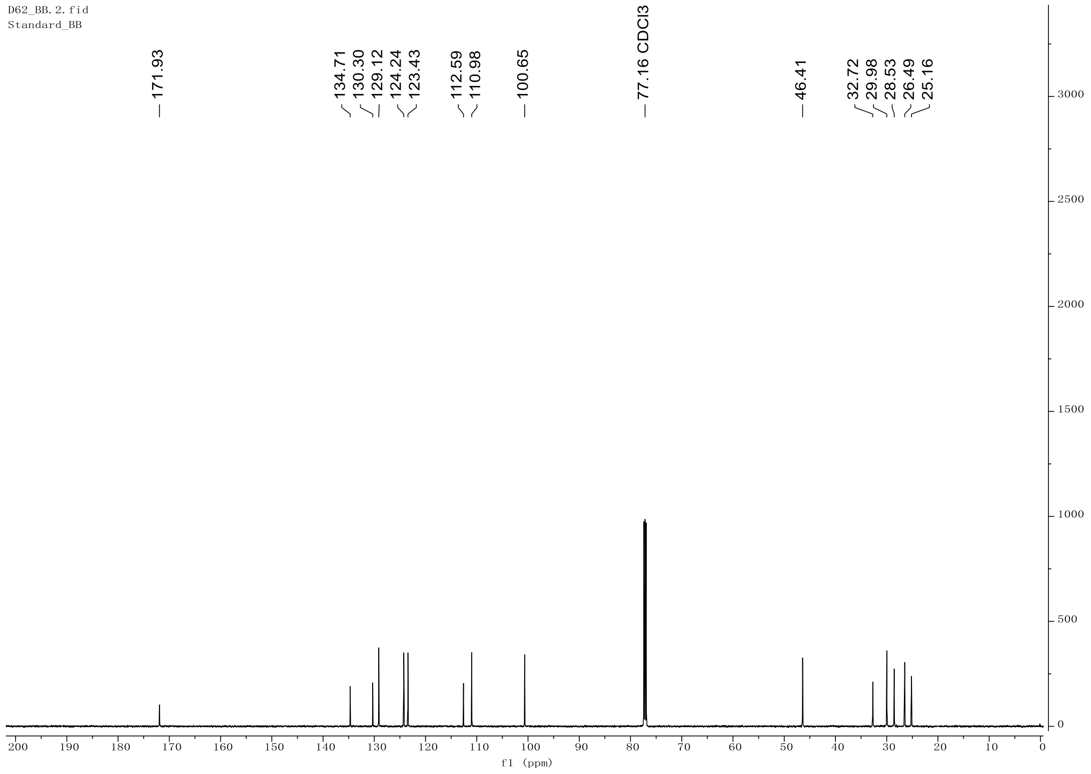

Figure S49. ^{13}C NMR spectrum of compound **5k**.

**Data Filename**	N_ESIH202306787.d	**Sample Name**	D4-D57
**Sample ID**		**Position**	P1-D6
**Instrument Name**	Agilent 6520 Q-TOF	**Acq Method**	20160324_MS_ESIH_NEG_1min.m
**Acquired Time**	11/27/2023 2:02:21 PM	**DA Method**	ESI-HR-20231114.m
**Comment**	ESIH by fangsu		

User Spectra

Fragmentor Voltage	Collision Energy	Ionization Mode
165	0	ESI

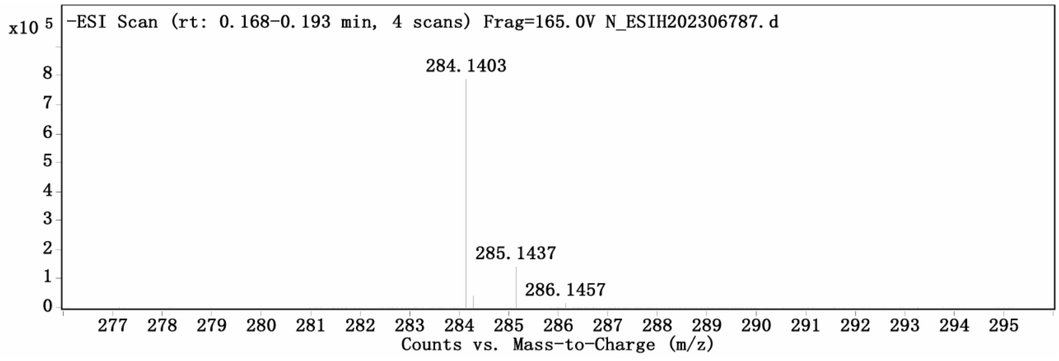

Formula Calculator Results

m/z	Calc m/z	Diff (mDa)	Diff (ppm)	Ion Formula	Ion
284.1403	284.1405	0.16	0.56	C16 H18 N3 O2	(M-H)-

--- End Of Report ---

Figure S50. HRESIMS spectrum of compound **5l**.

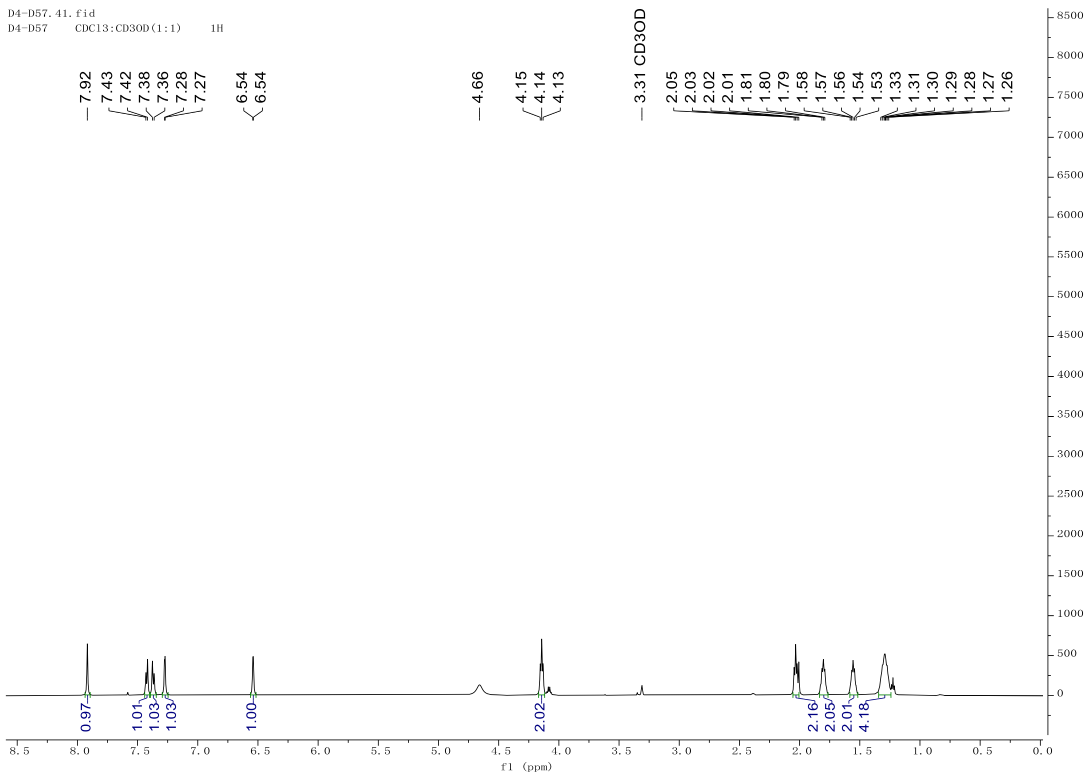

Figure S51. ^{1}H NMR spectrum of compound **5l**.

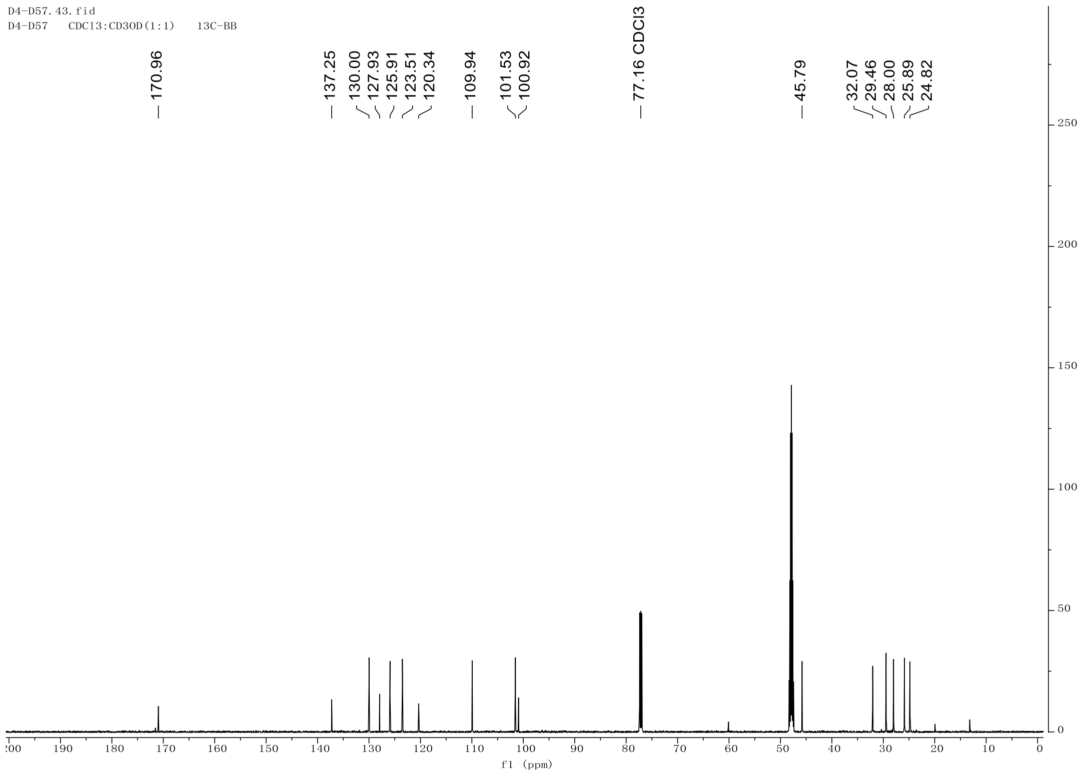

Figure S52. ^{13}C NMR spectrum of compound **5l**.

**Data Filename**	N_ESIH202306782.d	**Sample Name**	D4-D36
**Sample ID**		**Position**	P1-D1
**Instrument Name**	Agilent 6520 Q-TOF	**Acq Method**	20160324_MS_ESIH_NEG_1min.m
**Acquired Time**	11/27/2023 1:56:03 PM	**DA Method**	ESI-HR-20231114.m
**Comment**	ESIH by fangsu		

User Spectra

Fragmentor Voltage	Collision Energy	Ionization Mode
165	0	ESI

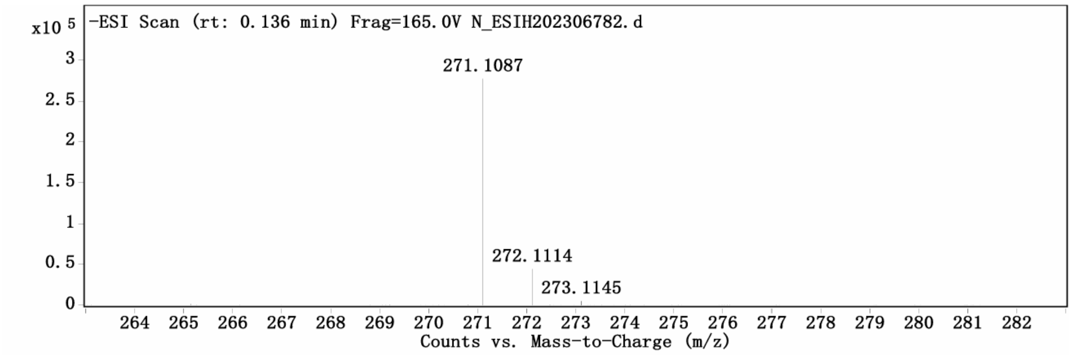

Formula Calculator Results

m/z	Calc m/z	Diff (mDa)	Diff (ppm)	Ion Formula	Ion
271.1087	271.1088	0.16	0.58	C15 H15 N2 O3	(M-H)-

--- End Of Report ---

Figure S53. HRESIMS spectrum of compound **11**.

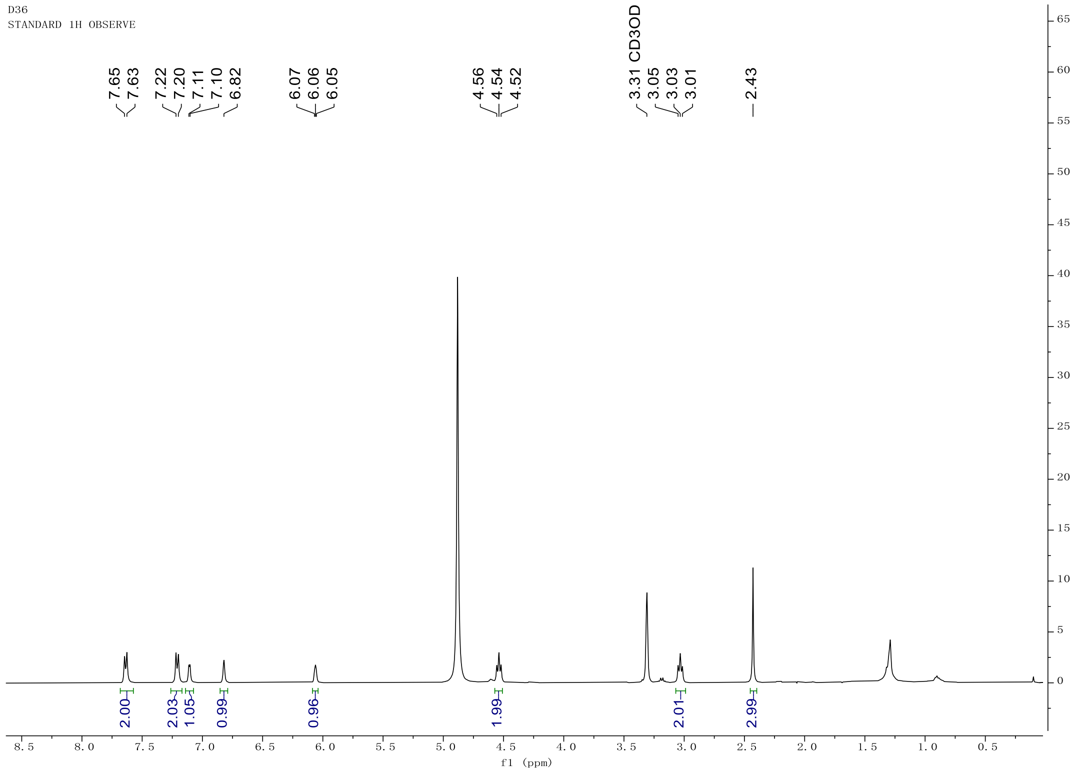

Figure S54. ^{1}H NMR spectrum of compound **11**.

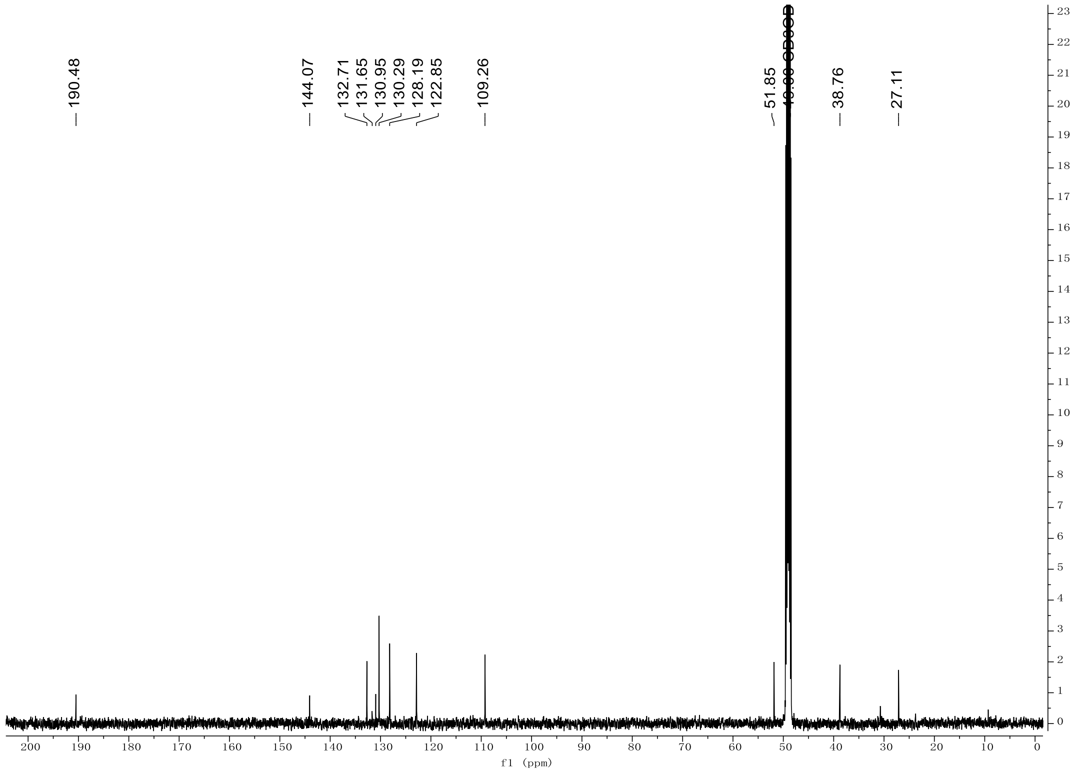

Figure S55. ^{13}C NMR spectrum of compound **11**.

**Data Filename**	N_ESIH202306783.d	**Sample Name**	D4-D53
**Sample ID**		**Position**	P1-D2
**Instrument Name**	Agilent 6520 Q-TOF	**Acq Method**	20160324_MS_ESIH_NEG_1min.m
**Acquired Time**	11/27/2023 1:57:18 PM	**DA Method**	ESI-HR-20231114.m
**Comment**	ESIH by fangsu		

User Spectra

Fragmentor Voltage	Collision Energy	Ionization Mode
165	0	ESI

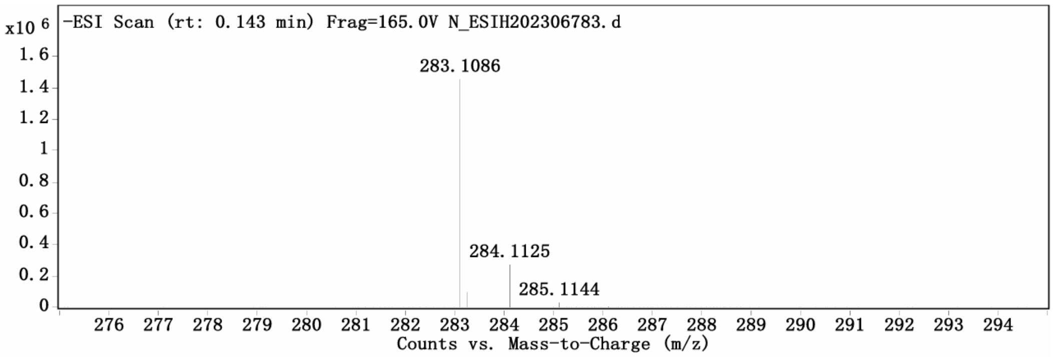

Formula Calculator Results

m/z	Calc m/z	Diff (mDa)	Diff (ppm)	Ion Formula	Ion
283.1086	283.1088	0.17	0.59	C16 H15 N2 O3	(M-H)-

--- End Of Report ---

Figure S56. HRESIMS spectrum of compound **14**.

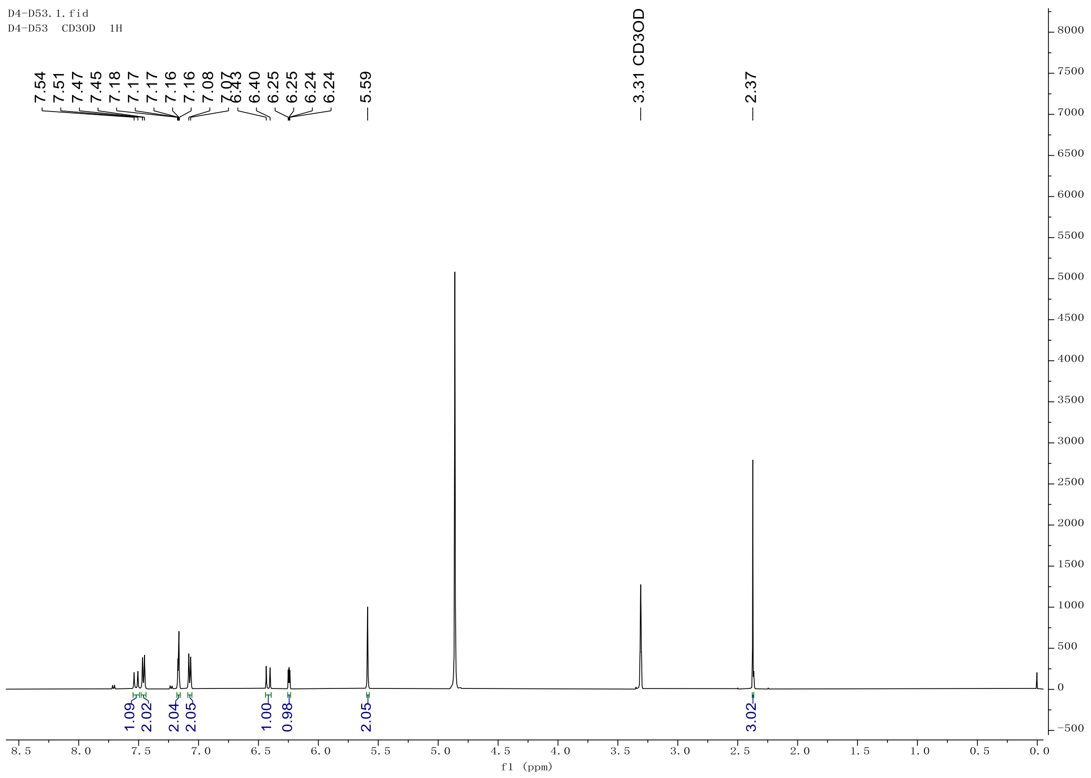

Figure S57. ^{1}H NMR spectrum of compound **14**.

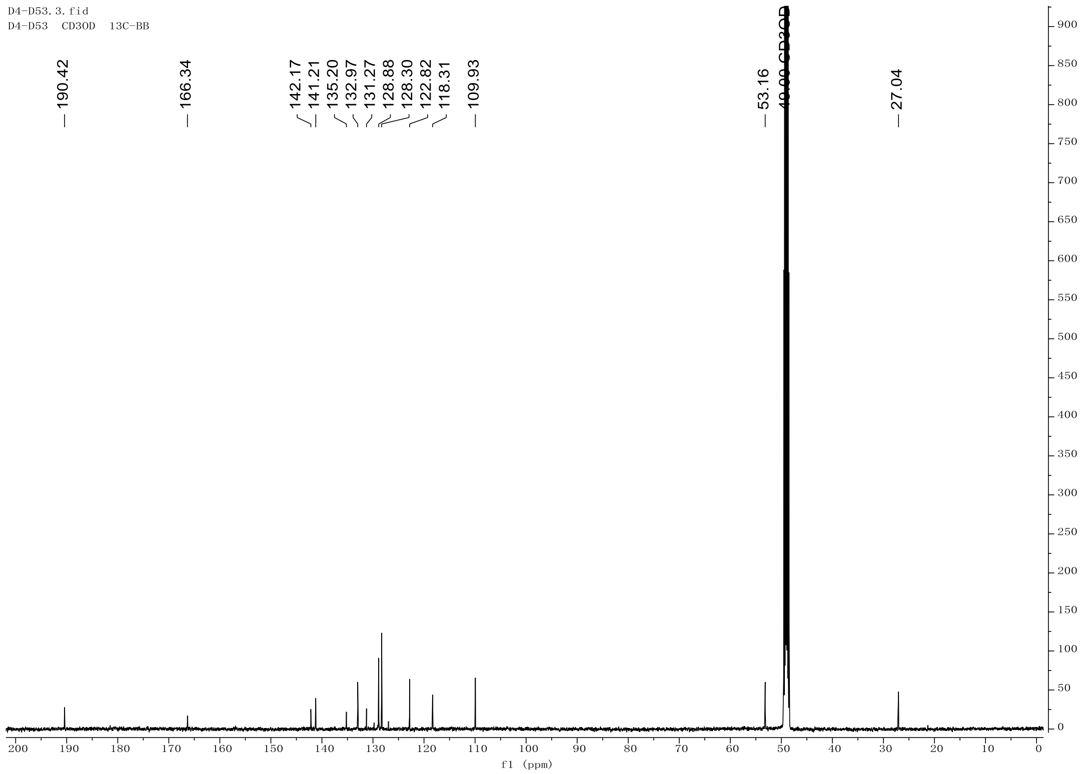

Figure S58. ^{13}C NMR spectrum of compound **14**.

**Data Filename**	N_ESIH202306773.d	**Sample Name**	D4-H21
**Sample ID**		**Position**	P1-C1
**Instrument Name**	Agilent 6520 Q-TOF	**Acq Method**	20160324_MS_ESIH_NEG_1min.m
**Acquired Time**	11/27/2023 1:44:35 PM	**DA Method**	ESI-HR-20231114.m
**Comment**	ESIH by fangsu		

User Spectra

Fragmentor Voltage	Collision Energy	Ionization Mode
165	0	ESI

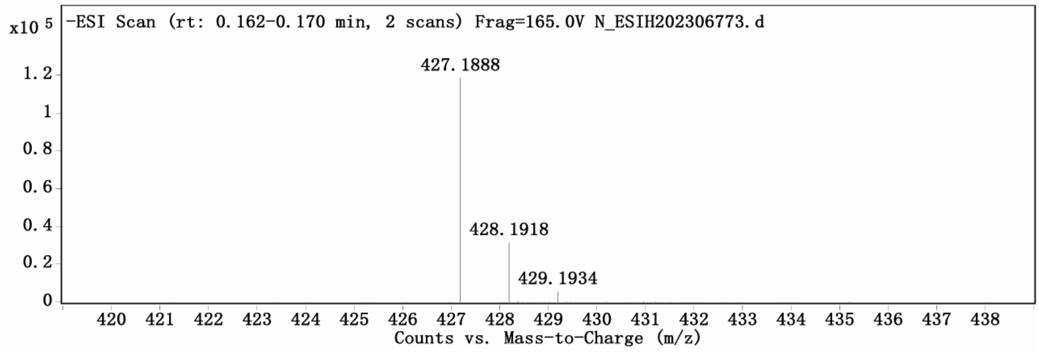

Formula Calculator Results

m/z	Calc m/z	Diff (mDa)	Diff (ppm)	Ion Formula	Ion
427.1888	427.1888	-0.03	-0.08	C24 H23 N6 O2	(M-H)-

--- End Of Report ---

Figure S59. HRESIMS spectrum of compound **19a**.

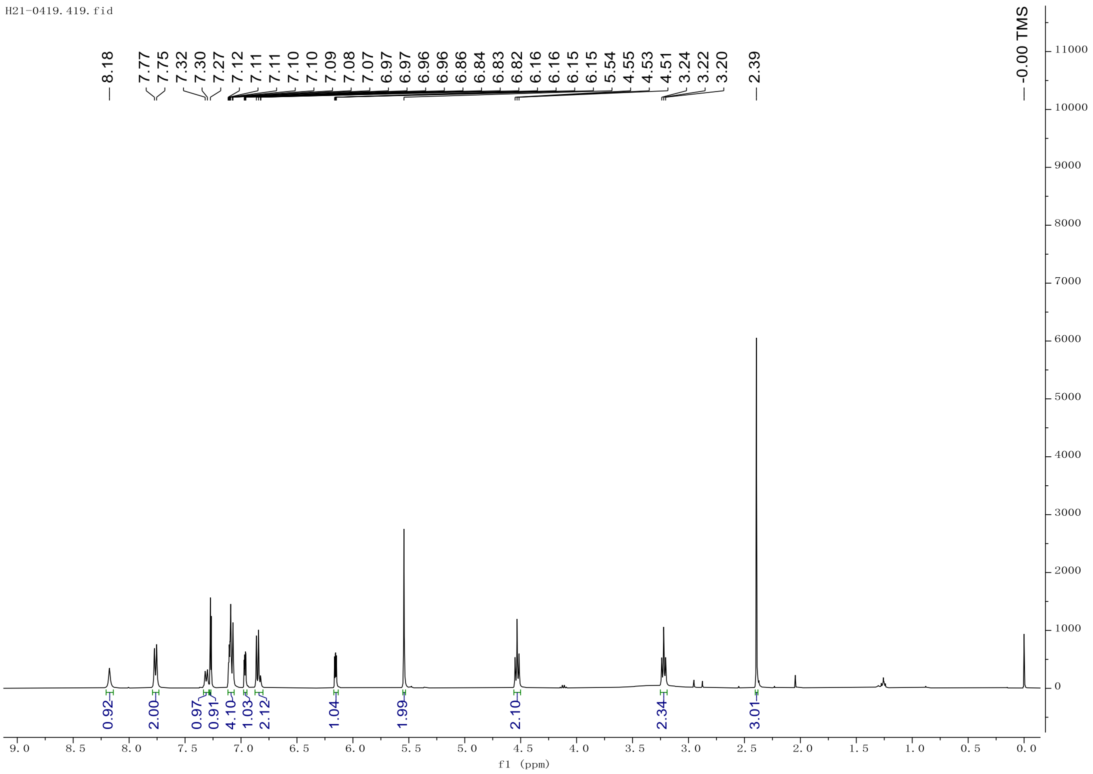

Figure S60. ^{1}H NMR spectrum of compound **19a**.

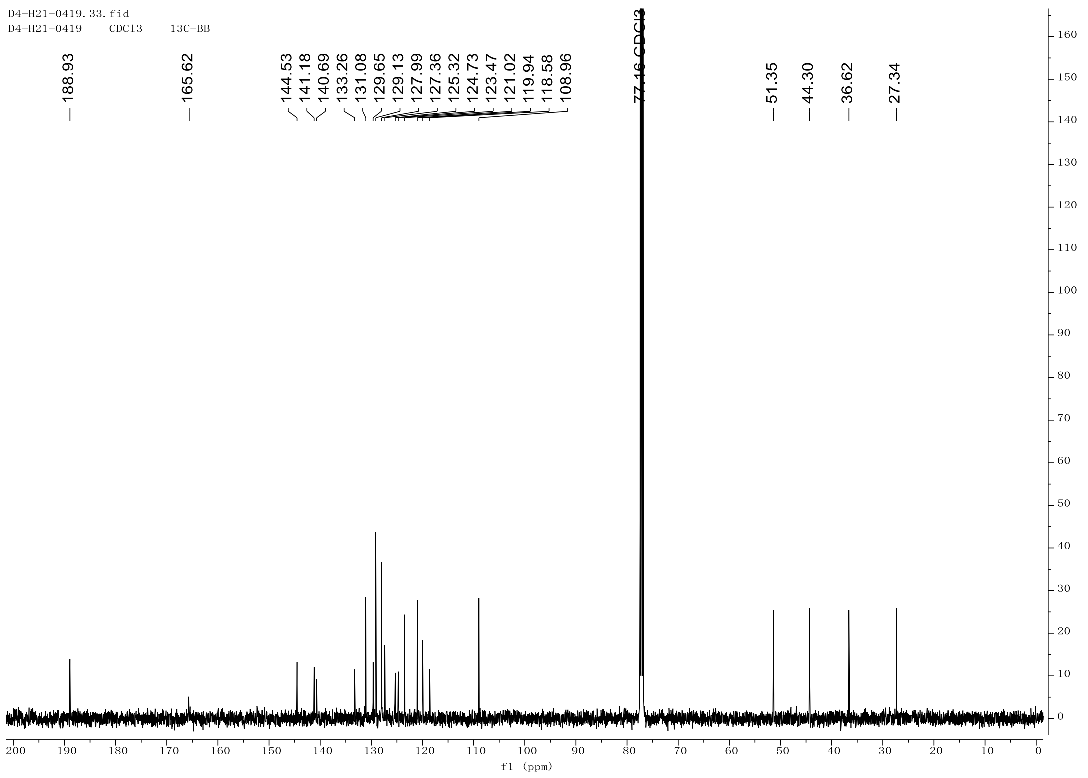

Figure S61. ^{13}C NMR spectrum of compound **19a**.

**Data Filename**	N_ESIH202306774.d	**Sample Name**	D4-H40
**Sample ID**		**Position**	P1-C2
**Instrument Name**	Agilent 6520 Q-TOF	**Acq Method**	20160324_MS_ESIH_NEG_1min.m
**Acquired Time**	11/27/2023 1:45:52 PM	**DA Method**	ESI-HR-20231114.m
**Comment**	ESIH by fangsu		

User Spectra

Fragmentor Voltage	Collision Energy	Ionization Mode
165	0	ESI

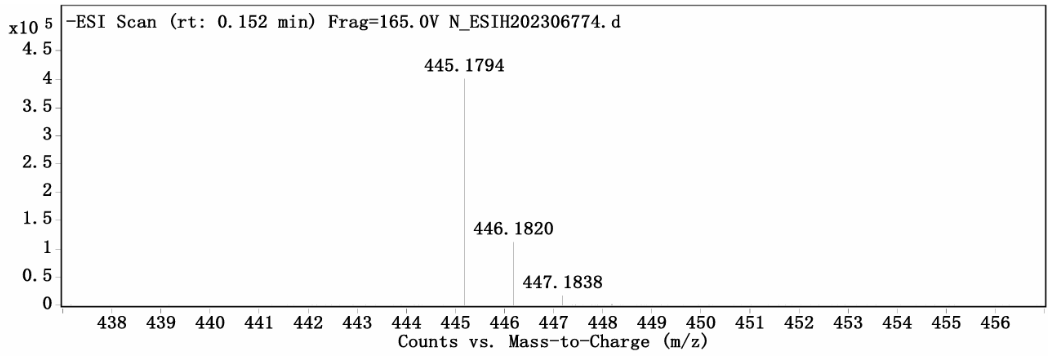

Formula Calculator Results

m/z	Calc m/z	Diff (mDa)	Diff (ppm)	Ion Formula	Ion
445.1794	445.1794	-0.07	-0.15	C24 H22 F N6 O2	(M-H)-

--- End Of Report ---

Figure S62. HRESIMS spectrum of compound **19b**.

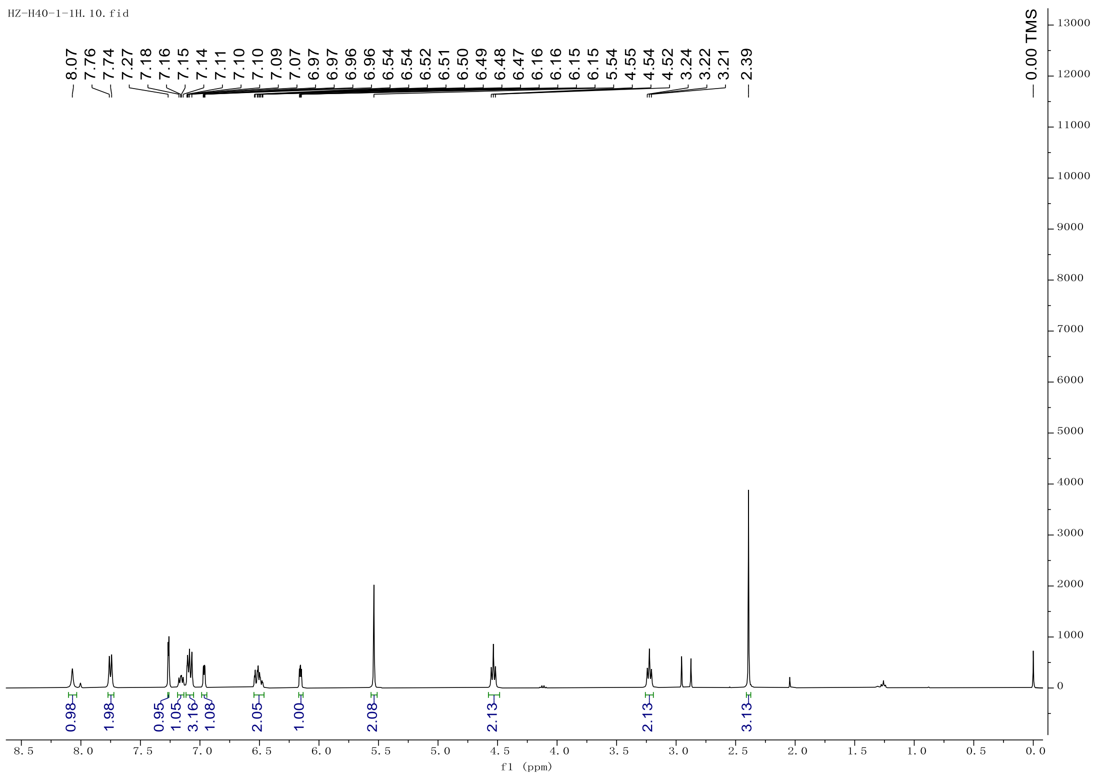

Figure S63. ¹H NMR spectrum of compound **19b**.

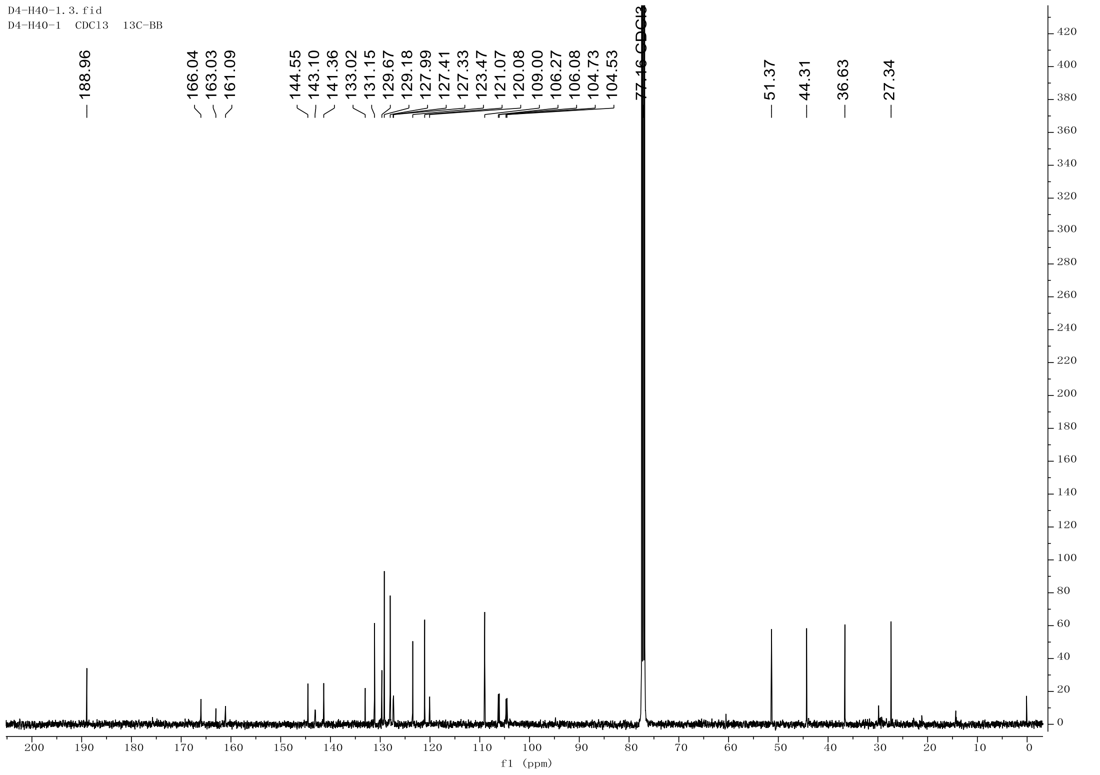

Figure S64. ^{13}C NMR spectrum of compound **19b**.

**Data Filename**	N_ESIH202306775.d	**Sample Name**	D4-H41
**Sample ID**		**Position**	P1-C3
**Instrument Name**	Agilent 6520 Q-TOF	**Acq Method**	20160324_MS_ESIH_NEG_1min.m
**Acquired Time**	11/27/2023 1:47:10 PM	**DA Method**	ESI-HR-20231114.m
**Comment**	ESIH by fangsu		

User Spectra

Fragmentor Voltage	Collision Energy	Ionization Mode
165	0	ESI

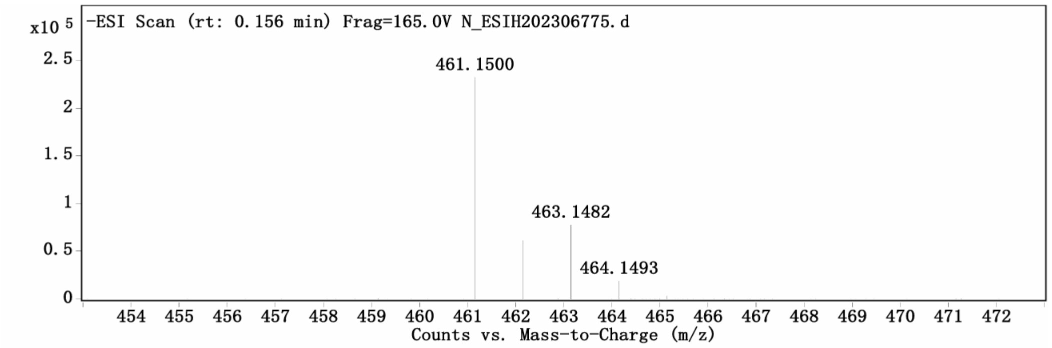

Formula Calculator Results

m/z	Calc m/z	Diff (mDa)	Diff (ppm)	Ion Formula	Ion
461.15	461.1498	-0.16	-0.35	C24 H22 Cl N6 O2	(M-H)-

--- End Of Report ---

Figure S65. HRESIMS spectrum of compound **19c**.

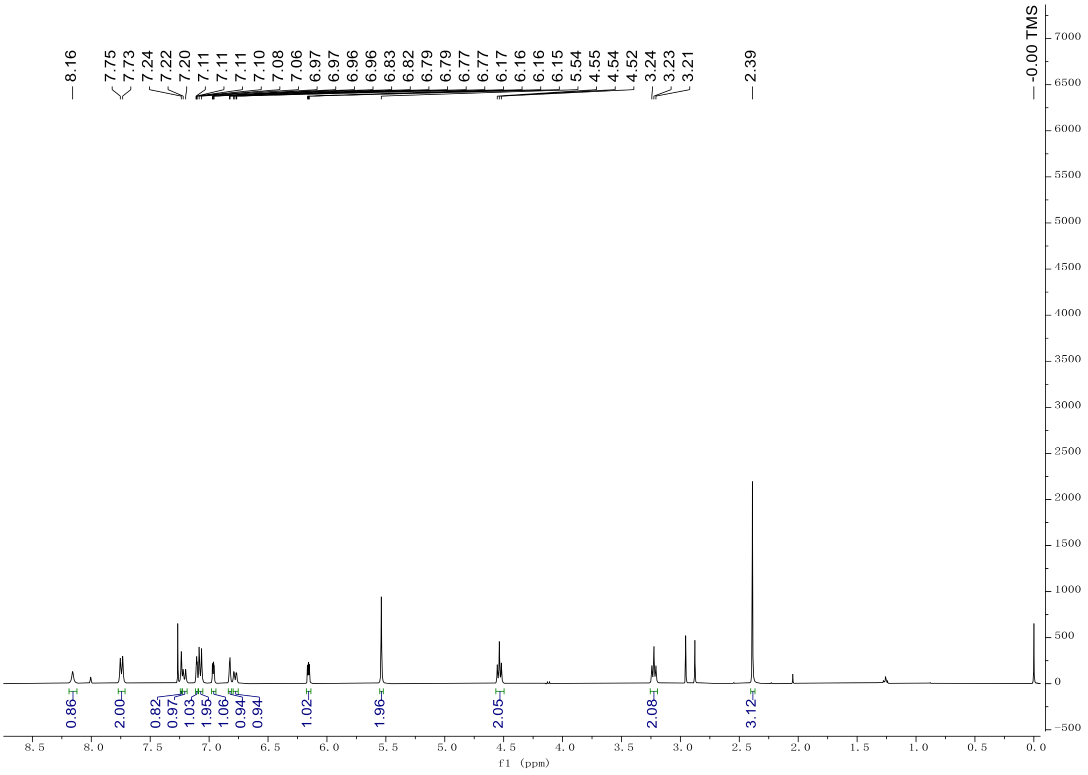

Figure S66. ^{1}H NMR spectrum of compound **19c**.

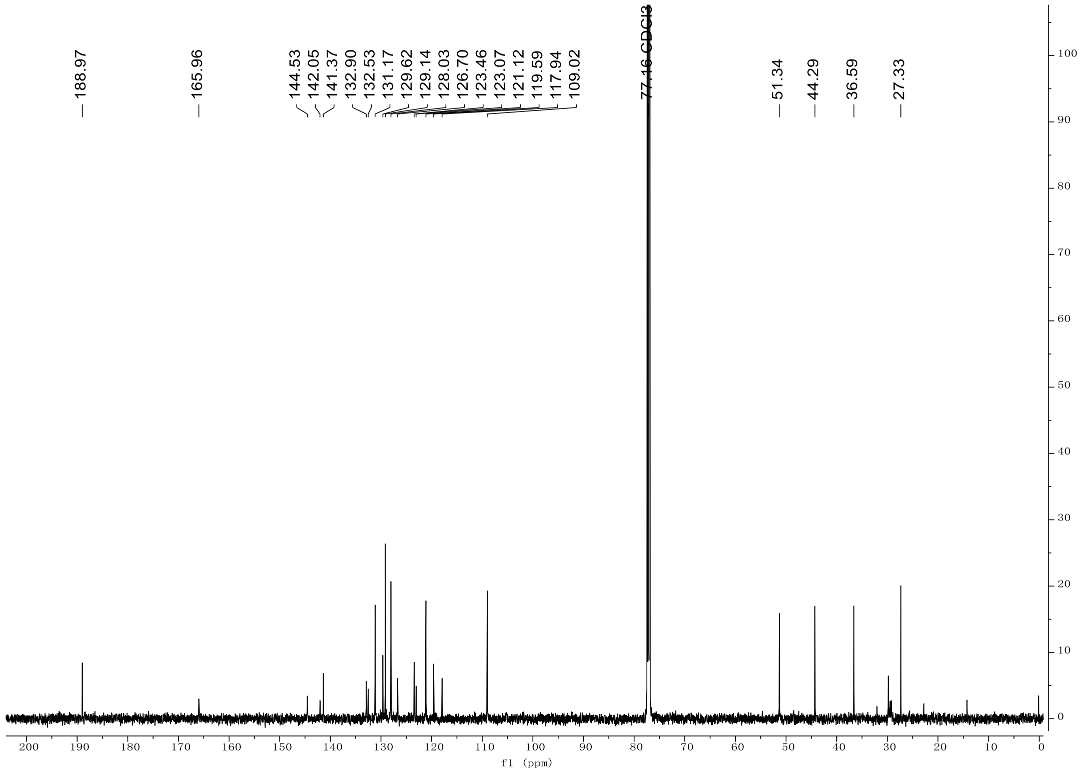

Figure S67. ^{13}C NMR spectrum of compound **19c**.

**Data Filename**	N_ESIH202306776.d	**Sample Name**	D4-H42
**Sample ID**		**Position**	P1-C4
**Instrument Name**	Agilent 6520 Q-TOF	**Acq Method**	20160324_MS_ESIH_NEG_1min.m
**Acquired Time**	11/27/2023 1:48:28 PM	**DA Method**	ESI-HR-20231114.m
**Comment**	ESIH by fangsu		

User Spectra

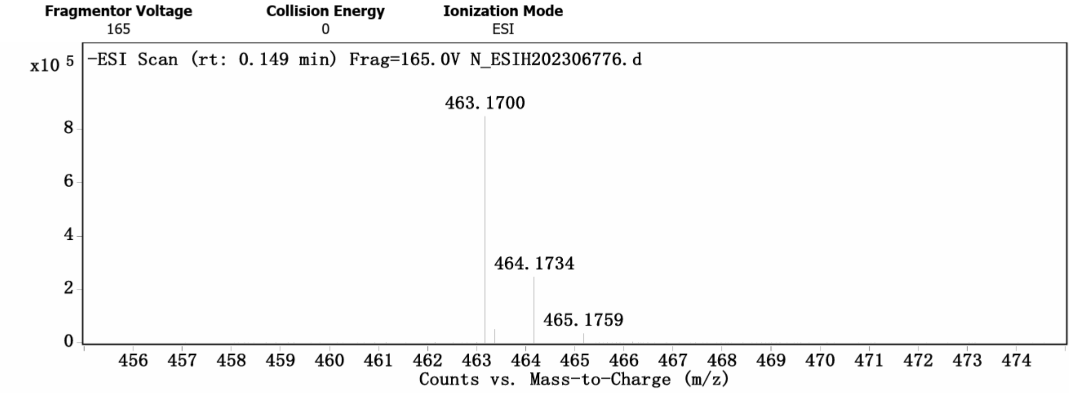

Formula Calculator Results

m/z	Calc m/z	Diff (mDa)	Diff (ppm)	Ion Formula	Ion
463.17	463.17	-0.03	-0.06	C24 H21 F2 N6 O2	(M-H)-

--- End Of Report ---

Figure S68. HRESIMS spectrum of compound **19d**.

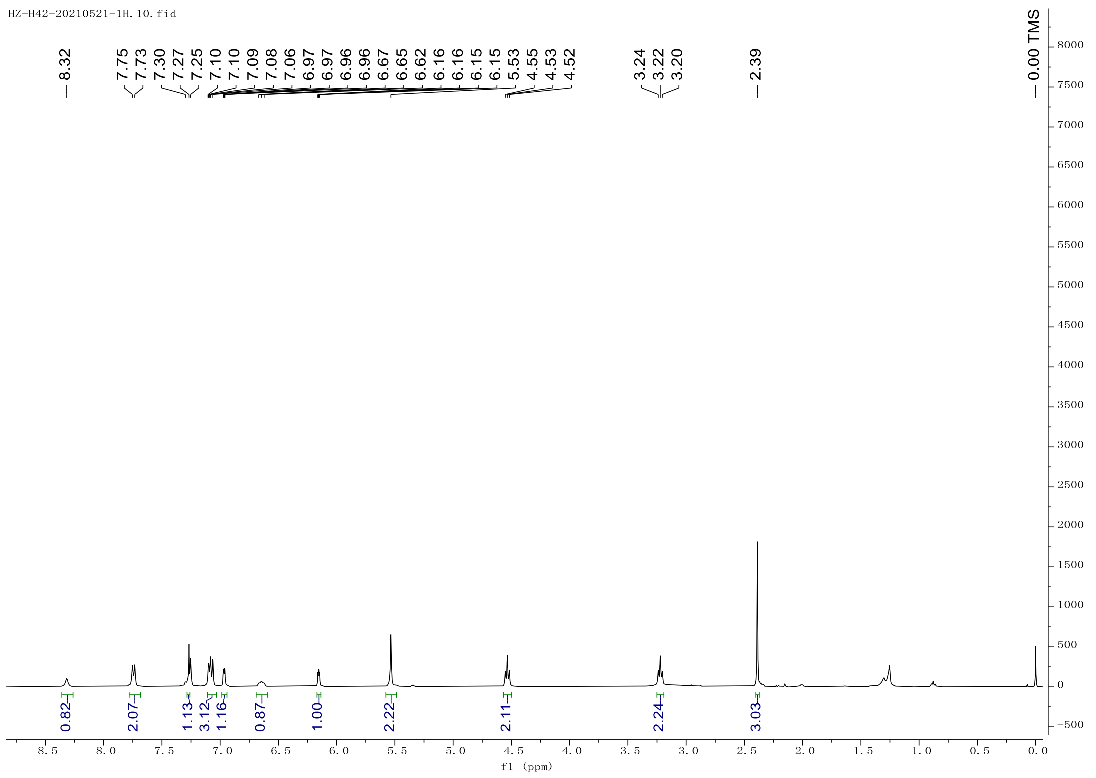

Figure S69. ^{1}H NMR spectrum of compound **19d**.

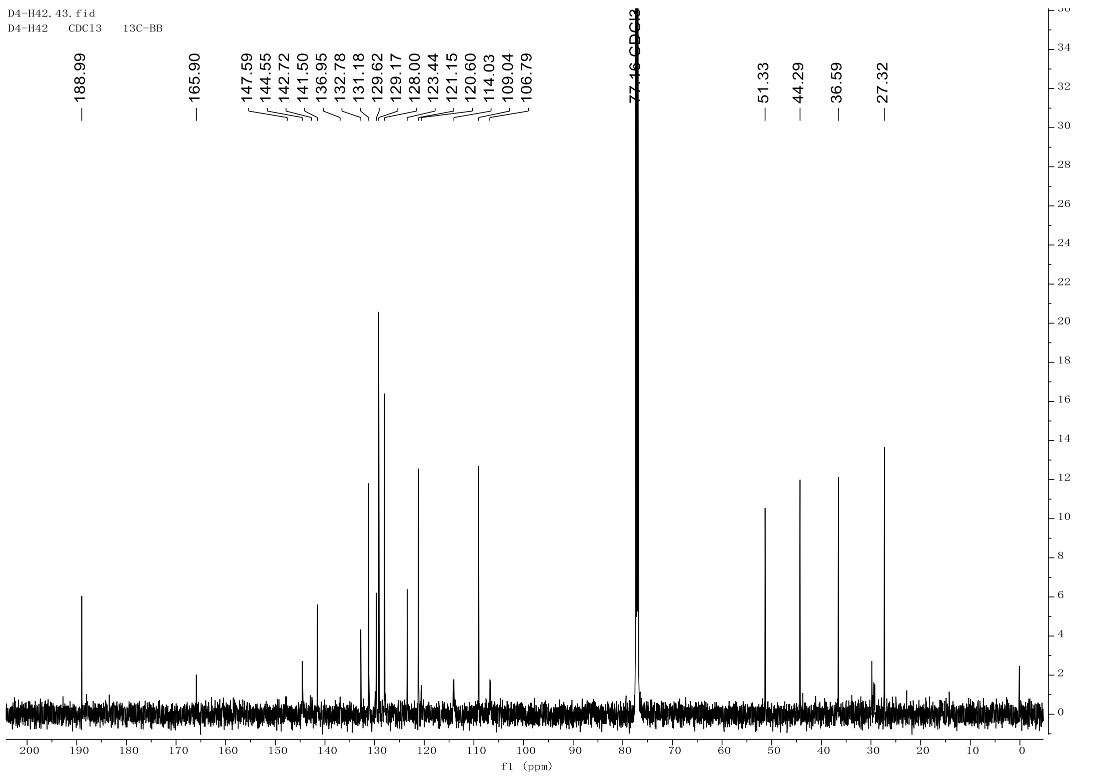

Figure S70. ^{13}C NMR spectrum of compound **19d**.

**Data Filename**	N_ESIH202306777.d	**Sample Name**	D4-H43
**Sample ID**		**Position**	P1-C5
**Instrument Name**	Agilent 6520 Q-TOF	**Acq Method**	20160324_MS_ESIH_NEG_1min.m
**Acquired Time**	11/27/2023 1:49:42 PM	**DA Method**	ESI-HR-20231114.m
**Comment**	ESIH by fangsu		

User Spectra

Fragmentor Voltage	Collision Energy	Ionization Mode
165	0	ESI

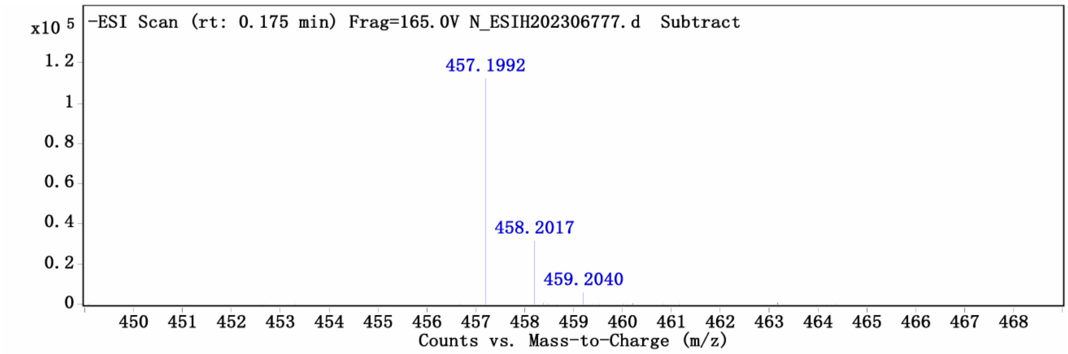

Formula Calculator Results

m/z	Calc m/z	Diff (mDa)	Diff (ppm)	Ion Formula	Ion
457.1992	457.1994	0.14	0.3	C25 H25 N6 O3	(M-H)-

--- End Of Report ---

Figure S71. HRESIMS spectrum of compound **19e**.

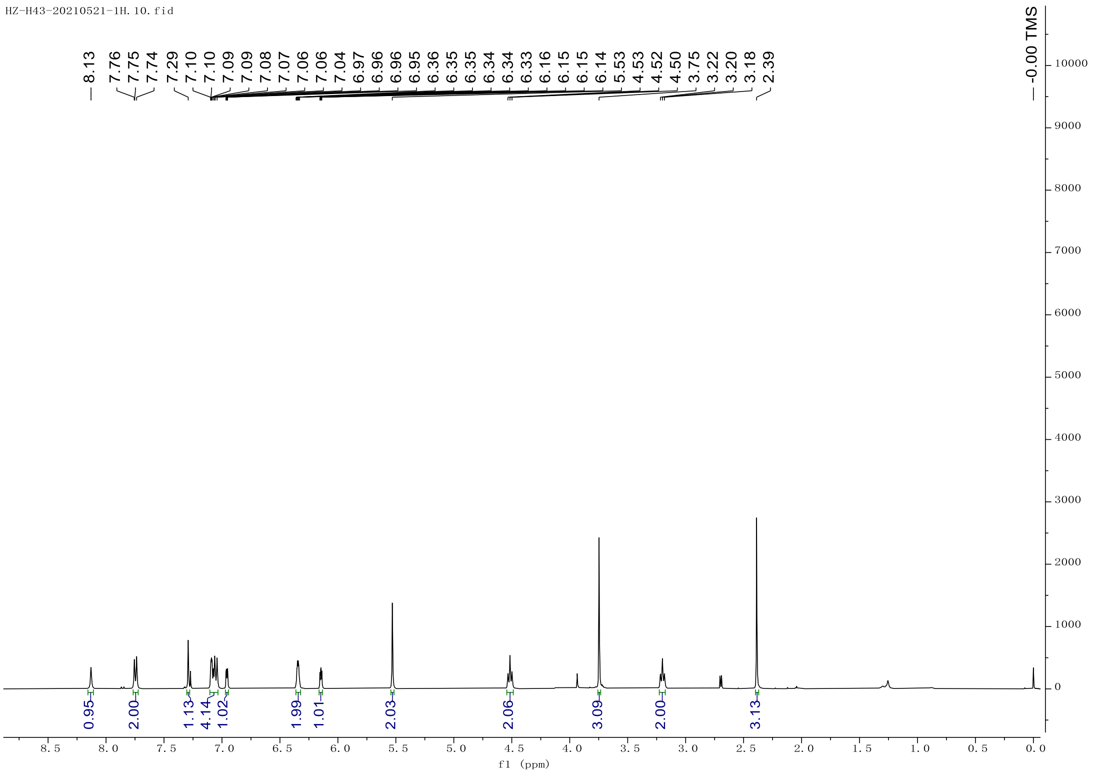

Figure S72. ^{1}H NMR spectrum of compound **19e**.

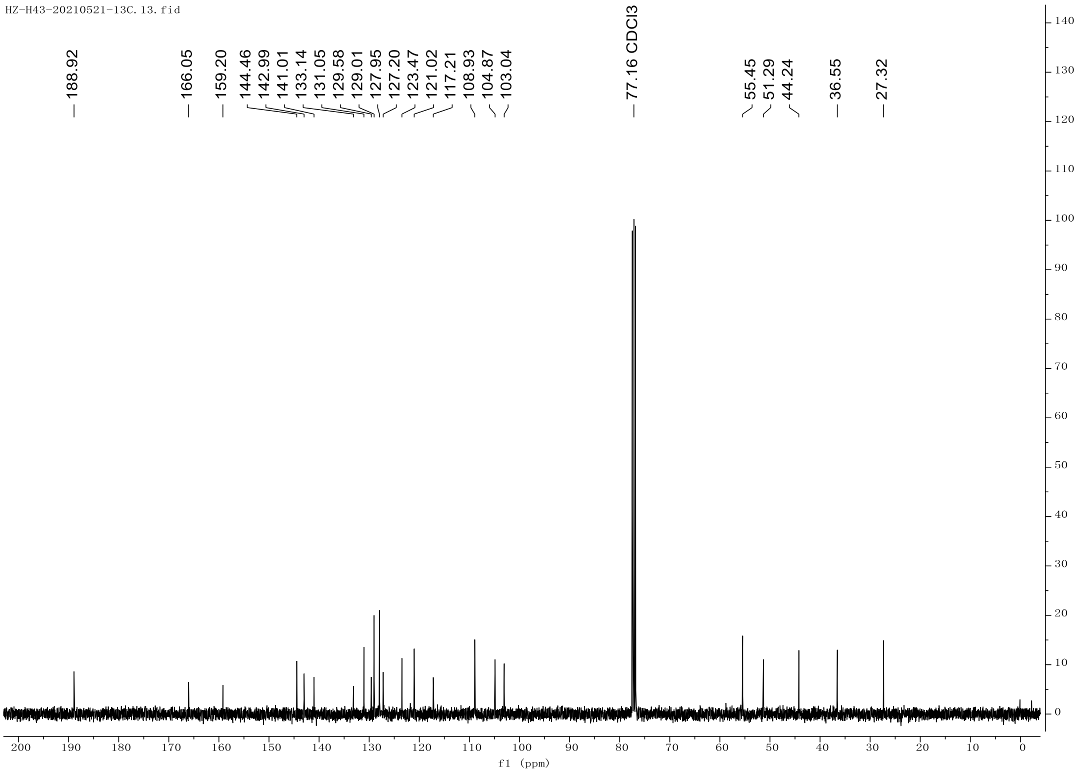

Figure S73. ^{13}C NMR spectrum of compound **19e**.

**Data Filename**	N_ESIH202306778.d	**Sample Name**	D4-H44
**Sample ID**		**Position**	P1-C6
**Instrument Name**	Agilent 6520 Q-TOF	**Acq Method**	20160324_MS_ESIH_NEG_1min.m
**Acquired Time**	11/27/2023 1:50:58 PM	**DA Method**	ESI-HR-20231114.m
**Comment**	ESIH by fangsu		

User Spectra

Fragmentor Voltage	Collision Energy	Ionization Mode
165	0	ESI

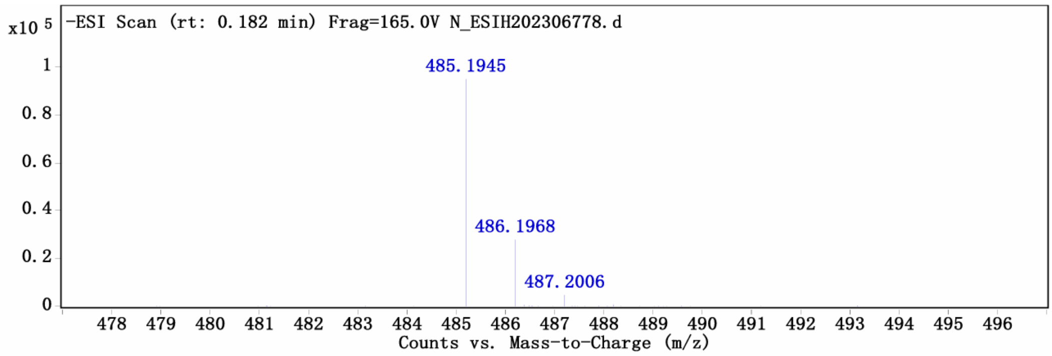

Formula Calculator Results

m/z	Calc m/z	Diff (mDa)	Diff (ppm)	Ion Formula	Ion
485.1945	485.1943	-0.25	-0.53	C26 H25 N6 O4	(M-H)-

--- End Of Report ---

Figure S74. HRESIMS spectrum of compound **19f**.

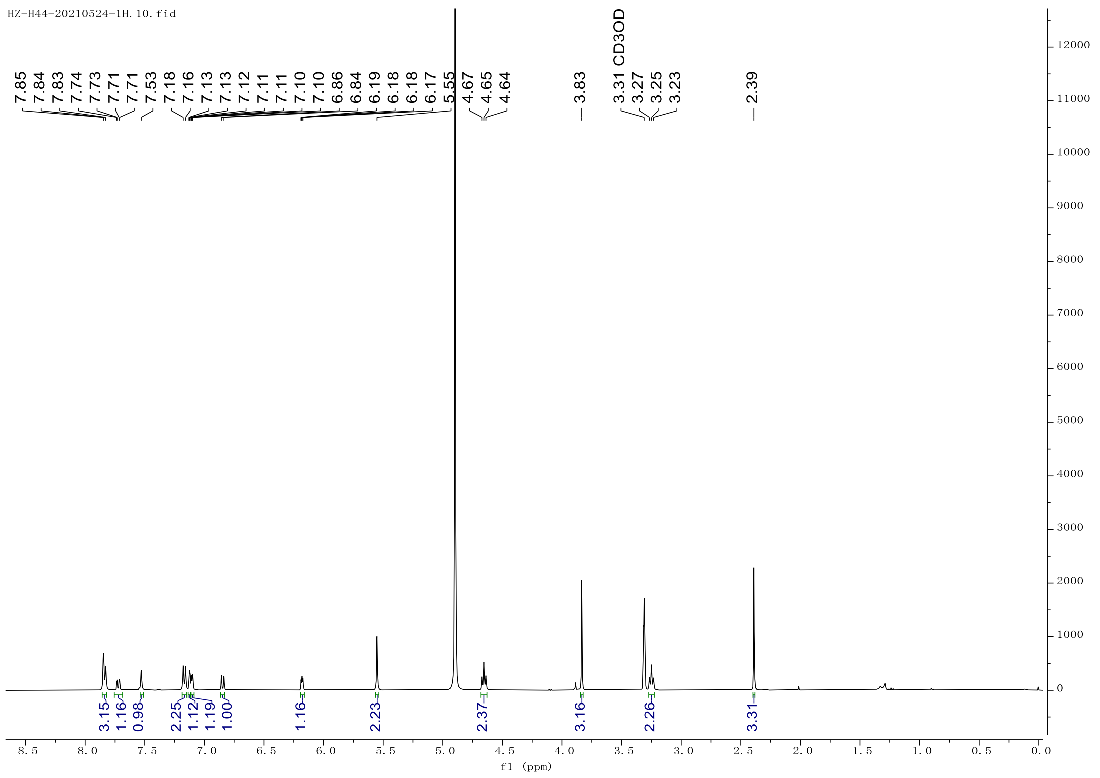

Figure S75. ^{1}H NMR spectrum of compound **19f**.

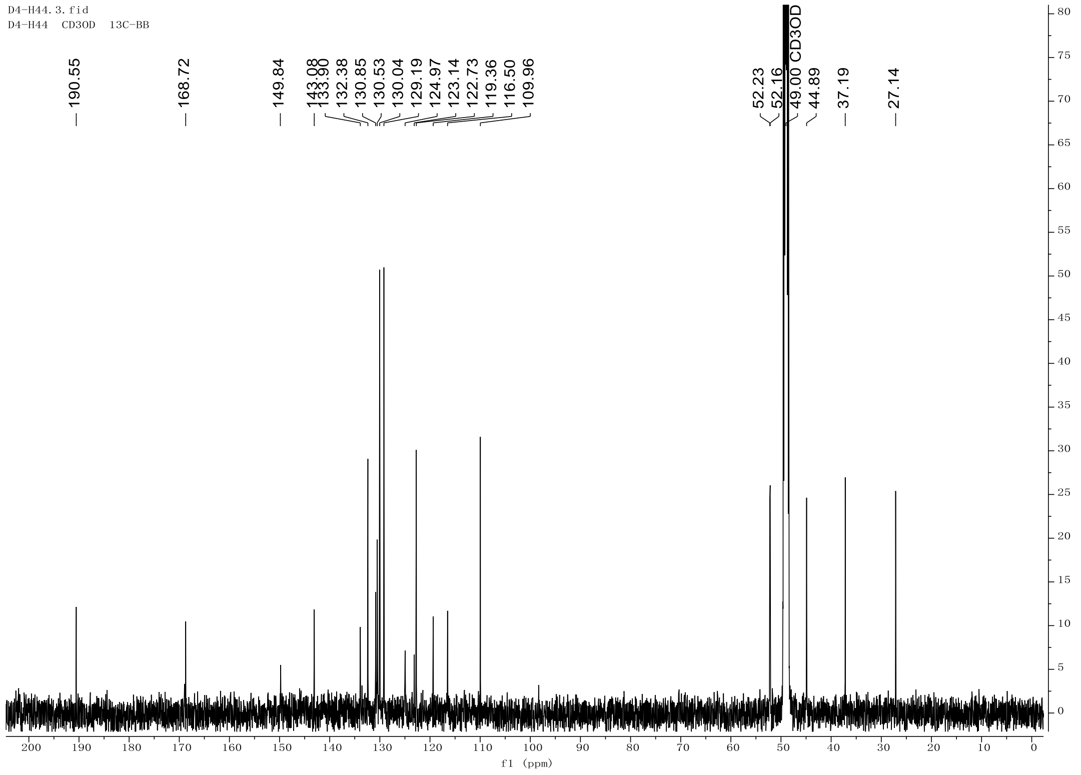

Figure S76. ^{13}C NMR spectrum of compound **19f**.

**Data Filename**	N_ESIH202306781.d	**Sample Name**	D4-D31
**Sample ID**		**Position**	P1-C9
**Instrument Name**	Agilent 6520 Q-TOF	**Acq Method**	20160324_MS_ESIH_NEG_1min.m
**Acquired Time**	11/27/2023 1:54:47 PM	**DA Method**	ESI-HR-20231114.m
**Comment**	ESIH by fangsu		

User Spectra

**Fragmentor Voltage**	**Collision Energy**	**Ionization Mode**
165	0	ESI

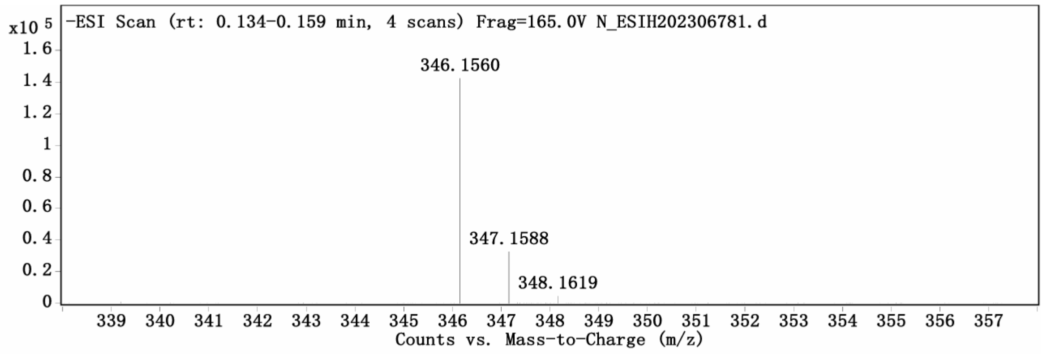

Formula Calculator Results

m/z	Calc m/z	Diff (mDa)	Diff (ppm)	Ion Formula	Ion
346.156	346.1561	0.08	0.24	C21 H20 N3 O2	(M-H)-

--- End Of Report ---

Figure S77. HRESIMS spectrum of compound **20**.

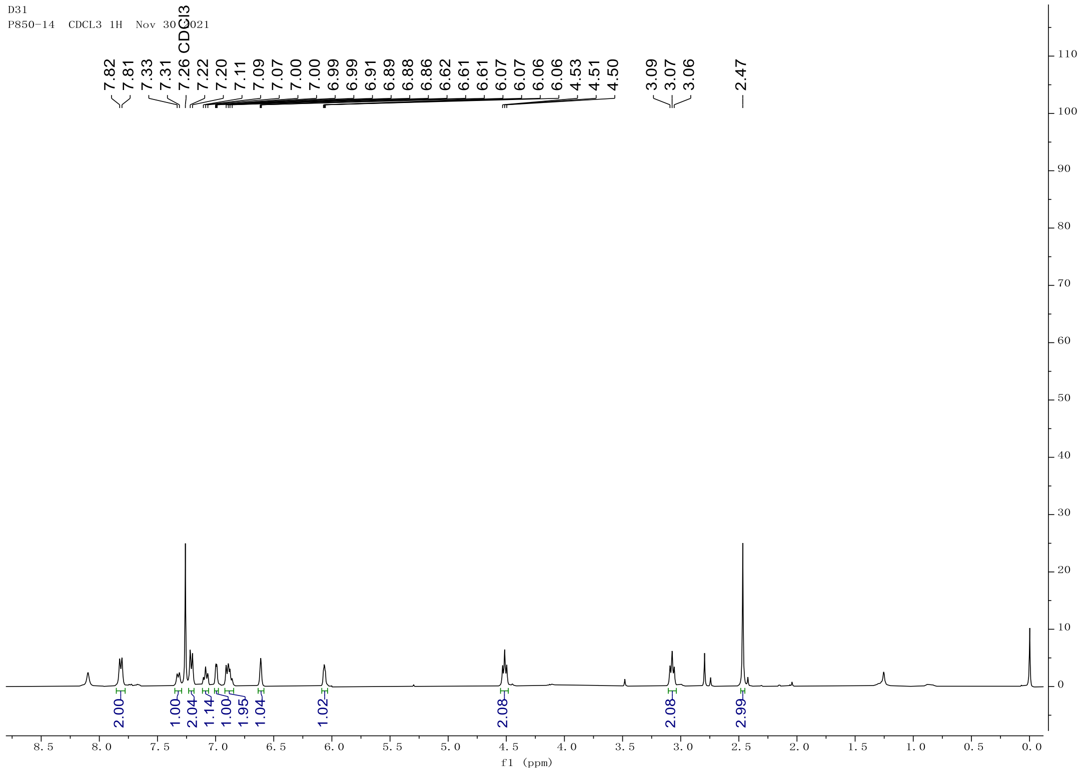

Figure S78. ^{1}H NMR spectrum of compound **20**.

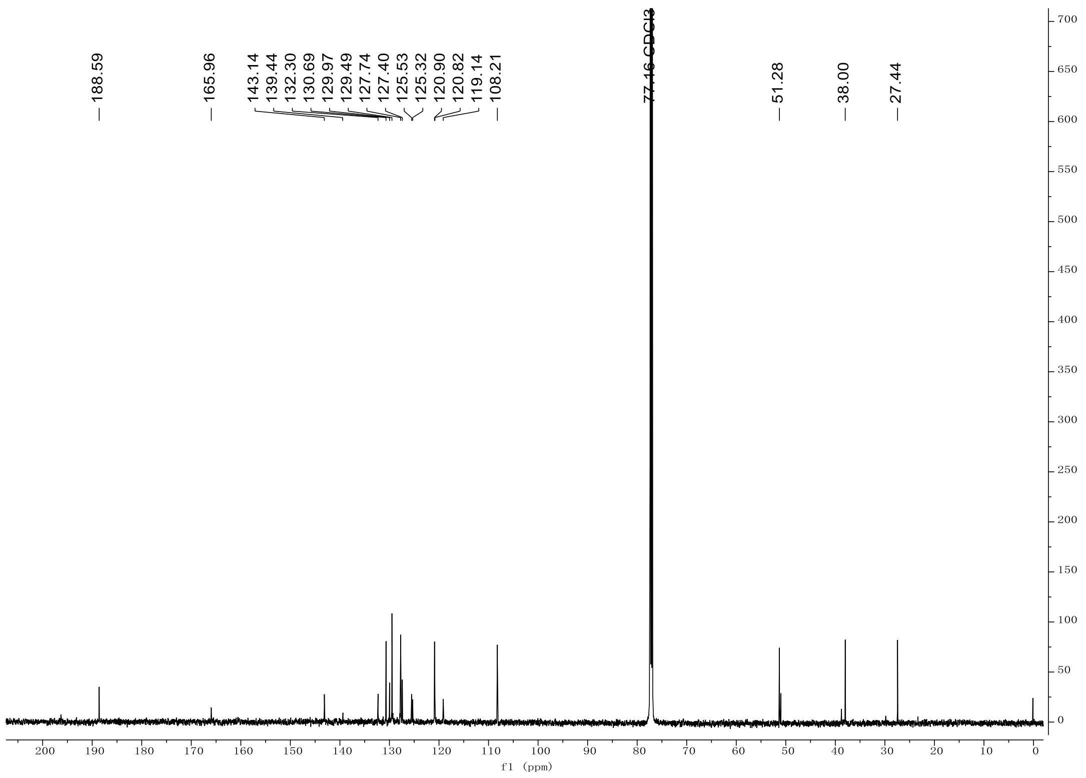

Figure S79. ^{13}C NMR spectrum of compound **20**.

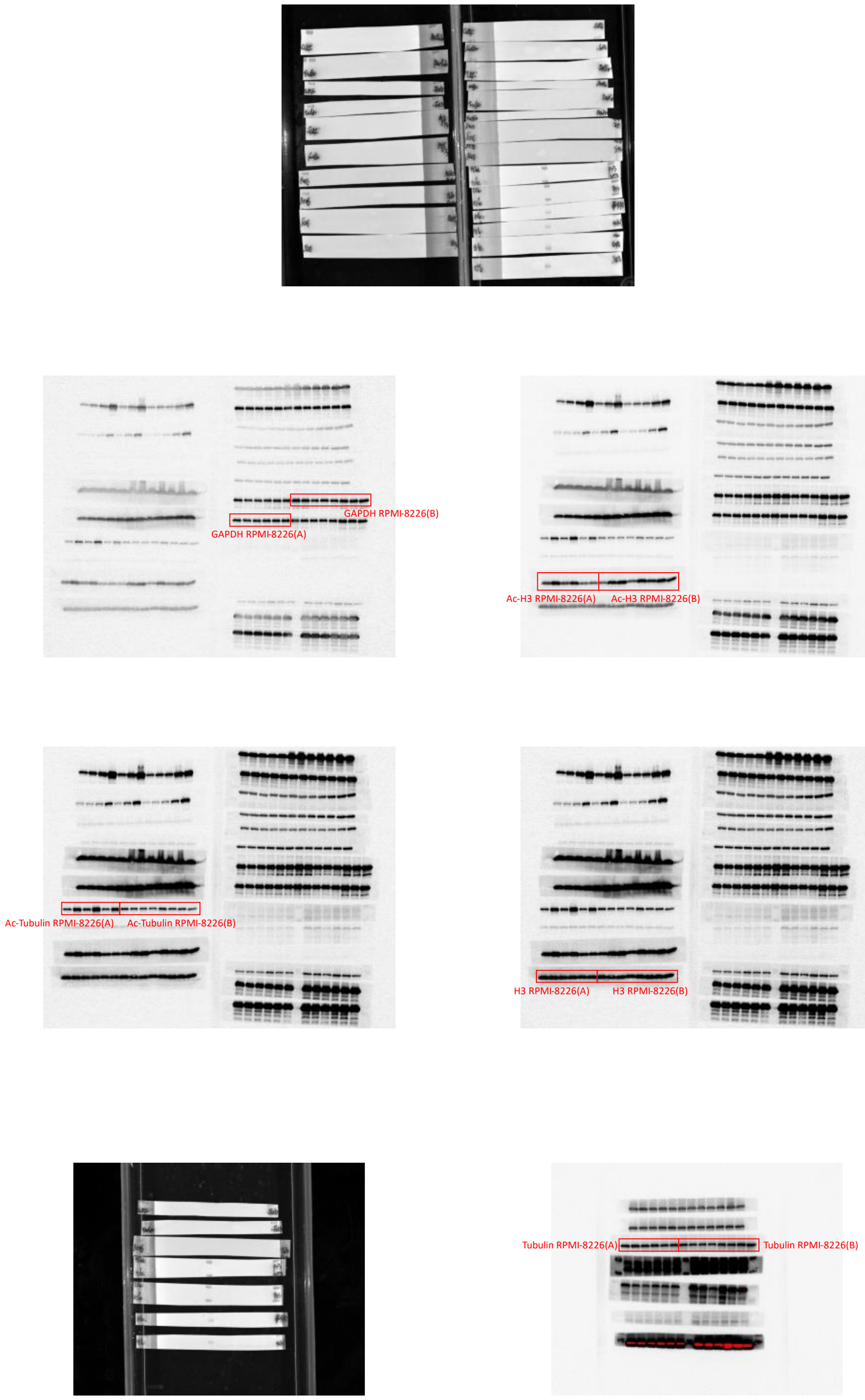

Figure S80. The whole un-cropped images of the original western blots in Figure 3.

EV	Cell No.	Cell name	Locus names									Figures
			D5S818	D13S317	D7S820	D16S539	VWA	TH01	AM	TPOX	CSF1PO	
	Query (RPM18226)		*11,13*	*11,11*	*9,10*	*9,9*	*16,18*	*8,8*	*X,Y*	*8,11*	*12,12*	
1.00(36/36)	248	COLO-677	11,13	11,11	9,10	9,9	16,18	8,8	X,Y	8,11	12,12	-
1.00(36/36)	CCL-155	RPMI 8226	11,13	11,11	9,10	9,9	16,18	8,8	X,Y	8,11	12,12	-
1.00(36/36)	IFO50013	RPMI 8226	11,13	11,11	9,10	9,9	16,18	8,8	X,Y	8,11	12,12	-
1.00(36/36)	JCRB0034	RPMI8226	11,13	11,11	9,10	9,9	16,18	8,8	X,Y	8,11	12,12	-

EV	Cell No.	Cell name	Locus names									Figures
			D5S818	D13S317	D7S820	D16S539	VWA	TH01	AM	TPOX	CSF1PO	
	Query (HCT-116Auc-		*10,11*	*10,12*	*11,12*	*11,13*	*17,22*	*8,9*	*X,Y*	*8,9*	*7,10*	
1.00(36/36)	CCL-247	HCT 116	10,11	10,12	11,12	11,13	17,22	8,9	X,Y	8,9	7,10	-

EV	Cell No.	Cell name	Locus names									Figures
			D5S818	D13S317	D7S820	D16S539	VWA	TH01	AM	TPOX	CSF1PO	
	Query (HL-60)		*12,12*	*8,11*	*11,12*	*11,11*	*16,16*	*7,8*	*X,X*	*8,11*	*13,14*	
1.00(36/36)	3	HL-60	12,12	8,11	11,12	11,11	16,16	7,8	X,X	8,11	13,14	-
1.00(36/36)	139	PLB-985	12,12	8,11	11,12	11,11	16,16	7,8	X,X	8,11	13,14	-
1.00(36/36)	CCL-240	HL-60	12,12	8,11	11,12	11,11	16,16	7,8	X,X	8,11	13,14	-
1.00(36/36)	CRL-1964	Clone 15 HL-60	12,12	8,11	11,12	11,11	16,16	7,8	X,X	8,11	13,14	-
1.00(36/36)	CRL-2257	HL-60/MX2	12,12	8,11	11,12	11,11	16,16	7,8	X,X	8,11	13,14	-
1.00(36/36)	JCRB0085	HL60	12,12	8,11	11,12	11,11	16,16	7,8	X,X	8,11	13,14	-
1.00(36/36)	JCRB0163	HL60(S)	12,12	8,11	11,12	11,11	16,16	7,8	X,X	8,11	13,14	-
1.00(36/36)	RCB0041	HL60	12,12	8,11	11,12	11,11	16,16	7,8	X,X	8,11	13,14	-
1.00(36/36)	RCB1550	HL-60-R2	12,12	8,11	11,12	11,11	16,16	7,8	X,X	8,11	13,14	-

Figure S81. The STR identification results of the three cells were correct.

Table S1. General information about the cells used in the experiments.

Cell code	Cell line name	Cell source
RPMI8226	Human multiple myeloma cells	Nanjing Kebai Biotechnology Co., Ltd.
HL-60	Human promyelocytic leukaemia cells	Chinese Academy of Sciences Committee Type Culture Collection cell bank (Shanghai, China)
HCT-116	Human colon cancer cells	American Type Culture Collection
